# Weighted families of contact maps to characterize conformational ensembles of (highly-)flexible proteins

**DOI:** 10.1093/bioinformatics/btae627

**Published:** 2024-10-21

**Authors:** Javier González-Delgado, Pau Bernadó, Pierre Neuvial, Juan Cortés

**Affiliations:** LAAS-CNRS, Université de Toulouse, CNRS, 31400 Toulouse, France; Institut de Mathématiques de Toulouse, Université de Toulouse, CNRS, 31400 Toulouse, France; Centre de Biologie Structurale, Université de Montpellier, INSERM, CNRS, 34090 Montpellier, France; Institut de Mathématiques de Toulouse, Université de Toulouse, CNRS, 31400 Toulouse, France; LAAS-CNRS, Université de Toulouse, CNRS, 31400 Toulouse, France

## Abstract

**Motivation:**

Characterizing the structure of flexible proteins, particularly within the realm of intrinsic disorder, presents a formidable challenge due to their high conformational variability. Currently, their structural representation relies on (possibly large) conformational ensembles derived from a combination of experimental and computational methods. The detailed structural analysis of these ensembles is a difficult task, for which existing tools have limited effectiveness.

**Results:**

This study proposes an innovative extension of the concept of contact maps to the ensemble framework, incorporating the intrinsic probabilistic nature of disordered proteins. Within this framework, a conformational ensemble is characterized through a weighted family of contact maps. To achieve this, conformations are first described using a refined definition of contact that appropriately accounts for the geometry of the inter-residue interactions and the sequence context. Representative structural features of the ensemble naturally emerge from the subsequent clustering of the resulting contact-based descriptors. Importantly, transiently populated structural features are readily identified within large ensembles. The performance of the method is illustrated by several use cases and compared with other existing approaches, highlighting its superiority in capturing relevant structural features of highly flexible proteins.

**Availability and implementation:**

An open-source implementation of the method is provided together with an easy-to-use Jupyter notebook, available at https://gitlab.laas.fr/moma/WARIO.

## 1 Introduction

The function of numerous proteins is intricately linked to their conformational variability. In particular, intrinsically disordered proteins/regions (IDPs/IDRs) represent an extreme example of this phenomenon (Dyson and Wright 2005, Oldfield and Dunker 2014, Clerc *et al.* 2021, Holehouse and Kragelund 2024). Nevertheless, the conformational characterization of highly flexible systems remains a challenge. Currently, structural ensembles of disordered proteins, such as those deposited in the Protein Ensemble Database (PED) (Ghafouri *et al.* 2024) or those derived from molecular dynamics (MD) simulations, are defined by a set of atomistic models, which are hard to analyze. The structural characterization of these ensembles is often reduced to very simple descriptors, such as the radius of gyration or the relative solvent accessibility, which provide very limited structural insights and that are not necessarily related with their function. Moreover, these descriptors are averaged values over the whole ensemble, ignoring the information about their distribution. Transiently populated secondary structural elements and long-range interactions are more relevant structural descriptors. However, their identification in large atomistic ensembles is often hampered by their reduced population. New descriptors are therefore needed to represent large conformational ensembles in a compact and meaningful way.

For well-folded proteins, contact and distance maps have become fundamental tools to define their 3D-fold (Phillips 1970, Nishikawa *et al.* 1972, Tanaka and Scheraga 1975), demonstrating their suitability to identify structural domains (Rossman and Liljas 1974, Kuntz *et al.* 1976, Janin and Wodak 1983). More recently, contact maps have proven key for the development of machine-learning-based approaches for structure prediction (Zheng *et al.* 2019, AlQuraishi 2021, Jumper *et al.* 2021). A naive extension of contact and distance maps to conformational ensembles, which involves estimating contact probabilities by averaging binary contacts, has been used to describe interaction propensities in structured systems (Yuan *et al.* 2012, Mercadante *et al.* 2018, Clementel *et al.* 2022, Güven *et al.* 2023). However, in the presence of structural disorder, this approach is not appropriate. More specifically, contacts between residues that are far apart in the sequence, which may be structurally or functionally important but occur with low probability, remain undetectable in these representations. Similarly, scarcely populated structural motifs are diluted in the average contact/distance maps. This phenomenon is illustrated in Fig. 3a, which displays the average contact map for a conformational ensemble of a 27-residue-long IDR in CHCHD4, one of the proteins used as an example in this study (see Section 3). This representation only highlights contacts around the diagonal of the matrix, while long-range contacts that appear at low frequency remain undetected. Consequently, the characterization of conformational ensembles on the basis of contacts represents a nontrivial task that requires novel approaches integrating the statistical nature of flexible proteins.

In order to overcome the above-described limitations, we propose a new approach that, while exploiting the power of contact maps, is adapted to the structural variability of highly flexible proteins. More precisely, we introduce the concept of *weighted family of contact maps* to characterize a conformational ensemble, by representing its structural diversity through a set of short- and long-range contact patterns that appear at a given frequency. This is done by first applying a well-suited clustering algorithm that unravels the underlying conformational variability of the protein and then characterizing such distribution through its representative network of contacts.

Clustering conformations of highly flexible proteins is a challenging problem since their conformational space can be considered as a high-dimensional manifold with non-Euclidean geometry. In this regard, nonlinear dimensionality reduction algorithms, such as t-SNE (van der Maaten and Hinton 2008) and UMAP (McInnes *et al.* 2020), are very attractive to disentangle features embedded in high-dimensional data (Diaz-Papkovich *et al.* 2019, Sakaue *et al.* 2020). Besides, their incorporation into clustering algorithms has shown remarkable efficiency (Becht *et al.* 2018, Allaoui *et al.* 2020, Dorrity *et al.* 2020, Grootendorst 2022). This idea has been recently exploited to analyze results of MD simulations (Appadurai *et al.* 2023, Conev *et al.* 2023). However, in these works, conformations were usually featured by descriptors such as atom coordinates or backbone torsion angles, and compared using root-mean-square deviation (RMSD) (Rao and Rossmann 1973, Maiorov and Crippen 1994), whose suitability to compare unfolded conformations is questionable. Here, we propose to use contacts also to feature conformations prior to clustering. However, unlike current approaches that use an arbitrary threshold, we define contacts as a continuous weight function that acts as a proxy of the interaction between residue pairs. Importantly, this weight function depends on the amino-acid types, their separation in the sequence, their Euclidean distance and their relative orientation. We show that the appropriate combination of these parameters in the contact definition is crucial for the detection of transient structural features within large ensembles. Then, clustering can be performed on the conformational space featured with contact-based information using HDBSCAN (Campello *et al.* 2013), passing through a low-dimensional UMAP projection. In addition to the contact pattern, several descriptors associated to each cluster can be derived, such as secondary structure propensities, average radius of gyration and end-to-end distances.

**Figure 1.**
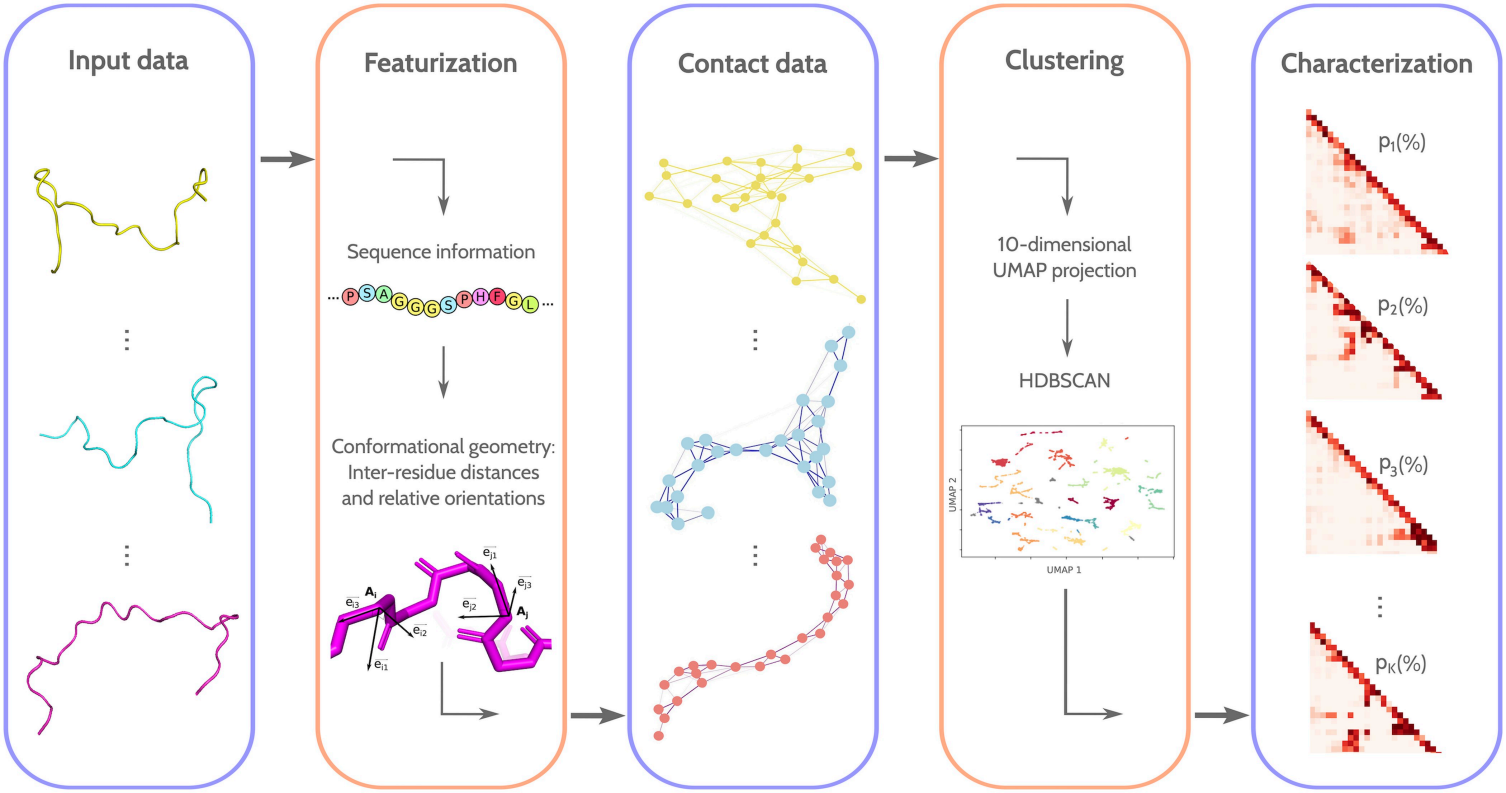
Overview of WARIO pipeline implementation. The method takes a conformational ensemble as input. For each conformation, inter-residue distances are computed, considering the sequence and the relative orientation of residue pairs. Using this information, a proxy for the inter-residue contacts within the conformation is calculated, and the resulting values are used as structural descriptors. Subsequently, the conformations are classified using the contact-based descriptors through a clustering algorithm that incorporates a projection into a low-dimensional space. Finally, each cluster is represented by an average contact map describing the inter-residue interactions within the corresponding group, along with their frequency within the ensemble. This weighted family of contact maps characterizes the conformational ensemble.

The pipeline with the stages corresponding to the actual implementation of the method, which we named WARIO, is illustrated in Fig. 1. This original approach provides a compact and meaningful representation of conformational ensembles of flexible proteins, from which functionally important structural features can be easily identified.

## 2 Materials and methods

### 2.1 Description of intramolecular contact as a sequence and orientation-dependent continuous function

Conventionally, a contact between a pair of residues is defined as a binary indicator when the Euclidean distance between their $C_{\alpha }$ (or $C_{\beta }$) atoms is less than a certain threshold, typically set between 6 Å and 12 Å (Newton *et al.* 2022). This indicator is universal for every pair of residues regardless of their identities, positions in the sequence or relative orientation. However, it is known that these parameters influence inter-residue interactions. Indeed, when looking at how Euclidean distances are distributed in high-resolution structures, we observed that they concentrate around values that are strongly dependent on the amino-acid identities and their sequence distance (from now on, we will use the term *range* to designate the sequence distance in number of amino acids). Furthermore, interacting residues present preferred relative orientations that clearly manifest for short-range contacts. Consequently, an accurate contact descriptor must integrate both sequence and geometric information, and avoid universal binary indicators that, as we show here, yield a substantial loss of structural information.

**Figure 2.**
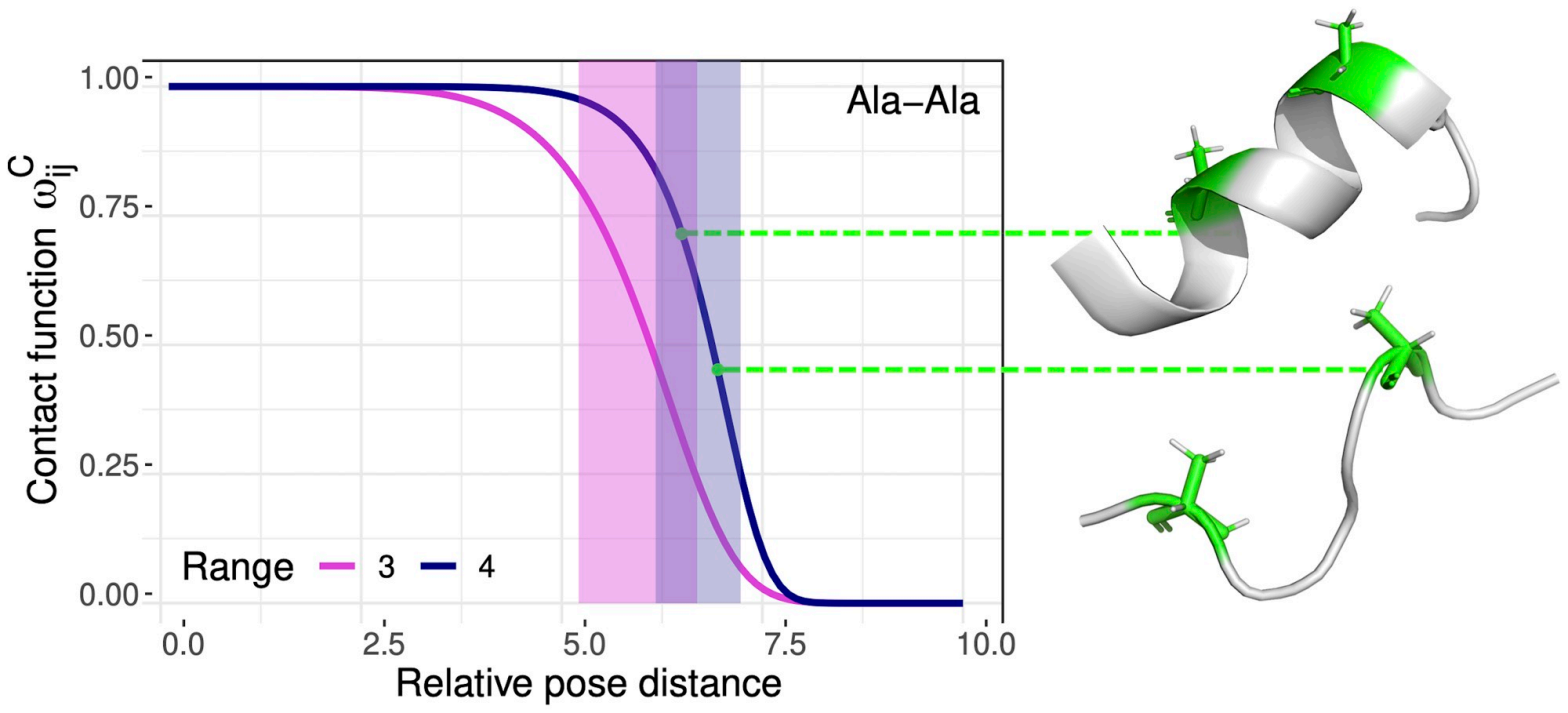
Contact function for Ala-Ala residue pairs at ranges 3 and 4. The growth of each curve is concentrated within the so-called contact interval, which is marked by a band colored magenta and blue for ranges 3 and 4, respectively. When inter-residue Euclidean distances remain equal, the contact function yields higher values for relative orientations that more closely resemble the preferred ones observed in the structural database. On the right, two Ala-Ala pairs at range 4 with equal $C_{\beta }$-$C_{\beta }$ distances of 7.4 Å. The configuration on the top has a higher contact value, indicating its closer alignment to the preferred orientation of both alanine residues at range 4.

Here, we redefine *contact* as a continuous function, taking values in the interval [0, 1], that integrates sequence information and the relative orientation between the interacting residues. To do so, we followed the steps briefly explained below. Details are provided in Supplementary Section S1 of the Supplementary Information (SI). The contact function was defined based on the analysis of 15 177 experimentally determined high-resolution (<2 Å) structures of protein domains extracted from the SCOPe 2.07 release (Chandonia *et al.* 2019), which we will refer to as the *structural database*. The first step corresponds to the identification of Euclidean contact distance maxima in the structural database, which depend on the identity and range of the two residues. These maxima are used to define the so-called *Euclidean contact interval*, which represents a fuzzy boundary below which the interaction between residues is meaningful. We observed that, for Euclidean distances below its upper limit, preferred orientations clearly stand out in the structural database. Once again, they depend on the residue identities and range and they are more clearly observed for short-range contacts (see Supplementary Fig. S3). These preferred orientations need to be combined with the Euclidean distance in a suitable way. In this respect, we ask the orientation contribution: (i) to be negligible for large values of the Euclidean distance, and (ii) to contribute to enhance the proxy for contact only if it is close to the specific preferred orientation of the residue pair. Conditions (i) and (ii) yield the definition of the so-called *relative pose distance*, which equals the Euclidean distance for large values or if the contact is long range, and progressively reduces the Euclidean distance as the relative inter-residue orientation approaches the preferred one. Note that in the present context, the term *pose* refers to the position and orientation of the amino acid in the 3D space. The relative pose distance is explicitly constructed as a continuous function combining the Euclidean distance and the deviation from the preferred orientation. Its functional form is parameterized by the identities and the range of the corresponding residue pair. Finally, the relative pose distance is transformed into a proxy for contact taking values in [0, 1], which we refer to as *contact function*. It is a decreasing function of the relative pose distance, parameterized by the sequence information. The growth of the curve is concentrated within a specific interval, which was analogously determined through the analysis of the relative pose distance distribution in the structural database. This is illustrated with an example in Fig. 2, where the contact function is depicted for Ala-Ala residue pairs at different range values.

The redefinition of contact as a continuous function in [0, 1], depending on the relative position, orientation, and sequence information, proves to be essential for an appropriate characterization of the structural dynamics of flexible proteins, as shown in Supplementary Section S2 in SI.

### 2.2 Clustering pipeline and ensemble characterization

The clustering method applied in WARIO relies on the contact function defined above. Conformations are featured by the contact function values for every pair of residues along the sequence. Consequently, an ensemble corresponding to a protein of length L and having n conformations is described by the n×L(L−1)/2 matrix:

(1) $$W_{C}=\left(\begin{array}{ccccc} \omega_{12;1}^{C} & \cdots & \omega_{ij;1}^{C} & \cdots & \omega_{(L-1)L;1}^{C}\\ \omega_{12;2}^{C} & \cdots & \omega_{ij;2}^{C} & \cdots & \omega_{(L-1)L;2}^{C}\\ \vdots & & \vdots & & \vdots \\ \omega_{12;n}^{C} & \cdots & \omega_{ij;n}^{C} & \cdots & \omega_{(L-1)L;n}^{C} \end{array}\right),$$

where $\omega_{ij;k}^{C}$ denotes the value of the contact function for residues i,j∈{1,…,L} in the kth conformation, for k∈{1,…,n}. Note that this formulation is equivalent to consider each conformation as a graph, as it has been previously done in related methods such as RING (Martin *et al.* 2011, Clementel *et al.* 2022). Here, the set of nodes is given by the set of residues and every pair of residues i, j is linked by an edge with a weight $\omega_{ij;k}^{C}$. This procedure is depicted in Fig. 1. It should be noted, however, that the graphical representation is merely an alternative visualization of the data, and that our methodology does not rely on graph theory.

The clustering method performed on the contact function matrix (1) is based on the combination of a dimensionality reduction technique with an efficient clustering algorithm, similarly to state-of-the-art approaches (Appadurai *et al.* 2023, Conev *et al.* 2023). Here, we opt for UMAP (McInnes *et al.* 2020) to first embed the data (1) into a low-dimensional space, as this strategy has been shown to improve the performance of several clustering algorithms (Allaoui *et al.* 2020). Besides, the use of UMAP has demonstrated its ability to preserve the topology of the high-dimensional data and efficiently reveal population structure (Dorrity *et al.* 2020, Sakaue *et al.* 2020, Diaz-Papkovich *et al.* 2021). In this work, we set to 10 the dimension of the low-dimensional UMAP space based on empirical analyses, although the user can change this parameter in the provided implementation. The HDBSCAN clustering algorithm (Campello *et al.* 2013), which we consider to be one of the best-performing density-based techniques, in then applied to the embedding. One of its practical advantages is that it only requires as input parameter the minimum cluster occupancy and automatically selects the number of classes. This is suitable for our implementation, as the practitioner might have more intuition of the desired “resolution” of the characterization through the setting of a minimum number of conformations rather than through the direct choice of a number of classes. Details on UMAP and HDBSCAN are provided in SI.

Once the clustering is performed, each class is characterized through a cluster-specific contact map. Let K be the number of retrieved clusters and $C_{k}\subset \{1,\ldots ,n\}$ be the subset of conformations constituting the k-th cluster, for k∈{1,…,K}. Of course, $C_{k}\cap C_{k^{\prime }}=\emptyset$ for all $k\neq k^{\prime }$. Keeping the notation of (1), we define the k-th *cluster-specific* ω*-contact map* as the (L−1)×(L−1) matrix:

(2) $${\bar{W}}_{C_{k}}={\left(\frac{1}{\left|C_{k}\right|}\sum_{l\in C_{k}}\omega_{ij;l}^{C}\right)}_{ij}\;\text{for}\;i<j\in \{1,\ldots ,L\},$$

where $\left|C_{k}\right|$ denotes the cardinality of $C_{k}$. The matrix (2) is the average of all the rows in (1) that belong to the k-th cluster, represented in a matrix form. Its entries are the cluster averages of the contact function values for every pair of residues along the sequence, and it accounts for the contact patterns that dominate the cluster. A weight $p_{k}=\left|C_{k}\right|/n$ can be assigned to each matrix (2) based on the cluster occupancy proportion. This allows us to define the *ensemble characterization* as the K-tuple of weighted cluster-specific ω-contact maps:

(3) $$E=\left(\left({\bar{W}}_{C_{1}},p_{1}\right),\ldots ,\left({\bar{W}}_{C_{K}},p_{K}\right)\right),$$

which provides a compact characterization of inter-residue interactions in the ensemble.

Each cluster of conformations can be analyzed *a posteriori* on the basis of additional descriptors. Here, we propose to evaluate the secondary structure propensities based on the structural classification provided by DSSP (Kabsch and Sander 1983) and to compute the cluster average radius of gyration. Other descriptors can be easily added using methods implemented in tools such as SOURSOP (Lalmansingh *et al.* 2023).

### 2.3 Software availability

WARIO has been implemented in Python, and can be executed through an easy-to-use Jupyter Notebook. The open-source code, along with detailed installation and use instructions, is available at https://gitlab.laas.fr/moma/WARIO.

## 3 Results

We have used WARIO to characterize ensembles of three highly flexible proteins containing different levels of structure. We applied the pipeline described in Section 2 to ensembles extracted from long MD trajectories. Details of these simulations can be found in the original articles. Through these examples, we demonstrate the ability of our approach to localize scarcely populated structural patterns, including secondary structural elements and transient long-range contacts. We also compared WARIO’s contact-based clustering method with other approaches, highlighting its unique ability to cluster structural patterns that often remain unidentified by other strategies.

### 3.1 Characterization of the N-terminal region of CHCHD4

CHCHD4 (coiled-coil-helix-coiled-coil-helix domain containing 4) plays a crucial role in the import of intermembrane space-targeted proteins (Hofmann *et al.* 2005, Fischer *et al.* 2013). Only the structure of the folded domain of CHCHD4 (residues 45–109) has been experimentally resolved (Banci *et al.* 2009). However, the interaction with most of its clients exclusively involves the intrinsically disordered N-terminal region (27 residues) (Hangen *et al.* 2015), which is the fragment analyzed here.

**Figure 3.**
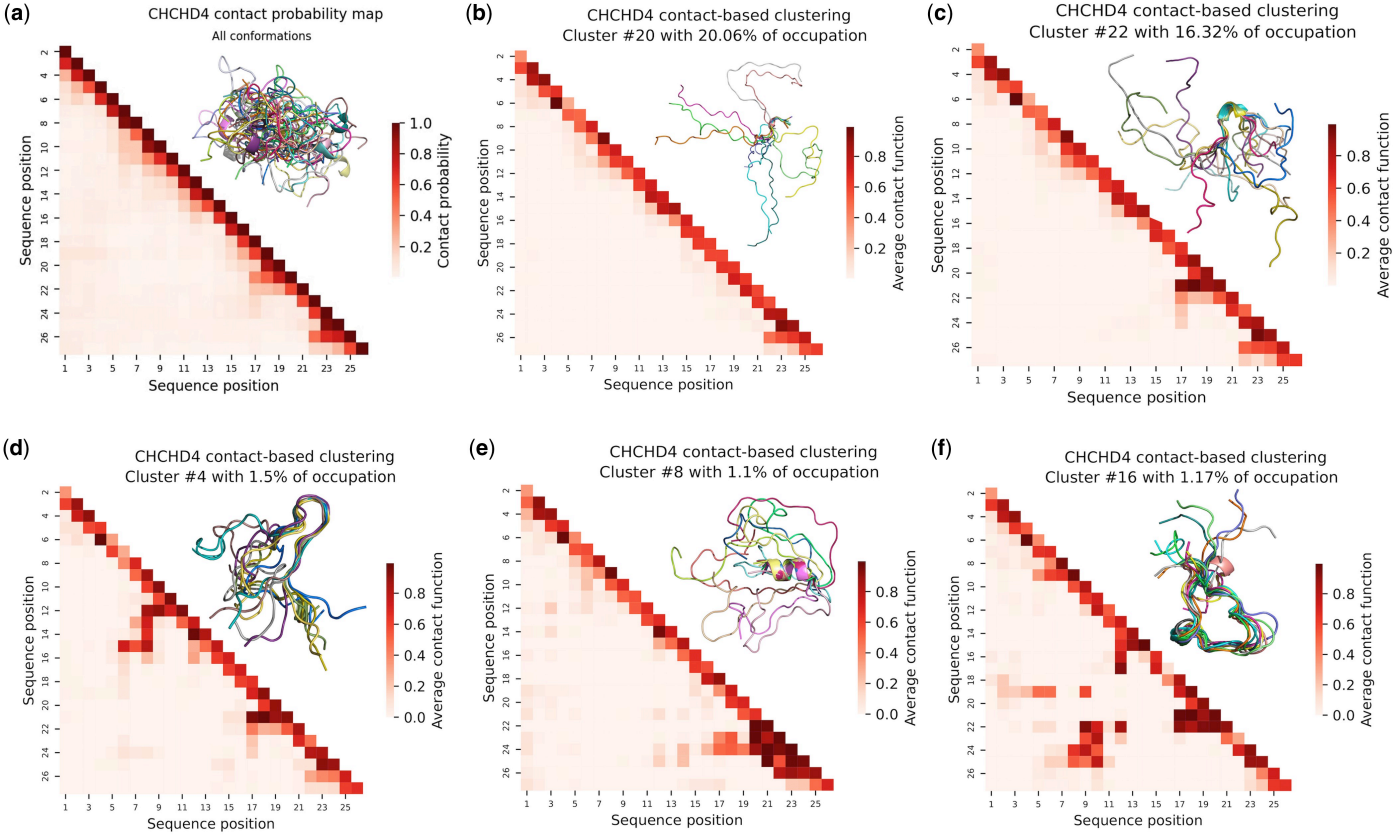
Structural characterization of CHCHD4. (a) Contact probability map for the conformational ensemble of CHCHD4. Each contact probability is estimated as the proportion of contacts between a pair of residues, considering a 8 Å distance threshold between the $C_{\beta }$ atoms ($C_{\alpha }$ for glycine). In the upper triangle, 10 randomly selected conformations from the ensemble. (b–f) Cluster-specific ω-contact maps (2) for five clusters of CHCHD4. Panels (b) and (c) correspond to the two most populated groups encompassing 20.06% and 16.32% of the conformations. In each upper triangle, 10 randomly selected conformations from the corresponding cluster and aligned on residues exhibiting off-diagonal contact patterns. Note that the cluster numbering is arbitrary and it is not related with its population.

A conformational ensemble of the disordered N-terminal region of CHCHD4, encompassing n=100050 conformations, was generated from 50 independent MD trajectories of 200 ns each. Data are publicly available in Zenodo, at https://doi.org/10.5281/zenodo.10777457. Using a minimum cluster size of 1% of the total number of conformations, WARIO identified 23 clusters with different levels of occupancy. The two most populated clusters contained approximately 20% and 16% of the conformations, while the remaining 21 clusters only represented 1%–3% of them. The overall cluster distribution can be visualized through the projection to a 2D UMAP space (see Supplementary Section S4.1 in the SI). The complete family of ω-contact maps for CHCHD4 as well as the secondary structure propensities and average radius of gyration for all clusters are presented in the SI. The average ω-contact map of the two most populated clusters showed the presence of some local structure at the C-terminus of the chain and a complete absence of long-range contacts (Fig. 3b and c). Interestingly, all the remaining, low-occupied conformational clusters presented more specific structural features (Fig. 3d–f). For instance, two clusters containing 1.5% and 1.1% of the population presented a turn from residues 6 to 15 and a short α-helix, respectively (panels (d) and (e) in Fig. 3). Indeed, the clusters provided by WARIO group together conformations that exhibit the same secondary structure motifs. As shown in Supplementary Section S2 of the SI, this is due to the proper incorporation of relative orientation in the definition of inter-residue contacts. Another low populated (1.17%) cluster displayed a well-defined long-range contact between the central and the C-terminal region of the peptide (Fig. 3f). When analyzing the average radius of gyration for all the identified clusters, a large difference was observed between the two most populated ones, with values of 15.36 Å and 13.98 Å, respectively, and the others, presenting values around 10–12Å. This observation substantiates the presence of long-range contacts in the majority of the low-populated clusters. This analysis demonstrates the ability of WARIO to identify and localize scarcely populated structural patterns from large ensembles.

### 3.2 Comparison of WARIO’s featurization with other methods

**Figure 4.**
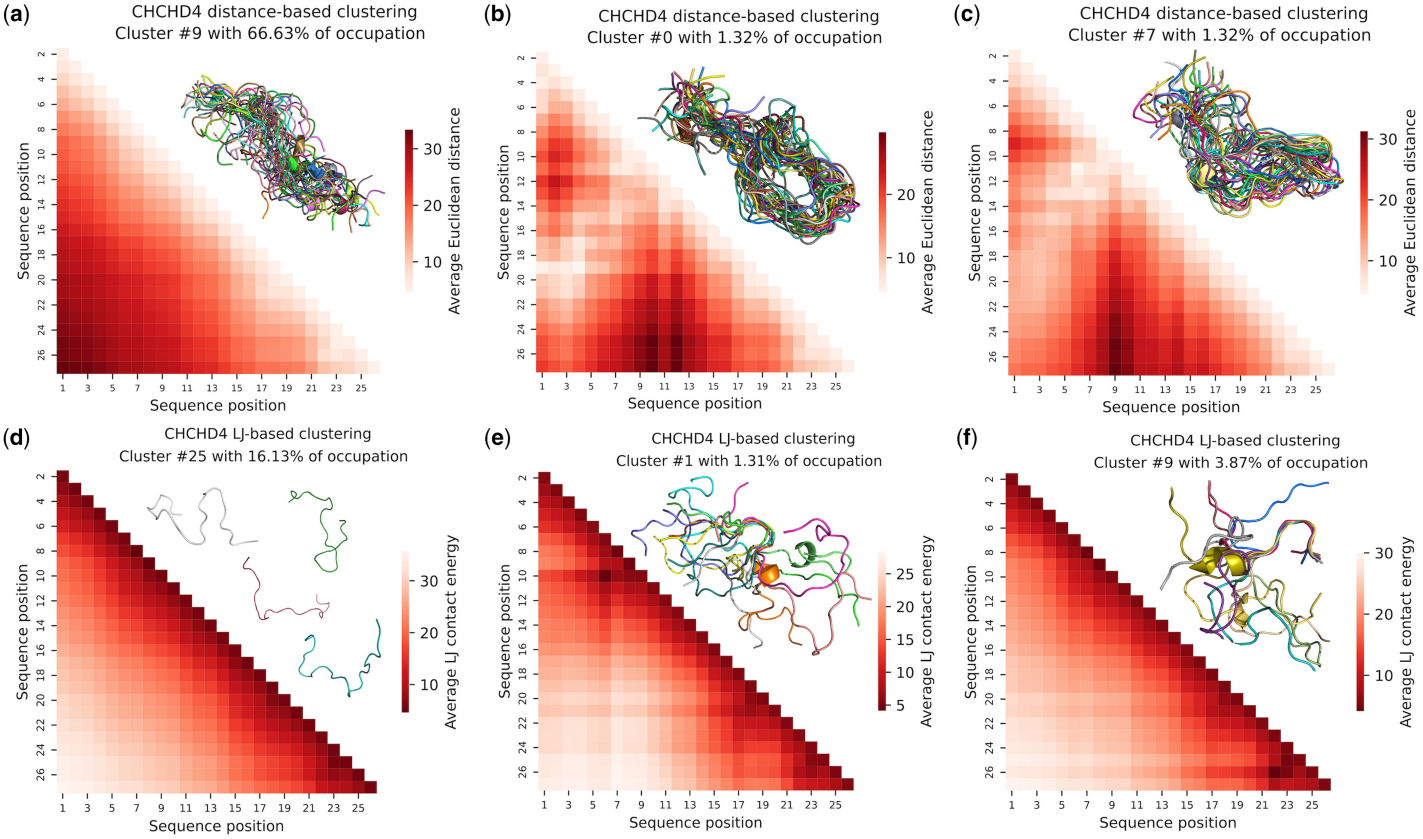
Comparison of WARIO with other clustering approaches. (a–c) CHCHD4 cluster-specific average distance maps after applying the UMAP + HDBSCAN pipeline to the set of conformations featured by all Euclidean inter-residue distances. In each upper triangle, 30 randomly selected CHCHD4 conformations from the corresponding cluster and aligned on all residues are displayed. (d–f) CHCHD4 cluster-specific Lennard-Jones contact maps after applying the UMAP + HDBSCAN pipeline to the set of conformations featured by all inter-residue LJ interaction potentials. In each upper triangle of (e, f), 10 randomly selected CHCHD4 conformations from the corresponding cluster and aligned on residues with low average contact energy values. In the upper triangle of panel (a), corresponding to the most populated cluster, four nonaligned randomly selected conformations from the group are displayed. Note that the cluster numbering is arbitrary and it is not related with its population.

We compared the clusters obtained using WARIO for CHCHD4 with those provided by two existing approaches based on pairwise distances and inter-residue Lennard-Jones contact energies. In distance-based methods, structural data are featured by Euclidean distances between residue pairs. This metric has proven its suitability for detecting structural differences between ensembles of flexible proteins (Lazar *et al.* 2020, González-Delgado *et al.* 2023b). It is frequently used together with dimensionality reduction and clustering algorithms to analyze conformational ensembles (Lowry *et al.* 2008, Appadurai *et al.* 2023, Conev *et al.* 2023). However, when employed to characterize the structure of a highly flexible protein, the use of Euclidean distances does not show the same efficacy as when used for comparative purposes. This is explained because the inter-residue Euclidean distances for the whole protein primarily account for the global structure of the conformation, and are less sensitive to transient interactions. To illustrate this, we applied the UMAP + HDBSCAN pipeline to the structural data featured with pairwise Euclidean distances between all $C_{\beta }$ atoms ($C_{\alpha }$ for glycines) to characterize the CHCHD4 MD ensemble. This strategy retrieved 10 clusters, among which one contained the 67% of conformations. Note that WARIO retrieved 23 clusters for the same ensemble and that the two most occupied clusters contained approximately 20% and 16% of the conformations. Figure 4a–c displays the average distance maps for the three most occupied clusters, together with 30 conformations randomly drawn from each cluster and aligned using all residues. As the UMAP + HDBSCAN pipeline is fed with all the pairwise distances, clusters tend to group conformations having similar global shapes and do not necessarily group them according to the presence of structural motifs or long-range contacts. As a consequence, the structural clusters yield much broader contact maps when compared to the results yielded by WARIO. Therefore, distance-based methods do not seem adapted to identify scarcely populated states diluted in a conformationally diverse ensemble.

The use of an inter-residue Lennard-Jones (LJ) interaction potential to feature individual conformations has been recently reported (Appadurai *et al.* 2023). The capacity to capture interactions within the chain makes this strategy similar to our continuous contact function. In order to implement the LJ potential, we repeated the same strategy as in the previous distance-based analysis but featuring each conformation k∈{1,…,n} by the vector $\left(V_{12;k},\ldots ,V_{ij;k},\ldots ,V_{L(L-1);k}\right)$, where $V_{ij;k}$ is the inter-residue LJ contact energy between residues i and j in the k-th conformation. The explicit form of the interaction potential is given in (Clementi *et al.* 1999, Eqs. 1–3).

After classifying the LJ interaction matrices with the UMAP + HDBSCAN pipeline, one predominant cluster was retrieved containing around 16% of conformations, together with 25 other groups with populations ranging from 1% to 5% (Fig. 4d–f), similarly to the number of clusters retrieved with WARIO. The regions of these maps displaying low energy values indicate pairs of residues with more likely interactions. Although this representation is more diffuse than that based on contact functions implemented in WARIO (see Fig. 3), it still allows for the identification of cluster-specific interaction patterns. When looking at the most populated cluster (Fig. 4d), an interaction map with low energy values near the diagonal that steadily increases towards the interior of the matrix was observed, and no local contact or long-range interaction could be identified. This contradicts the inter-residue interactions observed for some randomly selected conformations of the cluster, as shown in Fig. 4d. The inspection of less populated clusters indicates that LJ-based interaction maps are more diffuse than the continuous-contact ones and that the derivation of a specific structural features from these maps is less straightforward (Fig. 4e and f). In order to exemplify this last observation, we searched among the LJ-based clusters one presenting a helical motif at residues 21–24, as detected by WARIO (Fig. 3e). For this, we identified three LJ maps presenting energy minima at the C-terminus (Fig. 4f, Supplementary Fig. S9c and e). However, the secondary structure analysis of these three clusters displayed a negligible α-helical propensity for residues 21–24 (Supplementary Fig. S9b, d, and f), indicating that the LJ-based description of contacts produces structurally ill-defined clusters. The distance and LJ-based featurizations, along with classical binary contacts, are compared to WARIO using the adjusted Rand index in Supplementary Section S3.2 of the SI.

### 3.3 Characterization of Huntingtin Exon-1 and TAR DNA-binding protein 43

We applied WARIO to characterize conformational ensembles of more challenging systems. Due to length constrains, detailed descriptions are presented in Supplementary Section S4 of SI.

The *huntingtin exon-1 (HTTExon-1)*, which contains a polyglutamine tract, poly-Q, is the main toxic agent in Huntington’s disease (Saudou and Humbert 2016). A 20-microsecond molecular dynamics (MD) simulation of HTTExon-1 (Elena-Real *et al.* 2023) with a 46 glutamines and 5 prolines was analyzed using WARIO, revealing 43 low-population structural clusters, each representing 1%–3% of conformations. Cluster-specific contact maps and secondary structure analyses identified a systematic extension of helical structures within the poly-Q tract. By refining the clustering resolution, WARIO detected scarcely populated intramolecular contacts, such as a β-sheet formation in 0.2% of the conformations, demonstrating the ability of the method to identify critical structural features with extremely low populations.

The structure of *TAR DNA-binding protein 43 (TDP-43)*, associated with amyotrophic lateral sclerosis and frontotemporal dementia (Cohen *et al.* 2011), was studied through all-atom MD simulations at 100 and 300 mM NaCl to understand its phase separation behavior (Mohanty *et al.* 2024). WARIO analysis of these simulations elucidated intricate interdomain interactions. At low ionic strength (100 mM NaCl), TDP-43 showed complex interaction networks involving its N-terminal domain (NTD), disordered regions (IDR1 and IDR2), and RNA-recognition motifs. Upon increasing the ionic strength at 300 mM NaCl, some interactions, such as the L1-RRM2 contact, remained unaltered, while others, such as the IDR1-IDR2 contact, disappeared. This analysis revealed the capabilities of WARIO in providing insights into the protein behavior under different experimental conditions.

## 4 Discussion

The proposed method provides a compact and meaningful characterization of conformational ensembles through a weighted family of contact maps. The idea of using a graph-based characterization built from contact information to investigate biomolecular ensembles has been previously proposed (Clementel *et al.* 2022). However, due to the enormous structural variability of highly flexible proteins and to the sparsity of most long-range contacts, the average probability of residue-residue contacts is not a suitable structural descriptor. To account for the complex nature of the contact distribution, WARIO first unravels the most determinant interaction patterns that characterize the ensemble and then represents them as easily interpretable cluster-specific contact maps, with associated weights accounting for their population. A key point of this procedure is a novel definition of contact that integrates the chemical nature of the residues involved, their distance along the sequence and their relative orientation. Taking into account the relative orientation of interacting residues is essential to correctly identify scarcely populated structural motifs. Note that these motifs are often the anchoring points for biomolecular assemblies, where they can modulate the thermodynamics and kinetics of recognition events (Tompa *et al.* 2015, Davey 2019). In the current implementation of WARIO, the relative orientation is not considered for long-range interactions. Indeed, our analyses of high-resolution protein structures did not show clearly preferred orientations for residue pairs with a distance greater than four along the sequence. Despite this, in all our examples, very clear contacts between residues far apart in the sequence were detected. Importantly, WARIO was able to cluster these conformations based primarily on the presence of these long-range contacts. This is possible thanks to the use of contact information to feature conformations instead of global descriptors based on atomic coordinates, which we showed to be less effective to derive structurally meaningful clusters.

It is important to emphasize that descriptors based on contact information hold particular significance for the investigation of disordered proteins. Indeed, they can be directly associated to experimental data reporting on local and global structural information obtained by Nuclear Magnetic Resonance (NMR) (Milles *et al.* 2018), Small-Angle X-ray Scattering (SAXS) (Bernadó and Svergun 2012), single molecule Förster Resonance Energy Transfer (smFRET) (Chowdhury *et al.* 2023), Electronic Paramagnetic Resonance (EPR) (Jeschke 2013) or from mutational studies (Pounot *et al.* 2024). In contrast, the use of atomic coordinates, the most standard descriptor for rigid protein structures, is less suitable in this context, since the experimental techniques providing such information, namely X-ray crystallography and cryo-electron microscopy, are not applicable to highly flexible systems.

The proposed ensemble characterization approach relying on contact-based clustering is clearly defined and easy to interpret. Nevertheless, it strongly depends on the minimum cluster size used by HDBSCAN. The output dependence on hyper-parameters is an intrinsic and unavoidable property of all clustering algorithms. However, in our pipeline, the minimum cluster size is easily interpretable as the desired resolution for the characterization (3). The smaller the size, the finer the classification, allowing the detection less frequent contact patterns, although too high resolution could result in redundancy. The choice of the clustering resolution should be made based on the practitioner’s needs, and its readjustment can be envisioned depending on the results. It is important to emphasize that, in general, there is no “true number of clusters,” and all classification algorithms aim at representing the diversity of the conformational states rather than revealing a nonexisting underlying partition. An effective solution to deal with the dependence on the minimum cluster size would be to apply statistical techniques, providing evidence of the differences between the clusters obtained at different resolutions and evaluate whether several clusters can be merged into a larger one, or vice versa. This problem is a growing field of research referred to as post-clustering inference. However, these methods are highly dependent on the type of algorithm used for clustering and on the interdependence of the observations and descriptors employed. Despite recent advances (Gao *et al.* 2022, Chen and Witten 2023, González-Delgado *et al.* 2023a), their application to the evaluation of WARIO results remains to be explored.

In the present study, we have applied WARIO to single-chain trajectories, but its range of applications could be easily extended to study large biomolecular multi-chain complexes with different levels of disorder, and to ensembles containing several copies of the same or different molecules (Galvanetto *et al.* 2023, Guseva *et al.* 2023). Note however that the current implementation of WARIO operates in an all-atom representation of the protein backbone. This is required for the definition of the residue-specific reference frame and, therefore, for the integration of relative orientation into the contact function. The adaptation of WARIO to coarse-grained (CG) models would be extremely valuable in the present context of continuous improvement of force-fields with the aim of investigating condensed states of phase separating systems (Tesei *et al.* 2021, Rizuan *et al.* 2022). Until this extended version of WARIO is available, interested users can rely on algorithms to transform CG into all-atom models such as cg2all (Heo and Feig 2024) or integrated tools in MD simulation packages.

As illustrated through the above-presented examples, WARIO can be easily applied to analyze the structural behavior of highly flexible protein from conformational ensembles produced by MD simulations. Furthermore, WARIO can also be applied to ensembles generated by statistical sampling methods (Bernadó *et al.* 2005, Estaña *et al.* 2019, Teixeira *et al.* 2022), as we have shown for the N-tail protein from Sendai virus (Jensen *et al.* 2008) (see Supplementary Section S5 in SI).

Importantly, the results provided by WARIO can also help to understand structural effects of mutations or environmental changes, as shown with the analysis of TDP-43 in two ionic strengths. An interesting direction for future work could be to exploit WARIO’s clustering capabilities to build Markov State Models (MSM) from MD simulation (Prinz *et al.* 2011, Sisk and Robustelli 2024), in order to study the kinetic properties of intrinsically disordered proteins. Nevertheless, we believe that WARIO’s greatest potential lies in its coupling with machine-learning (ML) methods for the prediction of the conformational behaviour of disordered chains in solution. Some recent studies have shown the potential of ML method to predict structural properties of IDPs/IDRs directly from sequence (Lotthammer *et al.* 2024, Tesei *et al.* 2024). However, these approaches are based on extremely simple structural descriptors, such as the radius of gyration and the end-to-end distance, and therefore provide very limited insights into the conformational details at the residue level. The weighted families of contact maps proposed in this work would enable the development of more accurate predictors and generative models for IDPs and IDRs.

## Supplementary Material

btae627_Supplementary_Data
