# Supplementary material for: Weighted families of contact maps to characterize conformational ensembles of (highly-)flexible proteins
Source: Bioinformatics. 2024 Oct 21;40(11):btae627. doi: 10.1093/bioinformatics/btae627 (PMC11530230; doi:10.1093/bioinformatics/btae627)

Supplementary Information

WARIO: Weighted families of contact maps to characterize conformational ensembles of (highly-)flexible proteins

Javier González-Delgado[1,2], Pau Bernadó[3], Pierre Neuvial[2] and Juan Cortés[1]

[1] *LAAS-CNRS, Université de Toulouse, CNRS, Toulouse, France.*

[2] *Institut de Mathématiques de Toulouse, Université de Toulouse, CNRS, Toulouse, France.*

[3] *Centre de Biologie Structurale, Université de Montpellier, INSERM, CNRS, Montpellier, France.*

The Supplementary Information is organized as follows:

- Section S1 details the methodology defining the complete clustering pipeline.
  - Section S1.1 starts by relaxing the threshold-based contact definition for Euclidean distances through the introduction of sequence-dependent contact intervals.
  - The role of relative orientation in short-range contacts is addressed in Section S1.2. This section also explains how the orientation can be combined with the Euclidean distance to define a metric accounting for residue-residue interactions. The precise form of this combination is determined through empirical analysis of the interactions between amino acids. This is presented in Section S1.3.
  - Then, Section S1.4 defines the contact function for amino acid pairs as a decreasing function of their relative pose distance, whose form is once again empirically calibrated.
  - Section S1.5 presents the dimensionality reduction and clustering algorithms that complete the pipeline.
- In Section S2, we demonstrate that refining the contact definition by removing arbitrary thresholds and incorporating relative orientation significantly improves the performance of the method.
- Section S3 includes supplementary figures complementing the comparative analysis of WARIO with other clustering approaches.
- Section S4 details the results of Section 3.3, that is, the characterization of Huntingtin Exon-1 and TAR DNA-binding protein 43.
- In Section S5, we present the characterization of the N-tail protein from Sendai virus, mentioned in Section 4 of the manuscript.
- Finally, Section S6 presents the complete characterizations of the conformational ensembles for the three proteins studied in this work.

S1 Methods

This section details the main components of WARIO's methodology. To calibrate the *contact function*, we made use of a set of 15177 experimentally-determined high-resolution structures of protein domains extracted from the SCOPe 2.07 release [1]. Throughout this section, this set will be referred to as the *structural database*. Supplementary analyses confirm that the number of structures in the database is sufficient to ensure precise estimates of the conformational preferences of interacting amino acids.

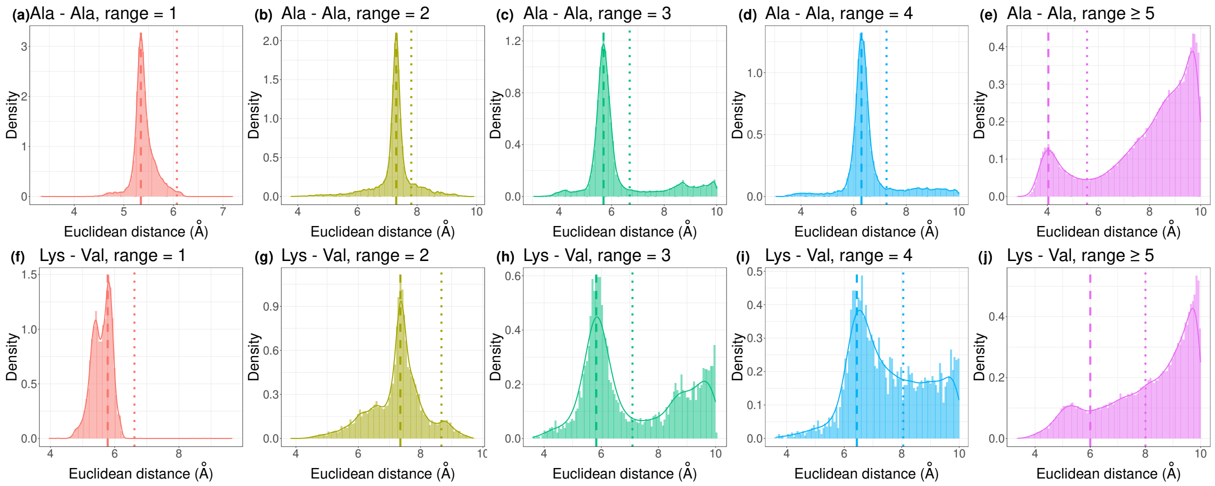

Figure S1: Empirical distribution of the Euclidean distance between (a-e) Ala-Ala and (f-j) Lys-Val residues in the structural database, stratified by range groups. Distributions are depicted through a histogram and a kernel density estimate. Vertical dashed and dotted lines indicate the lower and upper limits of the contact intervals for the Euclidean distance respectively.

S1.1 Contact intervals for the Euclidean distance

Contact between amino acid residues is usually defined by setting universal thresholds to the Euclidean distance between their positions [2]. By universal, we mean that these thresholds are fixed independently of the amino acid identities or their distance along the sequence. However, when looking at how contact distances distribute in nature, we directly observe that residue-residue interactions concentrate around distance values that change according to these parameters. To account for this, we computed the Euclidean distance between every pair of residues in the structural database, and represented their empirical distribution stratifying their identities and range (the distance between both residues along the sequence, in number of residues). Figure S1 presents the encountered distributions truncated to the interval $[0\AA, 10\AA]$ for two pairs of residues at ranges in $\{1, 2, 3, 4\}$ and $[5, \infty)$.

Figure S1 illustrates the fact that the residue-residue Euclidean distance truncated to $[0\AA, 10\AA]$ is not identically distributed across amino acid identities and ranges. Distance values concentrate around sequence-dependent maxima with sequence-dependent variance. Therefore, contact descriptors computed from Euclidean distance should take this information into account and avoid universal thresholds that contradict the empirical behavior. The sequence-specific distance distributions presented in Figure S1 allow us to relax the threshold-based definition of contact for Euclidean distances. Let A_i, A_j denote a pair of amino acid types and $S_{ij} = 1, 2, \ldots$ denote a sequence range. Let $f_{ij}^{\mathbb{R}^3}$ denote the density function of the Euclidean distance distribution for A_i-A_j pairs at range S_{ij} estimated from the structural database and truncated to the interval $[0\AA, 10\AA]$. The *Euclidean contact interval* for A_i-A_j pairs at range S_{ij} is defined as the real interval

$$C_{ij}^{\mathbb{R}^3}(A_i, A_j, S_{ij}) = C_{ij}^{\mathbb{R}^3} = [\Delta_{a;i,j}^{\mathbb{R}^3}, \Delta_{b;i,j}^{\mathbb{R}^3}], \tag{S1}$$

where $\Delta_{a;i,j}^{\mathbb{R}^3}$ is the abscissa smaller than $8\AA$ presenting the highest maximum of $f_{ij}^{\mathbb{R}^3}$ and $\Delta_{b;i,j}^{\mathbb{R}^3}$ is the closest abscissa from the right to $\Delta_{a;i,j}^{\mathbb{R}^3}$ presenting a minimum of $f_{ij}^{\mathbb{R}^3}$. Both limits are depicted in Figure S1 with dashed and dotted lines respectively. For low maximum prominences (as in Figure S1(j)), the Euclidean contact interval is set to $[6\AA, 8\AA]$ by default. Note that, as distance distributions are not markedly different when varying $S_{ij} \geq 5$, we are setting $C_{ij}^{\mathbb{R}^3}(A_i, A_j, S) = C_{ij}^{\mathbb{R}^3}(A_i, A_j, S')$ for every

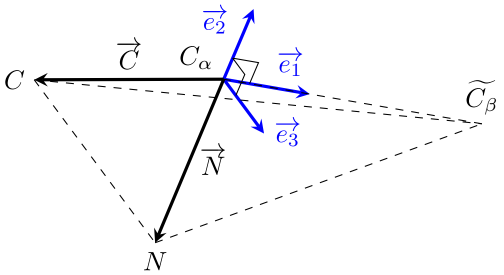

Figure S2: Figure S1 in [8]. The three vectors $\{\overrightarrow{e_1}, \overrightarrow{e_2}, \overrightarrow{e_3}\}$ defining the reference frame, built from the virtual atom $\widetilde{C_\beta}$ and vectors $\overrightarrow{C}$ and $\overrightarrow{N}$.

$S, S' \geq 5$. The complete list of contact intervals and the counterparts of Figure S1 for every amino acid pair and range class are available at https://gitlab.laas.fr/moma/WARIO.

The intervals (S1) allow a continuous description of residue-residue interactions by removing binary contact classifications. The upper limit of (S1) represents the distance value at which the interaction probability starts to be substantial, and continuously increases until reaching the lower limit of (S1), beyond which interaction occurs with high probability. Replacing thresholds by intervals is the key idea to define continuous functions accounting for a contact strength that increases smoothly as the interaction probability starts to be significant. Their explicit applicability in this work is detailed in the following sections.

S1.2 Distance to ideal orientations

Relative orientation plays a determinant role in residue-residue interactions [3–6]. This idea was already incorporated in RING [5, 7], where contact thresholds were defined by integrating the values of backbone angles mediating multiple types of interactions. Here, we propose to capture this effect through a meaningful representation of the spatial pose of each amino acid. This can be achieved by defining a residue-specific reference frame at each C_β atom (C_α for glycines) as it was done in [8]. The detailed construction of the reference system is explained in [8, Section S1.1]. An outline of its definition is presented here. To encode the angular configuration of the backbone at the residue level, we first define a virtual atom $\widetilde{C_\beta}$, which exists also for glycines. The position of $\widetilde{C_\beta}$ is an estimate of the position of the true C_β when it exists, but it is defined for every residue using only the coordinates of the C_α, C and N atoms. We denote as $\overrightarrow{C}$ and $\overrightarrow{N}$ the vectors going from C_α to C and N atoms, respectively, and we define $\overrightarrow{CN} = \overrightarrow{N} - \overrightarrow{C}$. The residue-specific reference frame is defined as follows:

$$\begin{cases} \overrightarrow{e_1} = \overrightarrow{\widetilde{C_\beta}} / \|\overrightarrow{\widetilde{C_\beta}}\| \\ \overrightarrow{e_2} = \overrightarrow{CN} / \|\overrightarrow{CN}\| \times \overrightarrow{e_1} \\ \overrightarrow{e_3} = \overrightarrow{e_1} \times \overrightarrow{e_2}, \end{cases} \tag{S2}$$

where $\times$ denotes the cross-product. An illustration of (S2) is presented in Figure S2. Note that the third basis vector $\overrightarrow{e_3}$ is parallel to $\overrightarrow{CN}$ under the hypothesis that the atoms C, N, C_α and $\widetilde{C_\beta}$ form a perfect tetrahedron. Let L denote the sequence length and $i \in \{1, \ldots, L\}$ the position of the i-th residue. Denoting as $\mathcal{F}_i = \{\overrightarrow{e_{1,i}}, \overrightarrow{e_{2,i}}, \overrightarrow{e_{3,i}}\}$ the reference system (S2) built on the i-th residue, its relative orientation with respect to another residue at position $j \neq i$ will be measured by considering the angles

between the first and third basis vectors:

$$\theta_{1;i,j} = \arccos\langle \overrightarrow{e_{1,i}}, \overrightarrow{e_{1,j}} \rangle, \qquad \theta_{3;i,j} = \arccos\langle \overrightarrow{e_{3,i}}, \overrightarrow{e_{3,j}} \rangle, \tag{S3}$$

where $\langle \cdot\,, \cdot \rangle$ denotes the inner product in $\mathbb{R}^3$. The reason why the angles (S3) were chosen to capture the role of orientation in residue-residue interactions is that they present preferred configurations in nature. This was observed in the structural database for short range contacts i.e. for $S_{ij} = |i - j| < 5$. Figure S3 depicts an example of the empirical distribution of (S3) when the Euclidean distance between both amino acids has crossed the upper limit of the contact interval (S1) i.e. it is smaller than $\Delta^{\mathbb{R}^3}_{b;i,j}$. Indeed, residues beyond the upper limit present preferred relative orientations that are specific to their identities and range. These preferred orientations might not be unique, as in Figure S3(c-d) and represent the contact poses with highest probability in nature. For each pair of amino acid residue types and range class, we took up to three maxima from the density estimates of the empirical distributions of (S3). The maximum with the highest density value was always kept, and the subsequent maxima were kept if their prominence with respect to the first maximum was not negligible. We refer to these maxima as the *ideal orientations* for A_i-A_j pairs at range S_{ij}, and we denote them as

$$\theta^*_{1;i,j} = \theta^*_{1;i,j}(A_i, A_j, S_{ij}) \quad \text{and} \quad \theta^*_{3;i,j} = \theta^*_{3;i,j}(A_i, A_j, S_{ij}) \tag{S4}$$

for the angles between the first and third basis vectors respectively. Note that (S4) are non-empty subsets of $[0°, 180°]$ containing up to three values. The complete list of $\theta^*_{1;i,j}$ and $\theta^*_{3;i,j}$ sets and their corresponding counterparts of Figure S3 are available at https://gitlab.laas.fr/moma/WARIO.

Following the fact that the angles (S3) concentrate around a set of sequence-specific ideal orientations when both amino acid residues interact, it is possible to define how close to the ideal contact setting is the relative orientation of a pair of residues. For two residues at positions $i \neq j$ in the sequence, this is done by considering the *distance between the pair* $\{\mathcal{F}_i, \mathcal{F}_j\}$ *and its ideal orientation*:

$$d^2_{\theta^*}(\{\mathcal{F}_i, \mathcal{F}_j\}) = \frac{1}{4}\, h\left(\min_{\theta \in \theta^*_{1;i,j}} |\theta_{1;i,j} - \theta|\right)^2 + \frac{1}{4}\, h\left(\min_{\theta \in \theta^*_{3;i,j}} |\theta_{3;i,j} - \theta|\right)^2, \tag{S5}$$

where h is a monotonic function on $[0°, 180°]$ defined by $h(x) = \sin(x)$ if $x \leq 90°$ and $h(x) = 1 - \cos(x)$ otherwise. Note that the quantity $d_{\theta^*}(\{\mathcal{F}_i, \mathcal{F}_j\})$ in (S5) takes values in $[0, 1]$, with $d_{\theta^*}(\{\mathcal{F}_i, \mathcal{F}_j\}) = 0$ being a perfect match to the ideal orientation and $d_{\theta^*}(\{\mathcal{F}_i, \mathcal{F}_j\}) = 1$ the strongest disagreement with such setting. Remark also that we have omitted the explicit dependence of (S5) on A_i, A_j and S_{ij} to lighten notation. As we mentioned before, preferred orientations were only found when $S_{ij} = |i - j| < 5$. We refer to this setting as short-range and to the case $S_{ij} \geq 5$ as long-range. Consequently, the relative orientation of the residue pair will only be considered for short-range interactions. In that case, we need to find a suitable strategy to combine distance and orientation information to correctly account for contact. This is addressed in the following section.

S1.3 Relative pose distance

We have sought to define an appropriate combination of Euclidean and orientation distances that correctly acts as proxy for the interaction between residue pairs. Let $i \neq j$ denote two sequence positions and $\mathcal{F}_i$, $\mathcal{F}_j$ the i-th and j-th reference frame defined in (S2). We denote by $d_{\mathbb{R}^3}(\mathcal{F}_i, \mathcal{F}_j)$ the Euclidean distance

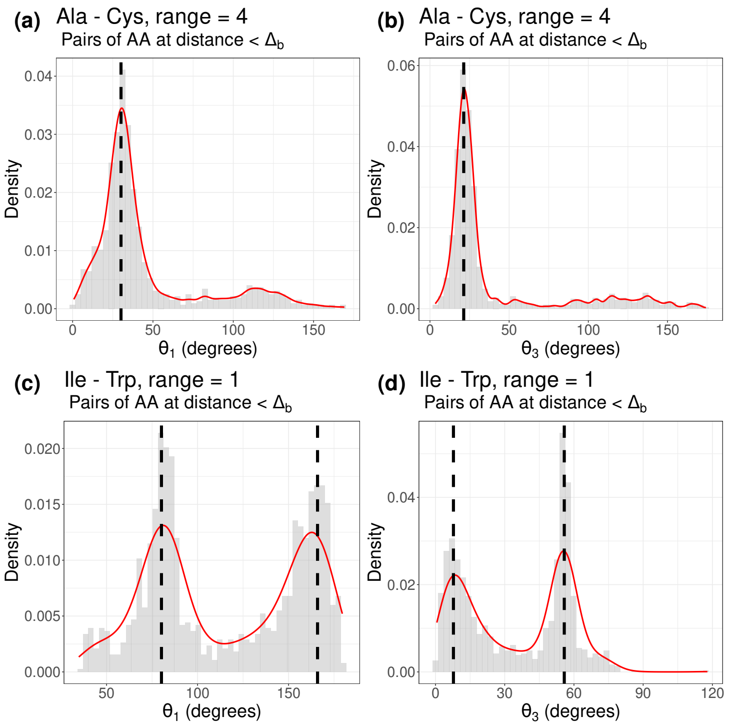

Figure S3: Empirical distribution of angles (S3) computed from the empirical database for pairs of residues at Euclidean distance smaller than $\Delta_b^{\mathbb{R}^3}(A_i, A_j, S_{ij})$, stratified by amino acid types and range. In (a-b), distributions for Ala-Cys pairs at range 4 and, in (c-d), distributions for Ile-Trp pairs at range 1. The non-negligible maxima of the kernel density estimates (red curves) are marked with a dashed black line.

between the positions of both residues. We propose to combine $d_{\mathbb{R}^3}(\mathcal{F}_i, \mathcal{F}_j)$ with (S5) as

$$(1 - \omega_{\theta^*})^2 \, d^2_{\mathbb{R}^3}(\mathcal{F}_i, \mathcal{F}_j) + \omega^2_{\theta^*} \, d^2_{\theta^*}(\{\mathcal{F}_i, \mathcal{F}_j\}), \qquad \text{(S6)}$$

where the weight $\omega_{\theta^*} \in [0, 1]$ governs the distance-orientation balance. Of course, the main problem here is the suitable choice of ω_{θ^*}. This should be done by considering the following guidelines:

(i) relative orientation must only be considered for short-range interactions,

(ii) relative orientation must only be considered when both residues are close in Euclidean distance, i.e. closer than the upper limit of their Euclidean contact interval (S1),

(iii) relative orientation must significantly enhance the contact strength if it is close to the ideal setting, and remain ineffective otherwise.

The first conclusion that can be extracted is that for ω_{θ^*} to satisfy $(i - iii)$ it must be a function of the pair of frames, the amino acid residue types and the sequence range $\omega_{\theta^*} = \omega_{\theta^*}(\mathcal{F}_i, \mathcal{F}_j, A_i, A_j, S_{ij})$. To lighten notation, we will omit the explicit dependence on range and residue identities and write only $\omega_{\theta^*} = \omega_{\theta^*}(\mathcal{F}_i, \mathcal{F}_j)$. The first point ($i$) can be easily guaranteed by asking $\omega_{\theta^*} = 0$ if $S_{ij} \geq 5$. For long-range interactions, contact will be exclusively encoded by the Euclidean distance between both residues. Ensuring (ii) remains to ask ω_{θ^*} to be a decreasing function of $d^2_{\mathbb{R}^3}(\mathcal{F}_i, \mathcal{F}_j)$, whose smooth decay concentrates in the Euclidean contact interval (S1). Finally, satisfying (iii) demands that ω_{θ^*}

is also decreasing with $d_{\theta^*}(\{\mathcal{F}_i, \mathcal{F}_j\})$. Note that the word *significantly* has been added in (*iii*). In other words, ω_{θ^*} needs to balance distance and orientation in a way that they are comparable beyond the Euclidean contact interval when orientation plays a non-negligible role. This can be ensured if the following relation holds:

$$(1-\omega_{\theta^*})^2\, d^2_{\mathbb{R}^3}(\mathcal{F}_i, \mathcal{F}_j) \sim \omega^2_{\theta^*}\, d^2_{\theta^*}(\{\mathcal{F}_i, \mathcal{F}_j\}) \quad \text{for all } d_{\mathbb{R}^3}(\mathcal{F}_i, \mathcal{F}_j) \leq \Delta^{\mathbb{R}^3}_{a;i,j}, \tag{S7}$$

where $\Delta^{\mathbb{R}^3}_{a;i,j}$ is the lower limit of the Euclidean contact interval for A_i-A_j pairs at range S_{ij}, defined in (S1). All these conditions are verified if the following functional form is chosen to define ω_{θ^*}:

$$\omega_{\theta^*}(\mathcal{F}_i, \mathcal{F}_j) = \begin{cases} 1 - \tanh\left[4\left(d^2_{\theta^*}\left(\{\mathcal{F}_i, \mathcal{F}_j\}\right) + g_{ij}\left(d^2_{\mathbb{R}^3}\left(\{\mathcal{F}_i, \mathcal{F}_j\}\right)\right)\right)^2\right] & \text{if} \quad S_{ij} < 5, \\ 0 & \text{otherwise,} \end{cases} \tag{S8}$$

where g_{ij} is a function defined by

$$g_{ij}(x) = \frac{1}{2}\left(\frac{x}{\Delta^{\mathbb{R}^3}_{b;i,j}}\right)^{\frac{d^{\mathbb{R}^3}_{ij}}{2}} \quad \text{for all } x \geq 0 \quad \text{and} \quad d^{\mathbb{R}^3}_{ij} = \frac{\log\left(\operatorname{argtanh}\left(1/\Delta^{\mathbb{R}^3}_{a;i,j}\right)\right)}{\log\left(\operatorname{argtanh}\left(\Delta^{\mathbb{R}^3}_{a;i,j}/\Delta^{\mathbb{R}^3}_{b;i,j}\right)\right)}, \tag{S9}$$

where $\Delta^{\mathbb{R}^3}_{a;i,j}$ (resp. $\Delta^{\mathbb{R}^3}_{b;i,j}$) is the lower (resp. upper) limit of the Euclidean contact interval for A_i-A_j pairs at range S_{ij}, defined in (S1). With this, it is possible to define the *relative pose distance* between the pair of residues A_i-A_j with frames $\mathcal{F}_i$, $\mathcal{F}_j$ at range S_{ij} in the sequence as the function

$$d^2_{\mathrm{RP}}\left(\{\mathcal{F}_i, \mathcal{F}_j\}\right) = \left(1-\omega_{\theta^*}\left(\{\mathcal{F}_i, \mathcal{F}_j\}\right)\right)^2 d^2_{\mathbb{R}^3}(\mathcal{F}_i, \mathcal{F}_j) + \omega^2_{\theta^*}\left(\{\mathcal{F}_i, \mathcal{F}_j\}\right)\, d^2_{\theta^*}\left(\{\mathcal{F}_i, \mathcal{F}_j\}\right), \tag{S10}$$

where we have omitted the dependence on reside types and range for simplicity and $\omega^2_{\theta^*}\left(\{\mathcal{F}_i, \mathcal{F}_j\}\right)$ is defined in (S8). A clear visualization of the orientation weight (S8) and the relative pose distance (S10) is presented in Figure S4 for Ala-Ala pairs at range 3. The curves in Figure S4 show that definition (S8) satisfies conditions $(i - iii)$. Note first that for Euclidean distances greater than the upper limit of the Euclidean contact interval, orientation is not considered to describe interaction. Its contribution smoothly increases when crossing the Euclidean contact interval from right to left, becoming comparable to the one of the Euclidean distance after crossing the lower limit. The rise of ω_{θ^*} is stronger when the relative orientation of $\{\mathcal{F}_i,\, \mathcal{F}_j\}$ gets closer to its ideal setting, and weaker otherwise. Indeed, orientation has no effect in the worst scenario $d_{\theta^*}(\{\mathcal{F}_i, \mathcal{F}_j\}) = 0$. In other words, the role of orientation is to enhance the contact strength defined by the Euclidean distance when it is close to the ideal setting. It is important to remark that the dependence of ω_{θ^*} on Euclidean distance and orientation occurs smoothly in all directions. This is possible thanks to the definition of Euclidean contact intervals (S1), which allows to concentrate the smooth variation of ω_{θ^*} within a sequence-dependent range of values extracted in agreement to the observed empirical behavior.

S1.4 Contact function definition

This section is devoted to define contact between amino acids as a continuous function taking values in $[0, 1]$ and correctly acting as an indicator of their interaction strength. In other words, contact will be defined as a decreasing function of the relative pose distance:

$$\omega^C_{ij}(\{\mathcal{F}_i, \mathcal{F}_j\}) = t_{ij}\left(d_{\mathrm{RP}}(\{\mathcal{F}_i, \mathcal{F}_j\})\right) \quad \text{with } t_{ij} : [0, \infty) \longrightarrow [0, 1] \quad \text{decreasing.} \tag{S11}$$

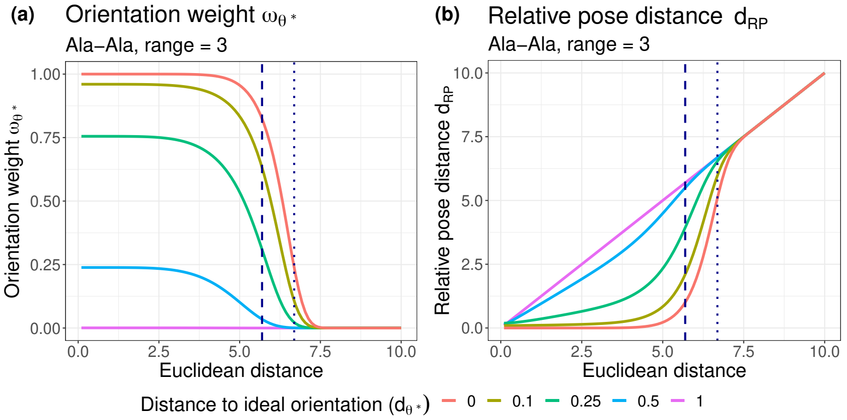

Figure S4: For Ala-Ala pairs at range 3, (a) the weight function (S8) and (b) the relative pose distance (S10). Both quantities are depicted as a function of the Euclidean distance between residue positions and stratified by distance to the ideal orientation. Dashed and dotted vertical lines indicate respectively the lower and upper limit of the Euclidean contact interval.

The contact function ω_{ij}^{C} will take values close to 1 (resp. 0) when the relative pose distance between residues at positions $i \neq j$ is close to (resp. far from) 0. Once again, we will ask the contact function to decrease smoothly with d_{RP} and to concentrate its decay inside an empirically determined interval. To calibrate its functional form, we proceeded analogously to the previous sections, and started by computing the empirical distribution of the relative pose distance (S10) for every pair of amino acids at ranges in $\{1, 2, 3, 4\}$ extracted from the structural database. The results for Ala-Ala and Lys-Val pairs are presented in Figure S5.

Figure S5 illustrates the effect of incorporating (S5) to the Euclidean distance when accounting for short-range residue-residue interactions. If we compare panels in Figure S5 with their counterparts of Figure S1, we see how the relative pose distance (S10) enhances -by translating their Euclidean distance value to the left- those residue pairs whose relative orientation is close to the ideal one. This translation is very clear for pairs at range 1, for which the uni-modal distributions of Figure S1 become bi-modal in Figure S5, but it also appreciable for ranges higher than one, where the probability mass moves to smaller distance values thanks to the residue pairs with low values of (S5). Note that the shift is more visible for contacts at range 1 due to the high concentration of the distance distribution around its mean. Indeed, distances and orientations between consecutive residues are very physically restricted. For longer ranges, the shift is equally present but less appreciable through Figure S5 due to the higher variance of the distance distributions. To conclude, defining (S10) allows us to filter residue-residue interactions that, besides corresponding to amino acids close in Euclidean distance, present ideal relative orientations. We introduce now the analogous contact interval of (S1) for the relative pose distance (S10). Let A_i, A_j denote a pair of amino acid identities and $S_{ij} = 1, 2, \dots$ denote a sequence range. Let f_{ij}^{RP} denote the density function of the relative pose distance distribution for A_i-A_j pairs at range S_{ij} estimated from the structural database and truncated to the interval $[0\AA, 10\AA]$. The *contact interval* for A_i-A_j pairs at range S_{ij} is defined as the real interval

$$C_{ij}^{\mathrm{RP}}(A_i, A_j, S_{ij}) = C_{ij}^{\mathrm{RP}} = [\Delta_{a;i,j}^{\mathrm{RP}}, \Delta_{b;i,j}^{\mathrm{RP}}], \tag{S12}$$

where $\Delta_{a;i,j}^{\mathrm{RP}}$ is the smaller abscissa presenting a maximum of f_{ij}^{RP} and $\Delta_{b;i,j}$ is the closest abscissa from the right to $\Delta_{a;i,j}^{\mathrm{RP}}$ presenting a minimum of f_{ij}^{RP}. As the relative pose distance (S10) corresponds to the

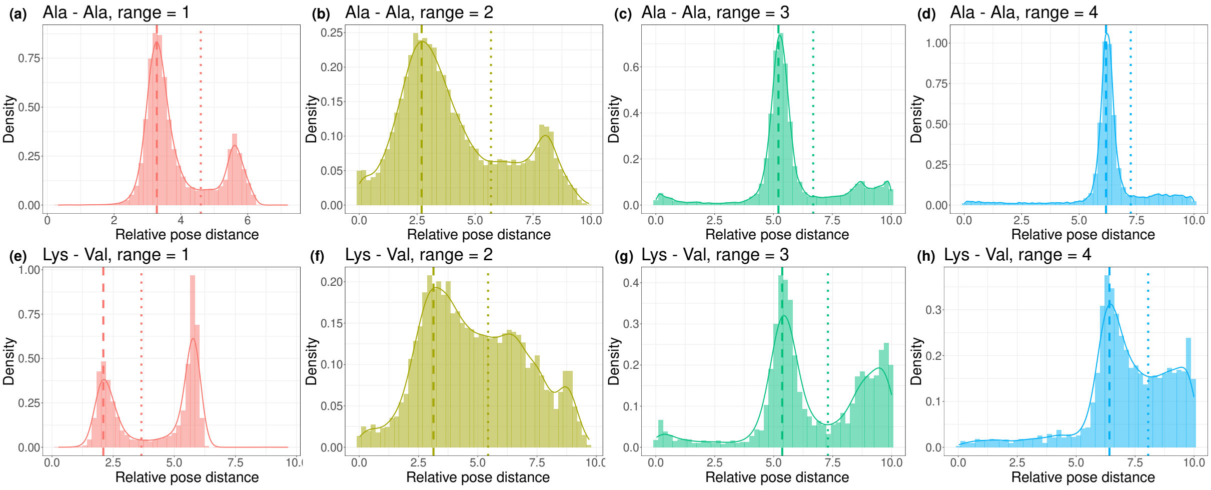

Figure S5: Empirical distribution of the relative pose distance (S10) between (a-e) Ala-Ala and (f-j) Lys-Val residues in the structural database, stratified by range groups. Distributions are depicted through a histogram and a kernel density estimate. Dashed and dotted vertical lines indicate respectively the lower and upper limit of the contact interval.

Euclidean one for $S_{ij} \geq 5$, we have

$$\Delta^{\mathrm{RP}}_{a;i,j} = \Delta^{\mathbb{R}^3}_{a;i,j}, \quad \Delta^{\mathrm{RP}}_{b;i,j} = \Delta^{\mathbb{R}^3}_{b;i,j} \quad \text{for all } S_{ij} \geq 5.$$

Then, we choose the decreasing function t_{ij} in (S11) to concentrate its smooth decay in (S12). This can be done by choosing

$$t_{ij}(x) = 1 - \tanh\left[\left(\frac{x}{\Delta^{\mathrm{RP}}_{b;i,j}}\right)^{d^{\mathrm{RP}}_{ij}}\right] \quad \text{for all } x \geq 0 \quad \text{and} \quad d^{\mathrm{RP}}_{ij} = \frac{\log\left(\operatorname{argtanh}\left(1/\Delta^{\mathrm{RP}}_{a;i,j}\right)\right)}{\log\left(\operatorname{argtanh}\left(\Delta^{\mathrm{RP}}_{a;i,j}/\Delta^{\mathrm{RP}}_{b;i,j}\right)\right)}. \quad \text{(S13)}$$

The curve of t_{ij} is illustrated in Figure S6 for Ala-Ala pairs at range 3. It shows how the contact function (S11) represents a relaxation of the classical step function based on a universal threshold. Here, contact is described by a continuous function whose transition from low to high values is smooth and concentrated inside an empirically determined sequence-specific interval.

S1.5 UMAP and HDBSCAN algorithms

The Uniform Manifold Approximation and Projection (UMAP) algorithm was introduced in the very technical work [9], together with a more accessible and fully detailed documentation [10]. UMAP is a graph layout algorithm incorporating several theoretical foundations that provide it with a robust and well-established framework. Succintly, the UMAP algorithm builds a graph in the high dimensional space and then performs an optimization step to find the most similar graph in a lower dimension. UMAP begins by building balls centered at each point and connecting points whose corresponding balls overlap. This yields the representation of the dataset as a simplicial complex, that captures many of the main topological properties of the high-dimensional space [11]. To deal with the arbitrariness of the radius choice, the connections are made probabilistic and the edges of the graph are weighted. The resulting graphical representation is projected into a lower-dimensional space via a force-directed graph layout algorithm. The optimization procedure is similar to the one of t-SNE [12], but it effectively preserves

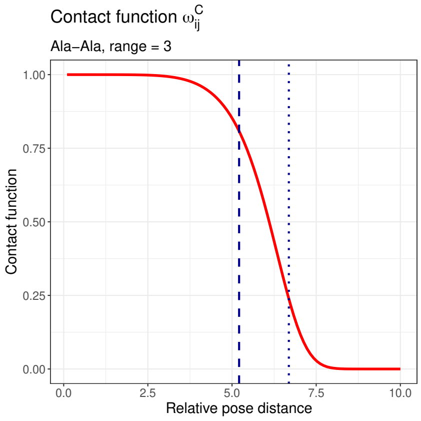

Figure S6: Contact function (S11) as a function of relative pose distance (S10) for Ala-Ala pairs at range 3. Vertical dashed and dotted lines indicate respectively the lower and upper limit of the contact interval.

a more substantial amount of global structure [13]. UMAP has found numerous applications in various domains, such as genetics [14,15], single-cell genomics [16,17] or neuroimaging [18]. Besides, its popularity is steadily increasing due to its demonstrated empirical efficiency, especially in enhancing the performance of clustering methods [19].

HDBSCAN [20] is a hierarchical version of the DBSCAN [21] clustering algorithm. It is a density-based method, so it performs better than classical distance-based techniques like k-means when clusters have arbitrary shapes and sizes, or in the presence of noise or outliers. The algorithm initially follows a similar approach to that of DBSCAN. It involves a density-based transformation of the space, akin to DBSCAN, and subsequently performs single linkage clustering on the transformed space. However, an alternative strategy is carried out to avoid the use of an epsilon value to define a cutoff level for the dendrogram, enabling the identification of the more stable or persistent clusters. Instead of the cutoff parameter, HDBSCAN needs the choice of the minimum cluster size, which is more intuitive and interpretable in practical scenarios. This, together with its remarkable computational efficiency, has made HDBSCAN a very popular algorithm often implemented in combination with dimensionality reduction techniques [15, 22, 23]. For a complete explanation of the algorithm details, we refer to the HDBSCAN documentation [24].

S2 The importance of refining contact definition

We assessed whether the effort made in Section S1 to define contact as a continuous function that integrates sequence and geometrical information is worth it to characterize ensembles. To do so, we kept the same strategy of characterizing an ensemble by a weighted family of contact maps, but starting from the classical contact definition i.e. by considering the matrix

$$\mathbf{C} = \begin{pmatrix} c_{12;1} & \cdots & c_{ij;1} & \cdots & c_{(L-1)L;1} \\ c_{12;2} & \cdots & c_{ij;2} & \cdots & c_{(L-1)L;2} \\ \vdots & & \vdots & & \vdots \\ c_{12;n} & \cdots & c_{ij;n} & \cdots & c_{(L-1)L;n} \end{pmatrix}, \tag{S14}$$

with $c_{ij,k} = 1$ if $d_{\mathbb{R}^3}(\mathcal{F}_i^k, \mathcal{F}_j^k) \leq 8\mathring{A}$ and $c_{ij,k} = 0$ otherwise, where $\mathcal{F}_i^k$ denotes the i-th reference frame (S2) for the k-th conformation. As the entries of (S14) are binary, we chose the Jaccard distance to project the data into the 10-dimensional UMAP space. Then, the clustering was performed using the Euclidean distance between points in the low-dimensional space. Of course, using the classical contact definition based on thresholds imposes the need of metrics that are well-defined for this type of data. The choice of such metric is not straightforward and neither is its suitability in the low-dimensional projection. Whether we can correctly compare points in the UMAP space with the Euclidean distance when the high-dimensional space is $\{0, 1\}^p$ is not a trivial question to address. Moving to the continuous scenario might ease the interpretation of the low-dimensional projection when using exclusively the Euclidean distance between points.

After implementing the previously described analysis to the CHCHD4 ensemble, we observed a substantial disagreement between methods when looking at the number of classified conformations. When using (1), WARIO classified the 78% of conformations. This proportion decreased to 65% when using (S14). The number of retrieved clusters was almost the same as in Section 3.1, where WARIO found 23 classes versus the 22 retrieved here, using the same value for the minimum cluster size: the 1% of n. When looking at the cluster-specific contact maps, we find similar contact trends when comparing both approaches. We can identify groups of conformations similarly classified with both approaches by detecting visually matching contact maps. Three examples are presented in Figure S7. This was expected as the definition proposed in Section S1 is a refinement of the classical one, and no extreme disagreements should appear.

However, remarkable differences appear when diving into short-range contacts, for which relative orientation played a role in the relative pose distance (S10). To illustrate this, we focus on the last row of Figure S7. Both contact maps seem to indicate the presence of helical motifs near the C-terminal. We already showed it in Figure 3(e) for the continuous contact definition. Conformations belonging to the corresponding cluster exhibit α-helix structure at residues 21-24, which is confirmed by the DSSP propensities presented in Figure S8(a). However, despite the visual similarity of panels (e) and (f) in Figure S7, we can appreciate that values for the continuous contact function (panel (e)) are slightly higher at the C-terminal than the ones for the binary definition (panel (f)). This means that residues 21-24 are *closer in relative pose distance* (S10) *than in Euclidean distance.* In other words, taking relative orientation into account enhances contact identification when it is close to the preferred behavior observed in nature. Indeed, the proportion of α-helix structures at 21-24 in cluster 6 for the binary contact clustering (see Figure S8(b)) is considerably smaller. This can be alternatively illustrated by looking at the conformations from such cluster, shown in Figure S8(c), which differ from the structured behavior depicted in Figure 4(e). Consequently, redefining contact as a continuous function (S11) that integrates sequence information and relative orientation is crucial to make the classification coherent in terms of secondary structure.

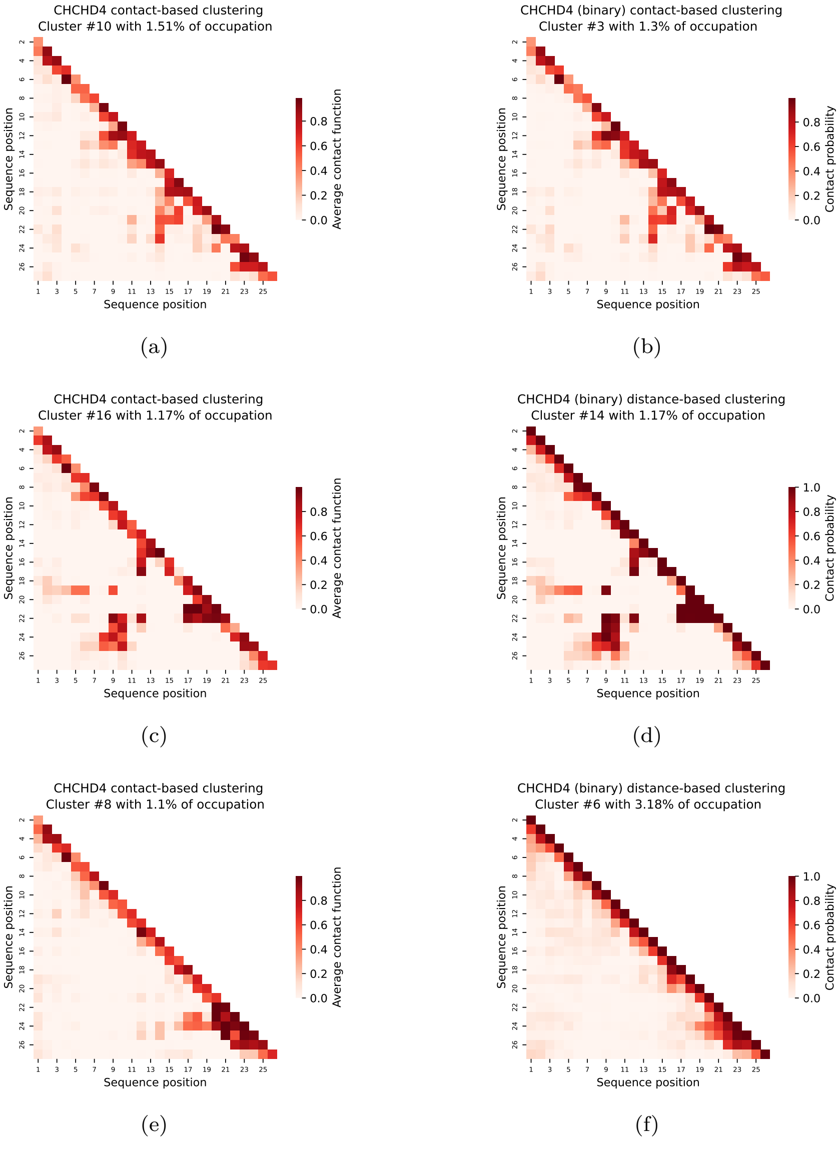

Figure S7: Left (resp. right) column: cluster-specific ω-contact maps (resp. average contact maps) for CHCHD4 after performing the UMAP+clustering pipeline on (1) (resp. (S14)). Maps in the same row are those who visually match each other among both classification techniques.

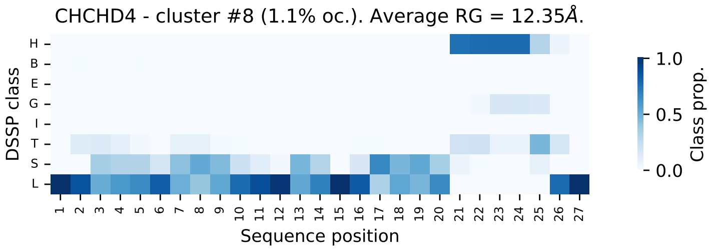

(a) UMAP+HDBSCAN on (1).

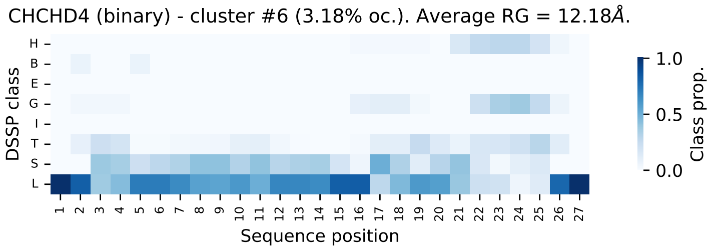

(b) UMAP+HDBSCAN on (S14).

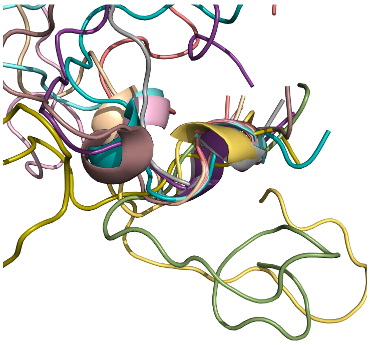

(c) Random conformers of cluster 6.

Figure S8: (a-c): Average DSSP secondary structure propensities across cluster conformations after performing the UMAP+HDBSCAN pipeline on (1), for cluster 8 (a) and on (S14), for cluster 6 (b), for the CHCHD4 ensemble. (c): 10 conformations randomly selected from cluster 6 in the previously detailed conditions.

S3 Additional analyses using other clustering approaches

S3.1 Supplementary figures

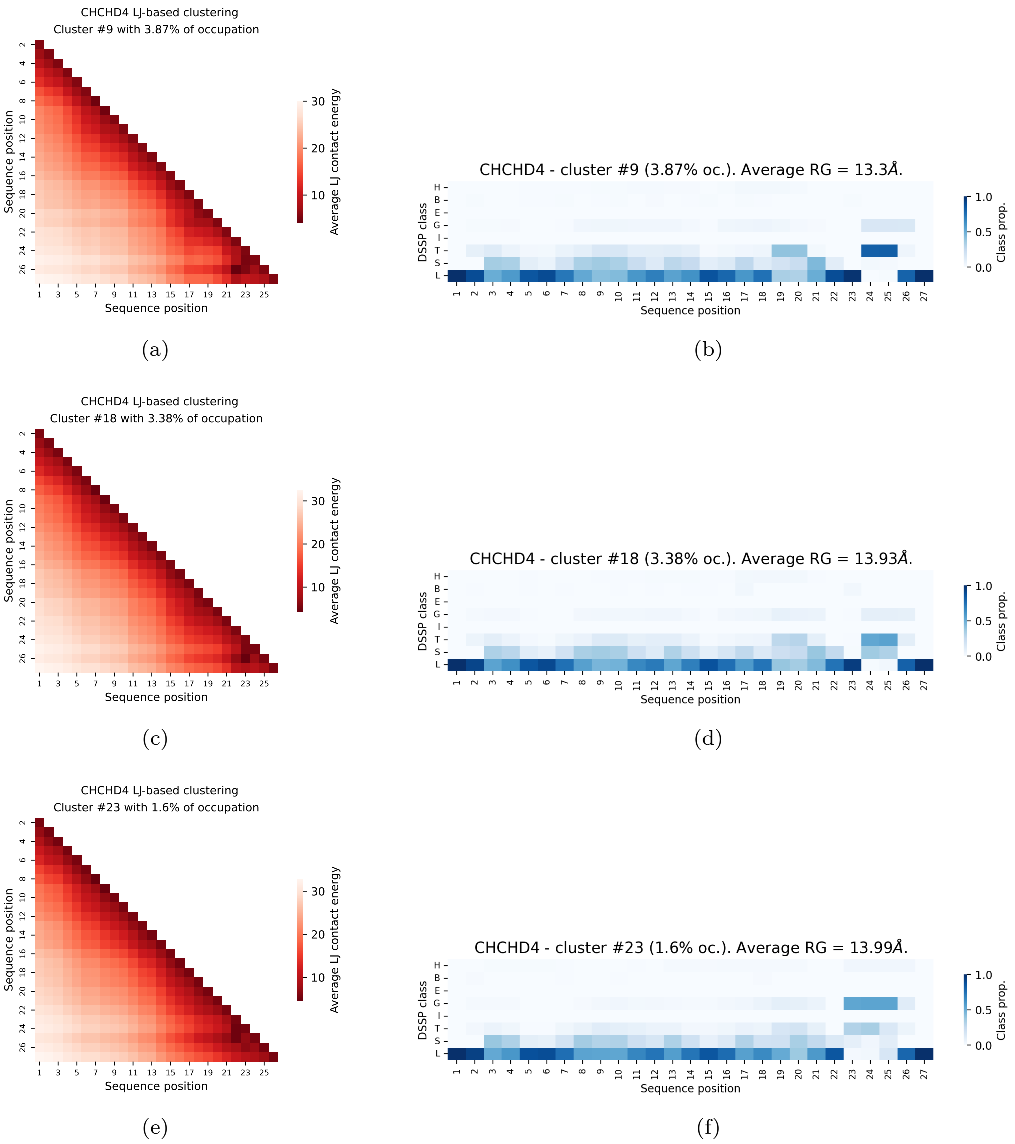

Figure S9: Left column: CHCHD4 cluster-specific Lennard-Jones contact maps after implementing the UMAP+clustering pipeline to the set of all inter-residue LJ potentials. Right column: average DSSP secondary structure propensities across cluster conformations corresponding to the cluster in the same row, left column.

S3.2 Global metric between clustering approaches

We compared the presented clustering approaches using the Adjusted Rand Index [25]. Intuitively, this score represents the probability that a pair of clustering algorithms will agree on classifying (in the same or different groups) a randomly chosen pair of observations. The Rand index takes values in $[0, 1]$, with

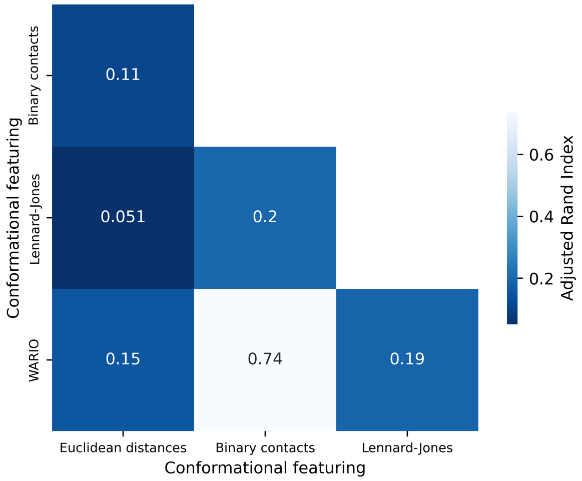

Figure S10: Adjusted Rand Index comparing WARIO and the three alternative featurizations mentioned in the manuscript.

0 corresponding to clusterings that do not agree on any pair of points and 1 indicating that the data clusterings are exactly the same. Here, we have compared the partitions of the CHCHD4 ensemble that were retrieved by WARIO and the three alternative approaches mentioned in the manuscript, that is, the UMAP+HDBSCAN pipeline applied on conformations featured with (*i*) inter-residue Euclidean distances, (*ii*) Lennard-Jones interaction potentials and (*iii*) the classical binary contacts. To filter out the noise, we kept conformations that were classified by HDBSCAN in all cases. Results, presented in Figure S10, show that the clasical contact featurization yields the closest partition to WARIO's, as it was expected. Largest differences appear between WARIO and the remaining featurizations, especially with the Euclidean distance based approach. In any case, note that a global score has to be dealt with care when used to compare two clustering algorithms, as it might be very sensitive to hyper-parameters like the number of clusters. Local comparisons between families of contact maps are more reliable and informative in this context.

S4 Results of Section 3.3

S4.1 Characterization of Huntingtin Exon-1 MD trajectory

The N-terminal region of huntingtin, the so-called exon-1, is the causative agent of Huntington's disease, a deadly neurodegenerative pathology [26]. This fragment contains a poly-glutamine tract, poly-Q, that is flanked by 17 amino acids (N17) and a proline-rich region at N- and C-termini, respectively. Importantly, when the length of the poly-Q exceeds a pathological threshold of 35, the protein forms large amyloidogenic aggregates in neurons that cause the pathology [27]. The structural changes occurring to the protein above this threshold have been the object of huge research efforts from experimental [28, 29] and theoretical perspectives [30]. In a recent study, huntingtin exon-1 (HTTExon-1) was described as an equilibrium of multiple partially helical states involving the N17 and increasing fragments of the poly-Q tract [31]. A MD simulation of a HTTExon-1 encompassing the N17, a poly-Q tract with 46 glutamines and five prolines was performed with the aim to decipher the mechanisms governing the structure of the

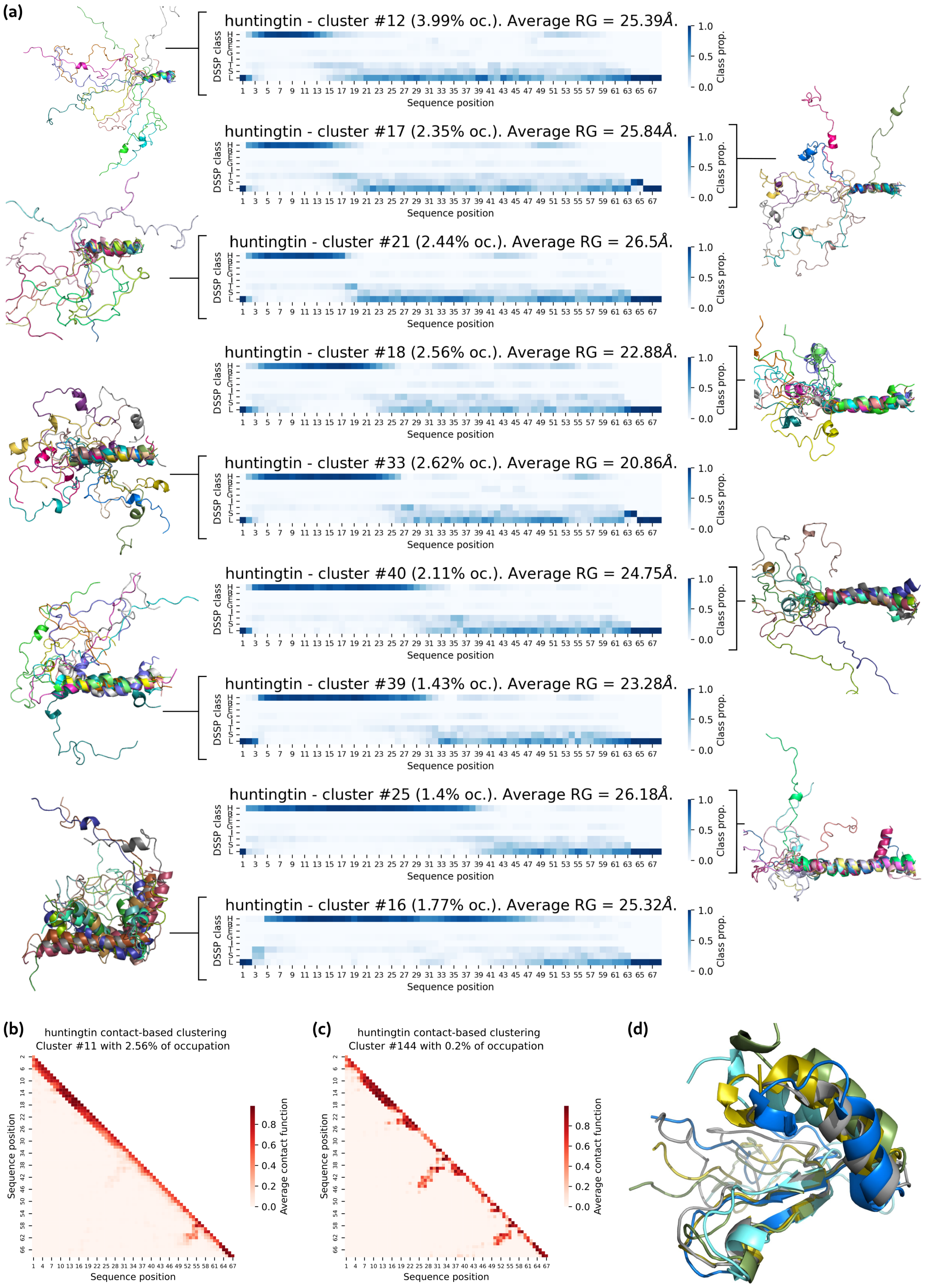

Figure S11: Structural analyses of HTTExon-1 MD trajectory. (a) Nine HTTExon-1 clusters represented by their DSSP secondary structure propensities and ordered by increasing number of residues involved in the α-helix. Together with each DSSP matrix, ten conformations randomly selected from the group and aligned on the α-helix are depicted. (b, resp. c) Cluster-specific ω-contact map for the 11-th (resp. 144-th) cluster of HTTExon-1 characterization after setting to the 1% (resp. 0.1%) of n as the minimum cluster size. (d) Ten conformations randomly selected from the 144-th cluster and aligned on the extended sheet structure around residue 35. Note that the cluster numbering is arbitrary and it is not related with its population.

protein. Details of this simulation can be found in the original publication [31]. Here, we have analyzed this 20 microsecond trajectory ($n = 96000$) with WARIO in order to disentangle the conformational states of this protein.

WARIO found a large number of low-populated clusters, $K = 43$, in the ensemble of HTTExon-1. Interestingly, the 43 clusters presented comparable sizes, grouping 1-3% of the conformations when defining to 1% of n the minimum cluster size. The overall distribution of the weights (3) is illustrated by projecting (1) to a two-dimensional UMAP space (see Section S6). Our approach identified a family of structural clusters displaying a systematic helix extension along the poly-Q tract. Among the 43 clusters, very few long-range contacts emerged from the analysis, the clustering being mainly governed by the short-range helical motifs, which appear close to the diagonal. Indeed, these clusters can be structurally interpreted by monitoring the number of residues involved in the α-helix, which naturally emerge from the cluster-specific ω-contact maps and their DSSP secondary structure analysis. This is illustrated for a group of representative clusters in Figure S11(a) (see Section S6 for the complete characterization). Note that the detection of the different steps along the secondary structure formation is not hampered by the rest of the chain, which preserves an important level of disorder, underlining the power of WARIO to focus on the relevant inter-molecular interactions governing the structure.

We also used the example of HTTExon-1 to assess the effect of the clustering resolution, which can be adjusted by selecting the minimum cluster size, for the detection of extremely low populated states in ensembles. Note that, in some cases, the detection of structural motifs or contact patterns could be essential, but they may go unnoticed if the resolution of the analysis is not sufficiently high. For instance, the cluster-specific ω-contact map for the 11-th cluster, presented in Figure S11(b) and corresponding to the 2.56% of conformations, displays a network of long-range interactions between residues ~50-55 and ~60-65, and a much less intense contact pattern around residue 35, which could correspond to a β-sheet. This last feature is barely appreciated as its corresponding contact function average value remains around 0.1-0.2. Moreover, it is unclear whether the two structural motifs can simultaneously occur in the trajectory. This can be confirmed by refining the clustering at higher resolution and evaluating the partitioning of the 11-th cluster conformations in the new analysis. Using a minimum cluster size of 0.1% of the total number of conformations, WARIO retrieved 440 clusters. One of them, containing 0.2% of the conformations, clearly captured the previously alluded β-sheet contact pattern (Figure S11(c,d)). Importantly, this cluster was the only one presenting this structural pattern, which appeared simultaneously to the C-terminal long-range interaction. These results show that WARIO is able to detect and group the 192 conformations presenting this particular contact pattern among the 96000 of the ensemble, highlighting its sensitivity to scarcely populated intramolecular contacts.

S4.2 Characterization of TAR DNA-binding protein 43 (TDP-43)

TAR DNA-binding protein 43 (TDP-43) is a multidomain protein involved in key RNA regulatory processes and it is commonly associated with various neurodegenerative disorders, including amyotrophic lateral sclerosis and frontotemporal dementia [32]. Multiple investigations suggest that TDP-43 is capable of experiencing liquid-liquid phase separation (LLPS) in vitro and to integrate into biological condensates [33]. The domain architecture of TDP-43 consists of a folded N-terminal domain (NTD, res. 1–78), two RNA-recognition motif domains (RRM1/2, res. 104–263) connected by a short linker, and a disordered C-terminal region (CTD, res. 264–414) that contains an evolutionary conserved region (CR, res. 319–341) known to populate a transient α-helical structure that is key for droplet formation [34]. The role of the different protein domains in the intra- and intermolecular interactions that give rise to LLPS is not

yet fully understood [35]. In this context, multi-scale simulations are a suitable approach to rationalize experimental results [36, 37].

In a recent study, all-atom MD simulations of TDP-43 (414 residues) at two different ionic strengths (100 and 300 mM NaCl) were analyzed to unravel the interdomain interactions governing phase separation [37]. Through an average contact map, in the original study, a complex interaction network involving linkers and the disordered C-terminal domain was identified. Here, we applied WARIO to the ensembles generated by both all-atom MD simulations, showing that the method is capable of finely disentangling the interdomain interactions that occur across the system. For each NaCl concentration, the trajectory was formed by the concatenation of three independent replicas of 5000 conformations. Details on the MD simulations can be found in [37]. Average distance representations used in the original study, while capable of detecting the most prevalent contacts, loose information about the correlation between different interactions, hampering the identification of these occurring simultaneously. The WARIO analysis of the low ionic strength trajectory is used here to exemplify the interaction network patterns. Two cluster-specific contact maps are presented to illustrate this point (Figure S12). The complete characterization at 100 mM NaCl is included in Section S6. The first one shows a simultaneous interaction of the folded N-terminal domain NTD with both C-terminal disordered regions (IDR1, res. 263-318; IDR2, res. 342-414), as well as an intradomain interaction between IDR1 and IDR2 (Figure S12(a)). In the second one, IDR2 interacts simultaneously with NTD, IDR1, the first linker (L1, res. 79-103) and the conserved region (CR, res. 319-341) of the C-terminal domain (Figure S12(b)). Interestingly, this last configuration induces the formation of an anti-parallel extended sheet in IDR2 involving residues 342-343 and 373-374 (Figure S12(c)). By inspecting all clusters derived from the trajectory at 100 mm NaCl, this motif only appears when the above-described long range interaction pattern of the 27th cluster is present. These concomitant interactions could not be identified in the original analyses.

The accurate clustering of conformations provided by WARIO enables the comparison between ensembles obtained with different parameters to identify the structural features that change between them. To exemplify this point, we have compared the clusters derived from the trajectories obtained at both ionic strengths. First, we focus on an interaction that remained unaltered with the salt concentration according to the original study: the long-range contact between the first linker L1 and the second RNA-recognition motif domain RRM2 (Figure S12(d)). At 100 mM NaCl, this interaction was clearly observed within 9 clusters containing 16.32% of conformations. Besides, L1-RRM2 contacts were coupled with other inter-domain interactions. Concretely, clusters 17 (lower triangle of Figure S12(d)) and 19 (comprising 3.99% of conformations altogether) presented simultaneous NTD-IDR1, NTD-IDR2, IDR1-IDR2, RRM1-IDR1 and RRM1-L2 interactions. Clusters 39, 40 and 43 with 5.25% of conformations presented L2-RRM1 interactions (see Section S6 for the complete family of cluster-specific ω-contact maps at both ionic strengths). However, the L1-RRM2 interaction at 300 mM NaCl was uncoupled to any other long-range contact, appearing as the only inter-domain interaction within the corresponding clusters (see e.g. the upper triangle in panel (e) of Figure S12). This observation suggests that the ionic strength selectively breaks some intramolecular interactions, while keeping others unaltered.

Interestingly, in the original study, slight perturbations in the interaction network involving disordered regions and linkers were detected for TDP-43 when the salt concentration was increased [37]. For instance, the interaction between IDR1 and IDR2 was reported to disappear at high ionic strength. The inspection of the contact maps of the 300 mM trajectory clusters, indicates that this contact is observed for some conformations, although its population diminishes (from 27.25% at 100 mM to 15.01% at 300 mM), underlining the higher sensitivity of our approach with respect to averaged contact maps (Figure S12(e)). This feature of WARIO is also evidenced for the contact between residues 353-355 of IDR1 and 285-287

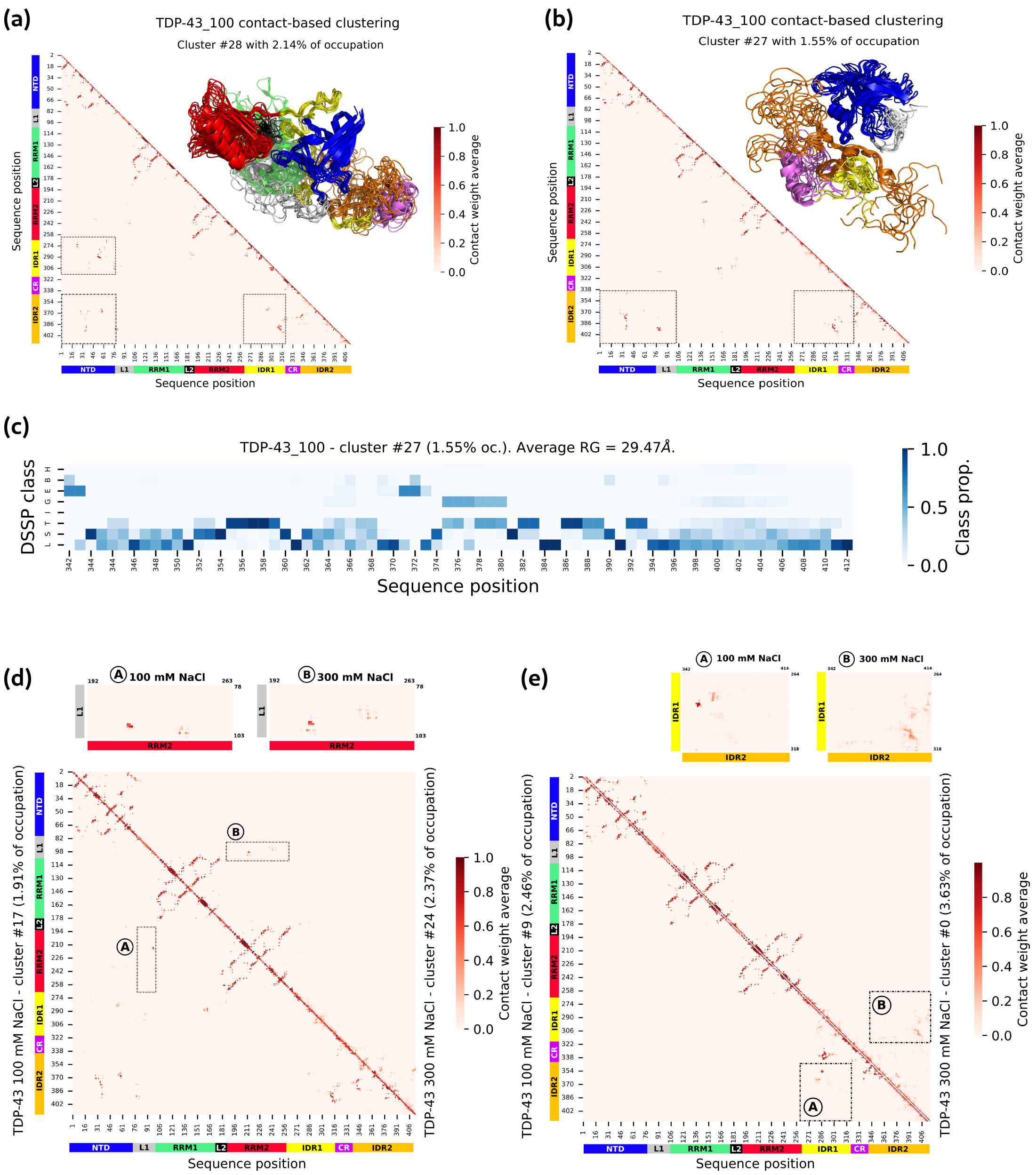

Figure S12: (a,b) Cluster-specific ω-contact maps for two clusters of the TDP-43 ensemble at 100 mM NaCl. In the upper triangles, 10 conformations randomly extracted from the corresponding cluster, aligned on residues that present prominent interactions. The N-terminal domain (NTD) is colored in blue, the two RNA recognition motif-containing domains (RRM1, RRM2) are colored in green and red respectively, the first and second linkers (L1, L2) are colored in gray and black respectively, the two intrinsically disordered regions (IDR1, IDR2) are colored in yellow and orange respectively and the conserved region (CR) is colored in violet. In (b), RRM1, RRM2 and L2 have been removed for easier visualization. (c) Average DSSP secondary structure propensities and average radius of gyration across the 27th cluster conformations, restricted to the IDR2 residues. (d,e) Two pairs of cluster-specific ω-contact maps comparing clusters presenting RRM1-L1 (d) or IDR1-IDR2 (e) inter-domain interactions, at both salt concentrations (100 mM lower triangle, 300 mM upper triangle).

of IDR2 identified with WARIO (see e.g. cluster 9 in the lower triangle of Figure S12(e)), which was not detected in the original study.

S5 Characterization of Sendai virus N-tail

We applied WARIO to analyze two conformational ensembles of the Sendai virus N-tail [38]. The ensembles were generated by a statistical sampling method described in [39] and using two different approaches. The first used three-residue-based sampling (TRS) in the fragment V476-E489, which correctly samples conformations with helical propensity in this region of the protein (see [39] for details). The second employed single-residue-based sampling (SRS), which builds random coil models. The WARIO analysis of the TRS ensemble classified the 99.95% of conformations and identified three clusters encompassing helical conformations of different length and position. These clusters represent 59.12% of the conformations. Results are presented in Figure S13. Not surprisingly, no structurally meaningful clusters could be identified by WARIO when doing the same analysis to the SRS ensemble.

S6 Additional results

This Section presents the complete characterizations of the conformational ensembles for the three proteins studied in this work. For each one of the examples, we first present the two-dimensional UMAP embedding of conformations featured by contact functions. Points are colored according to the HDB-SCAN classification, illustrating the overall distribution of the cluster occupancies. Then, the complete family of weighted ω-contact maps is presented for the ensemble. Finally, we show the secondary structure propensities for the conformations of each cluster, together with their average radius of gyration. For each one of the latter plots, DSSP classes are depicted in decreasing ordinates as: H (alpha helix), B (residue in isolated beta-bridge), E (extended strand, participates in beta ladder), G (3-helix (3/10 helix)), I (5-helix (pi helix)), T (hydrogen bonded turn), S (bend) and L (loops and irregular elements).

S6.1 Complete characterization of CHCHD4

S6.1.1 Two-dimensional UMAP projection

S6.1.2 Complete family of weighted ω-contact maps

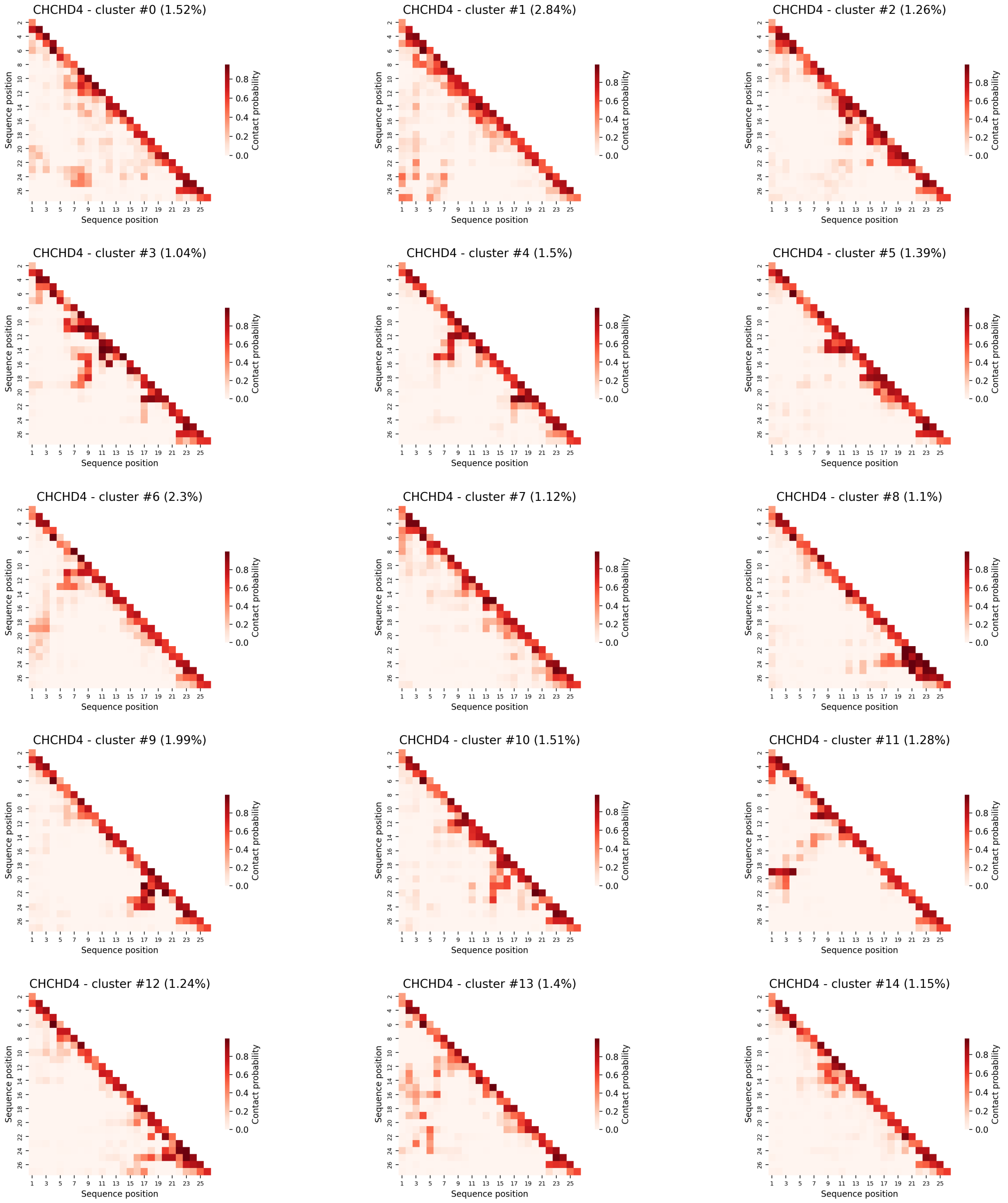

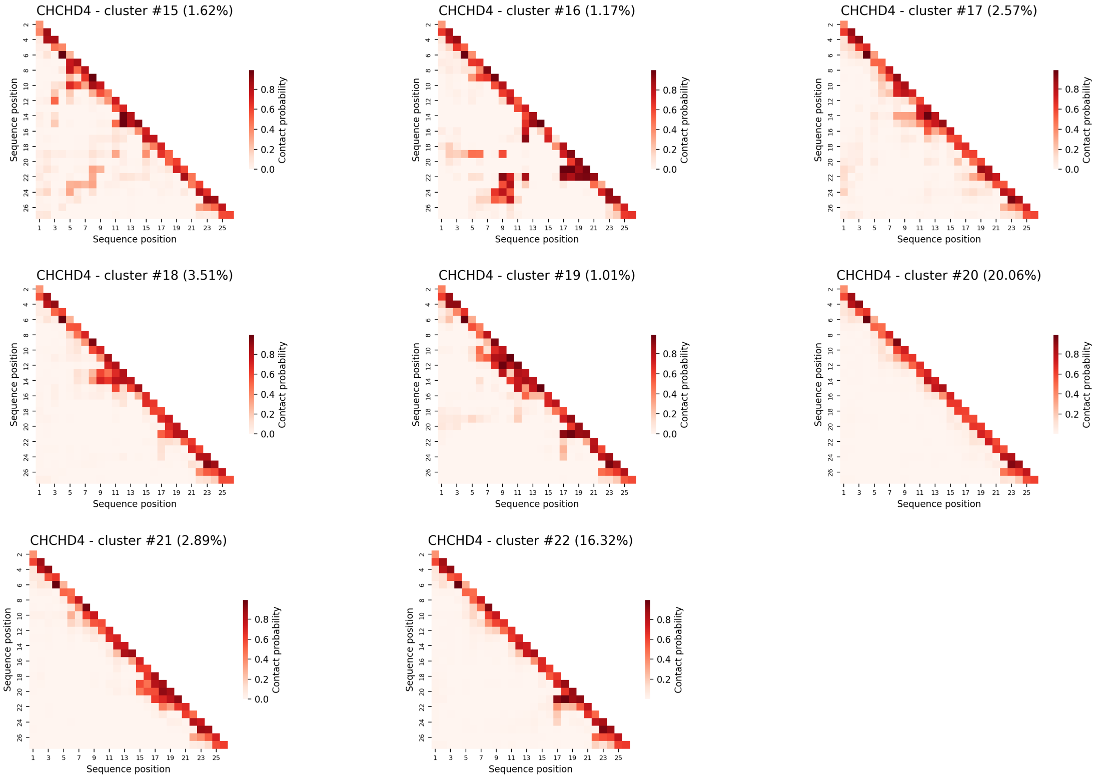

S6.1.3 Secondary structure propensities and average radii of gyration

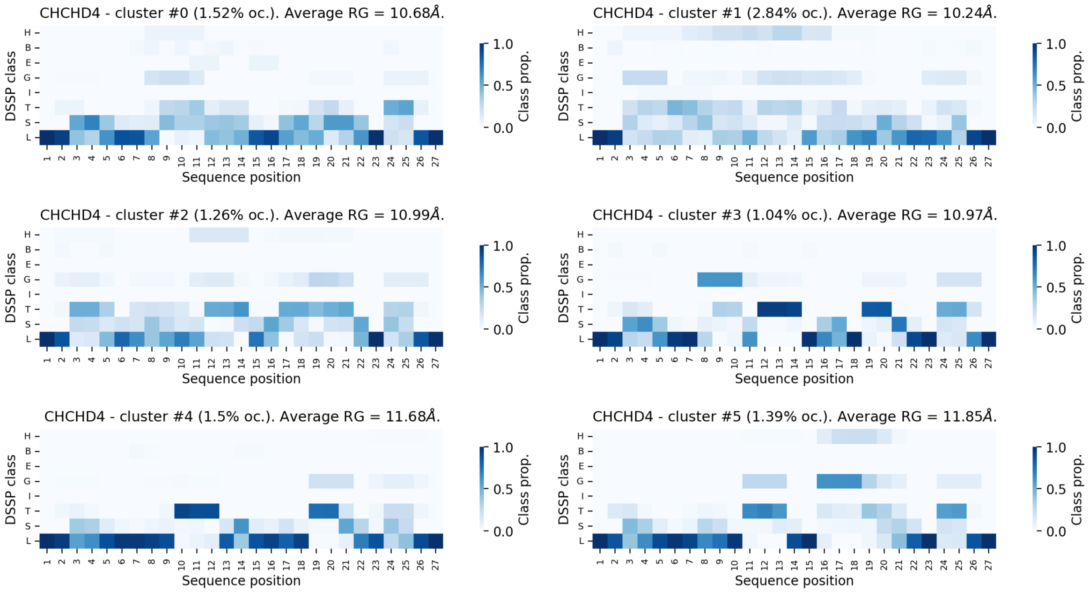

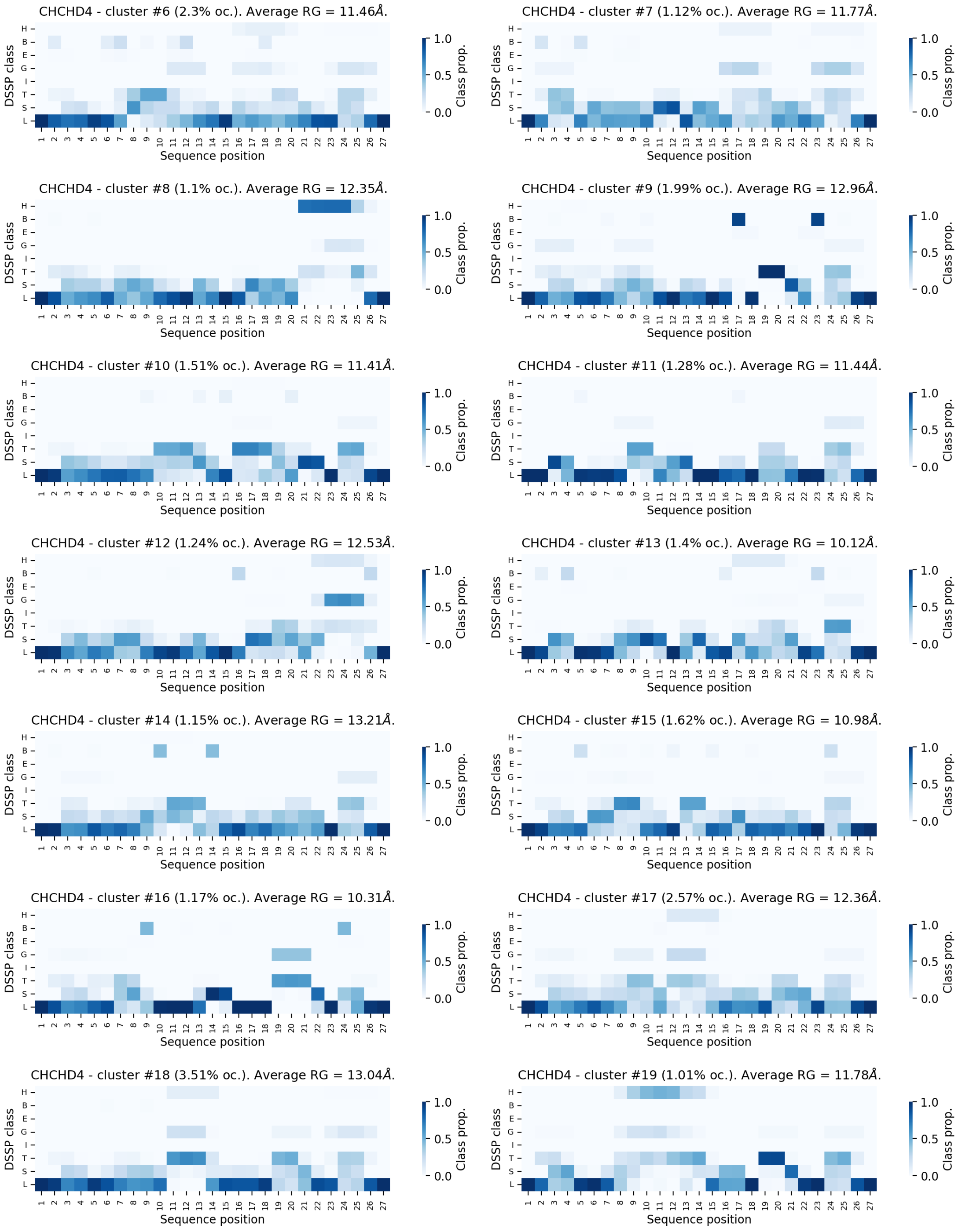
CHCHD4 - cluster #6 (2.3% oc.). Average RG = 11.46Å.
CHCHD4 - cluster #7 (1.12% oc.). Average RG = 11.77Å.
CHCHD4 - cluster #8 (1.1% oc.). Average RG = 12.35Å.
CHCHD4 - cluster #9 (1.99% oc.). Average RG = 12.96Å.
CHCHD4 - cluster #10 (1.51% oc.). Average RG = 11.41Å.
CHCHD4 - cluster #11 (1.28% oc.). Average RG = 11.44Å.
CHCHD4 - cluster #12 (1.24% oc.). Average RG = 12.53Å.
CHCHD4 - cluster #13 (1.4% oc.). Average RG = 10.12Å.
CHCHD4 - cluster #14 (1.15% oc.). Average RG = 13.21Å.
CHCHD4 - cluster #15 (1.62% oc.). Average RG = 10.98Å.
CHCHD4 - cluster #16 (1.17% oc.). Average RG = 10.31Å.
CHCHD4 - cluster #17 (2.57% oc.). Average RG = 12.36Å.
CHCHD4 - cluster #18 (3.51% oc.). Average RG = 13.04Å.
CHCHD4 - cluster #19 (1.01% oc.). Average RG = 11.78Å.
DSSP class
Sequence position
Class prop.

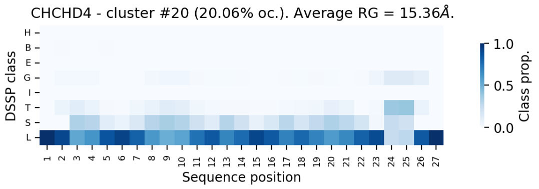

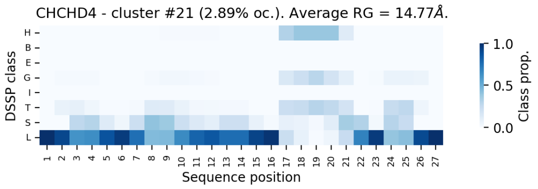

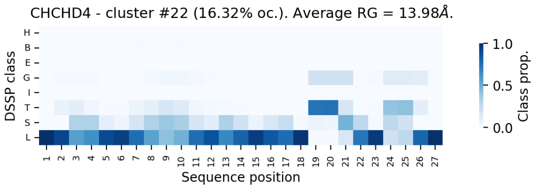

S6.2 Complete characterization of Huntingtin

S6.2.1 Two-dimensional UMAP projection

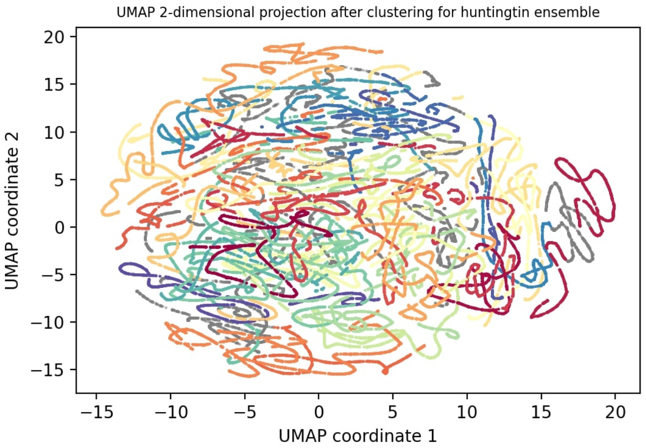

S6.2.2 *Complete family of weighted ω-contact maps*

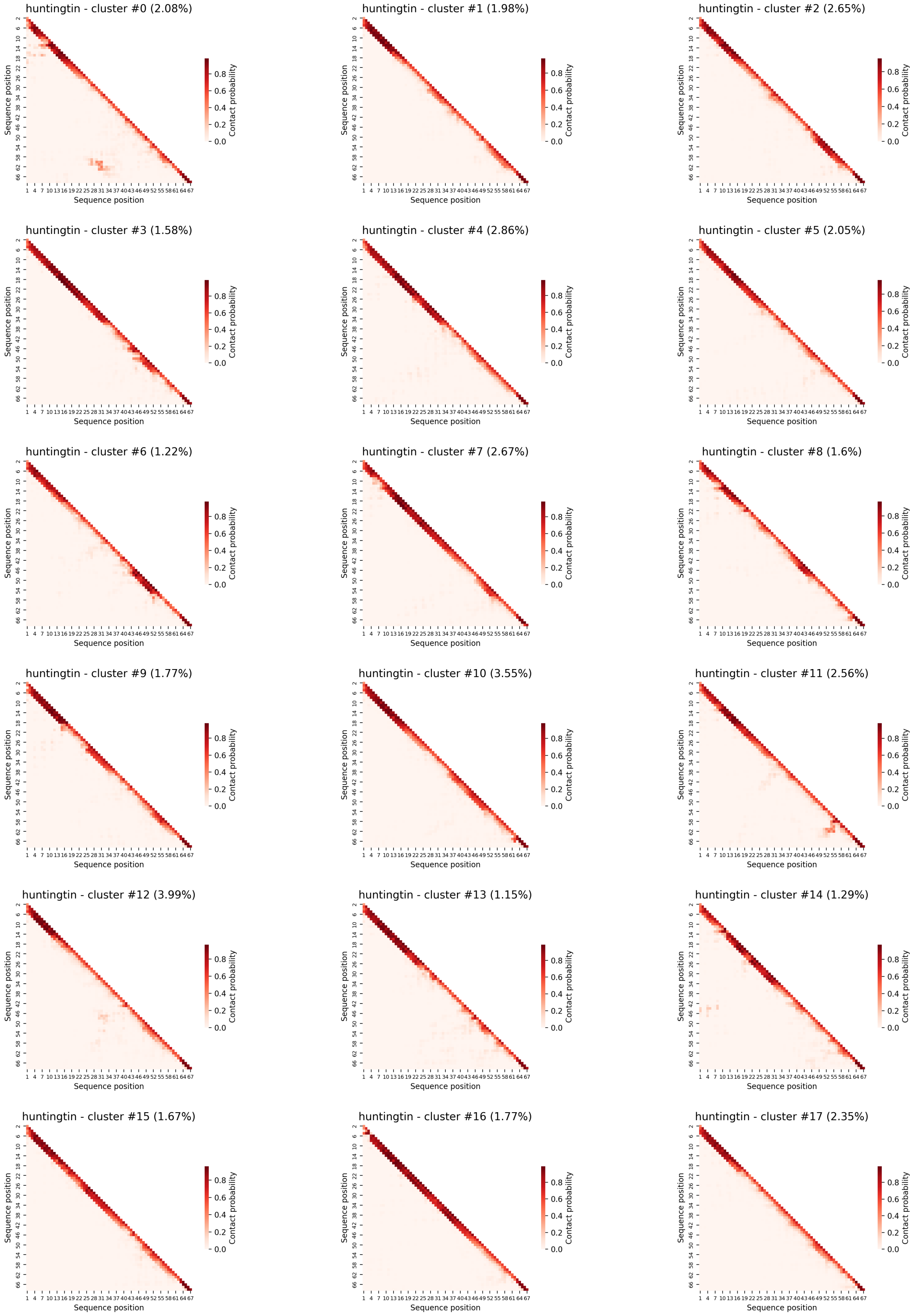

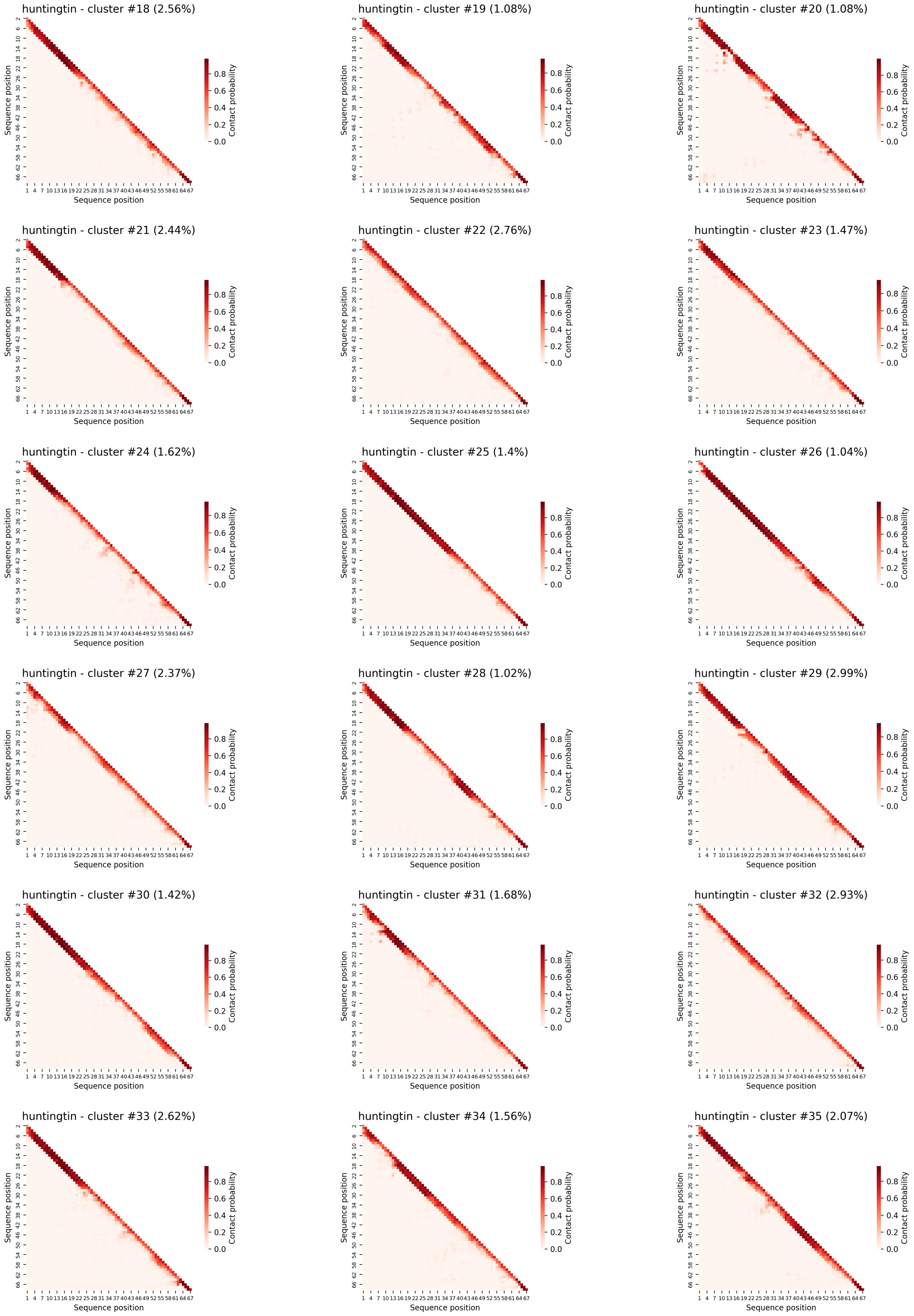
huntingtin - cluster #18 (2.56%)
huntingtin - cluster #19 (1.08%)
huntingtin - cluster #20 (1.08%)
huntingtin - cluster #21 (2.44%)
huntingtin - cluster #22 (2.76%)
huntingtin - cluster #23 (1.47%)
huntingtin - cluster #24 (1.62%)
huntingtin - cluster #25 (1.4%)
huntingtin - cluster #26 (1.04%)
huntingtin - cluster #27 (2.37%)
huntingtin - cluster #28 (1.02%)
huntingtin - cluster #29 (2.99%)
huntingtin - cluster #30 (1.42%)
huntingtin - cluster #31 (1.68%)
huntingtin - cluster #32 (2.93%)
huntingtin - cluster #33 (2.62%)
huntingtin - cluster #34 (1.56%)
huntingtin - cluster #35 (2.07%)
Sequence position
Contact probability

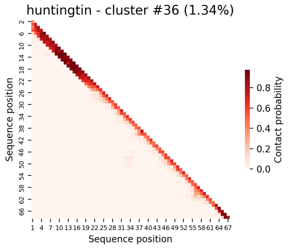

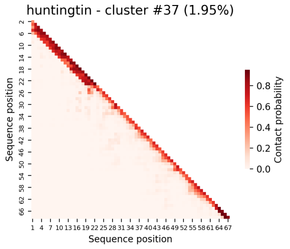

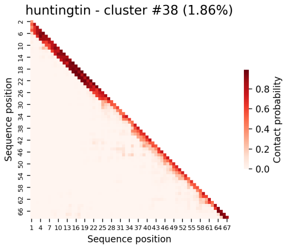

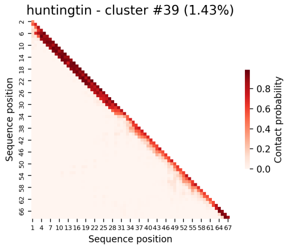

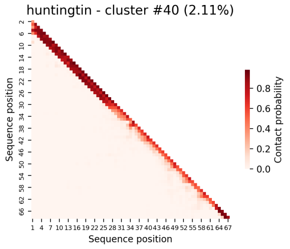

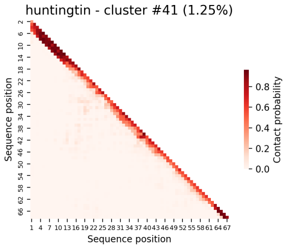

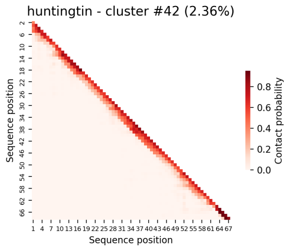

S6.2.3 Secondary structure propensities and average radii of gyration

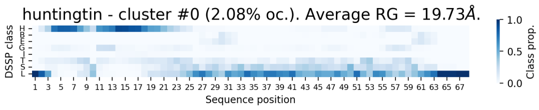

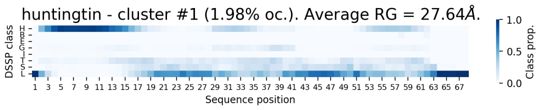

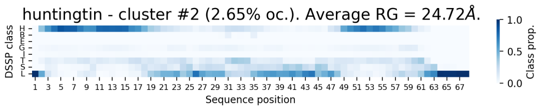

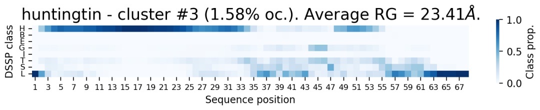

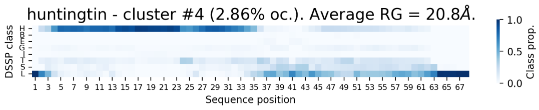

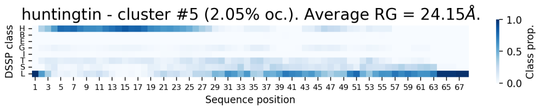

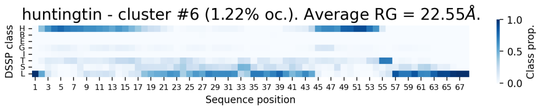

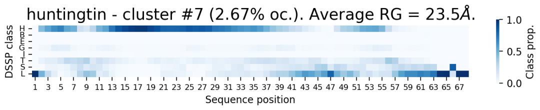

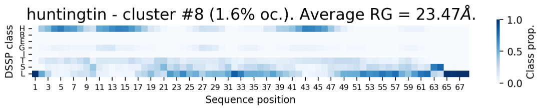

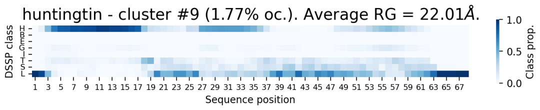

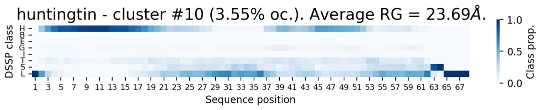

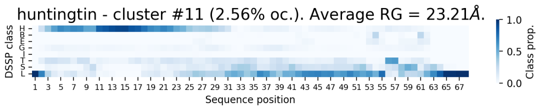

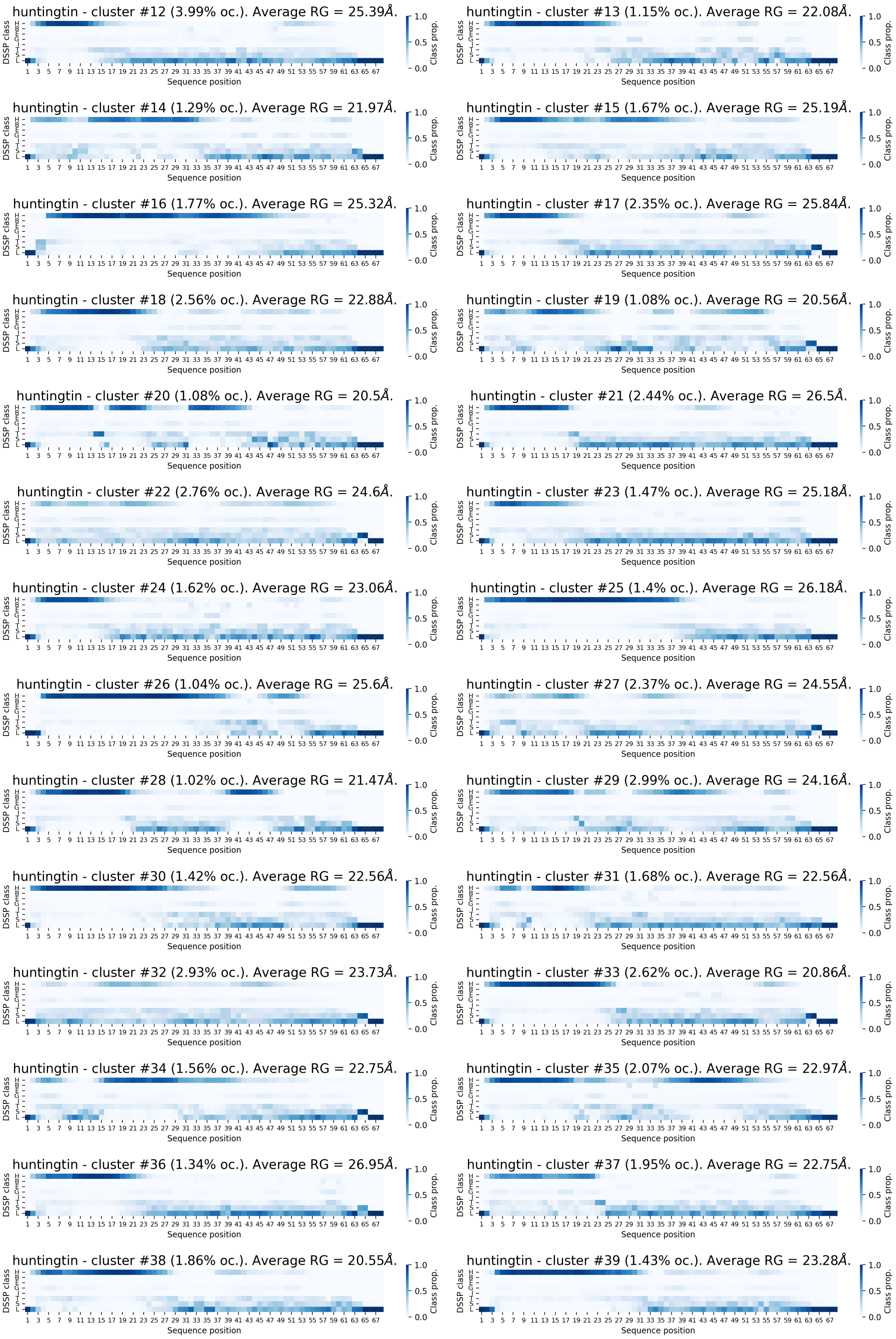
huntingtin - cluster #12 (3.99% oc.). Average RG = 25.39Å.
huntingtin - cluster #13 (1.15% oc.). Average RG = 22.08Å.
huntingtin - cluster #14 (1.29% oc.). Average RG = 21.97Å.
huntingtin - cluster #15 (1.67% oc.). Average RG = 25.19Å.
huntingtin - cluster #16 (1.77% oc.). Average RG = 25.32Å.
huntingtin - cluster #17 (2.35% oc.). Average RG = 25.84Å.
huntingtin - cluster #18 (2.56% oc.). Average RG = 22.88Å.
huntingtin - cluster #19 (1.08% oc.). Average RG = 20.56Å.
huntingtin - cluster #20 (1.08% oc.). Average RG = 20.5Å.
huntingtin - cluster #21 (2.44% oc.). Average RG = 26.5Å.
huntingtin - cluster #22 (2.76% oc.). Average RG = 24.6Å.
huntingtin - cluster #23 (1.47% oc.). Average RG = 25.18Å.
huntingtin - cluster #24 (1.62% oc.). Average RG = 23.06Å.
huntingtin - cluster #25 (1.4% oc.). Average RG = 26.18Å.
huntingtin - cluster #26 (1.04% oc.). Average RG = 25.6Å.
huntingtin - cluster #27 (2.37% oc.). Average RG = 24.55Å.
huntingtin - cluster #28 (1.02% oc.). Average RG = 21.47Å.
huntingtin - cluster #29 (2.99% oc.). Average RG = 24.16Å.
huntingtin - cluster #30 (1.42% oc.). Average RG = 22.56Å.
huntingtin - cluster #31 (1.68% oc.). Average RG = 22.56Å.
huntingtin - cluster #32 (2.93% oc.). Average RG = 23.73Å.
huntingtin - cluster #33 (2.62% oc.). Average RG = 20.86Å.
huntingtin - cluster #34 (1.56% oc.). Average RG = 22.75Å.
huntingtin - cluster #35 (2.07% oc.). Average RG = 22.97Å.
huntingtin - cluster #36 (1.34% oc.). Average RG = 26.95Å.
huntingtin - cluster #37 (1.95% oc.). Average RG = 22.75Å.
huntingtin - cluster #38 (1.86% oc.). Average RG = 20.55Å.
huntingtin - cluster #39 (1.43% oc.). Average RG = 23.28Å.
DSSP class
Sequence position
Class prop.

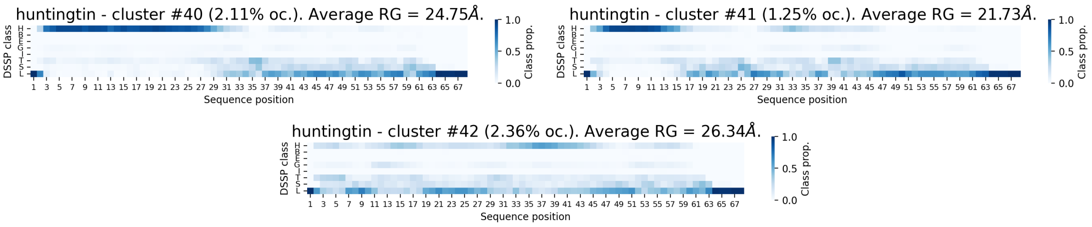

S6.3 Complete characterization of TDP-43 at 100 mM NaCl

S6.3.1 Two-dimensional UMAP projection

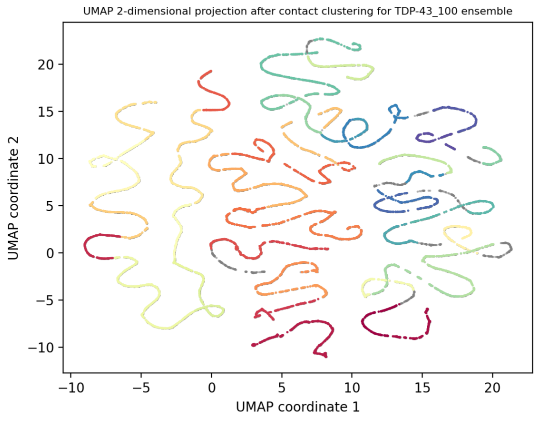

S6.3.2 Complete family of weighted ω-contact maps

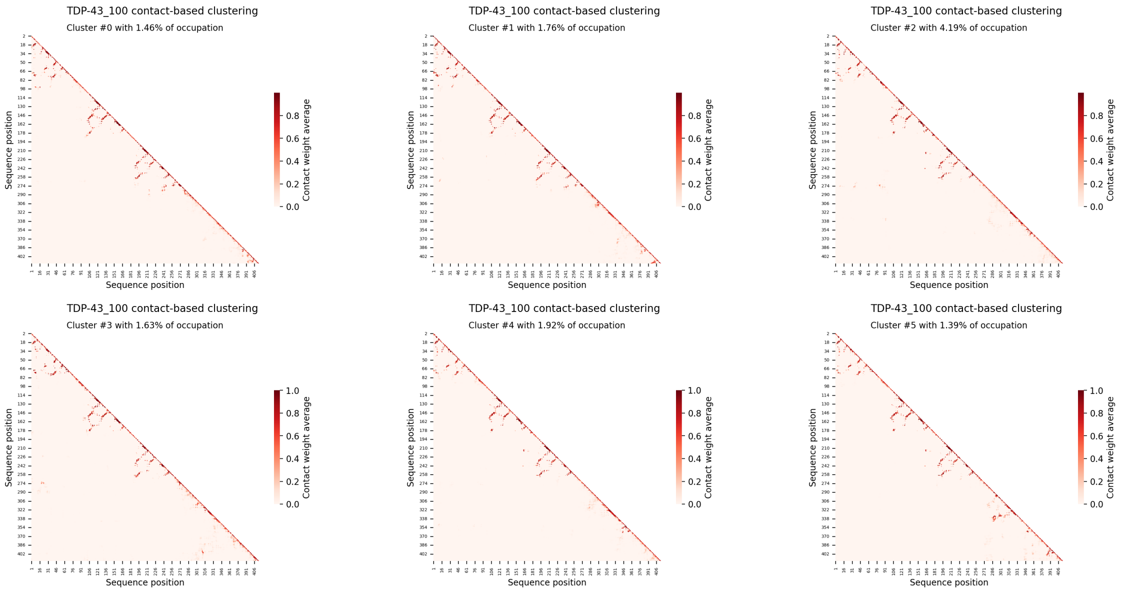

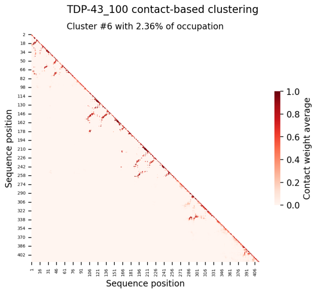
TDP-43_100 contact-based clustering
Cluster #6 with 2.36% of occupation
Sequence position
Sequence position
Contact weight average

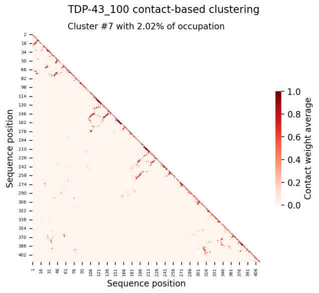
TDP-43_100 contact-based clustering
Cluster #7 with 2.02% of occupation
Sequence position
Sequence position
Contact weight average

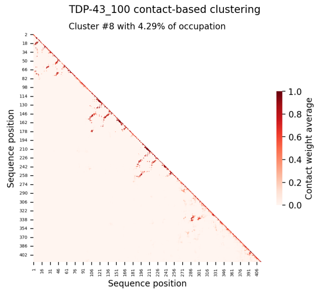
TDP-43_100 contact-based clustering
Cluster #8 with 4.29% of occupation
Sequence position
Sequence position
Contact weight average

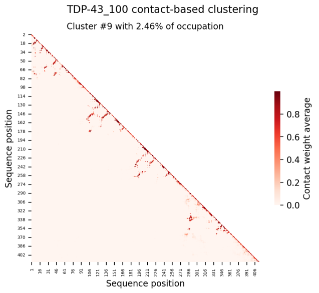
TDP-43_100 contact-based clustering
Cluster #9 with 2.46% of occupation
Sequence position
Sequence position
Contact weight average

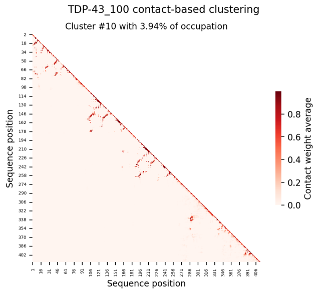
TDP-43_100 contact-based clustering
Cluster #10 with 3.94% of occupation
Sequence position
Sequence position
Contact weight average

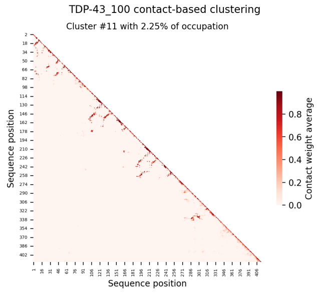
TDP-43_100 contact-based clustering
Cluster #11 with 2.25% of occupation
Sequence position
Sequence position
Contact weight average

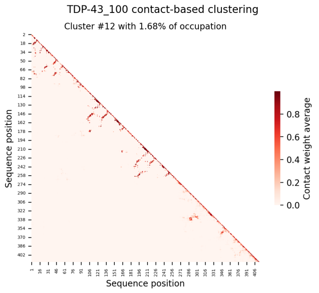
TDP-43_100 contact-based clustering
Cluster #12 with 1.68% of occupation
Sequence position
Sequence position
Contact weight average

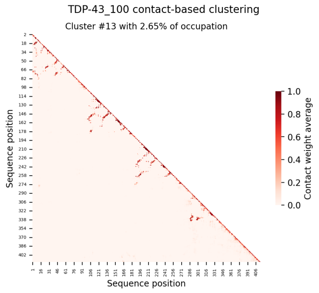
TDP-43_100 contact-based clustering
Cluster #13 with 2.65% of occupation
Sequence position
Sequence position
Contact weight average

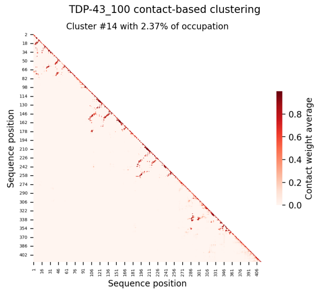
TDP-43_100 contact-based clustering
Cluster #14 with 2.37% of occupation
Sequence position
Sequence position
Contact weight average

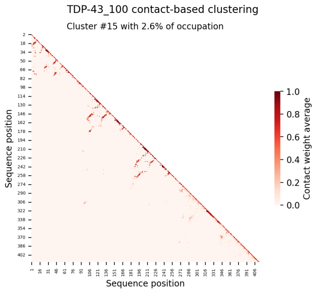
TDP-43_100 contact-based clustering
Cluster #15 with 2.6% of occupation
Sequence position
Sequence position
Contact weight average

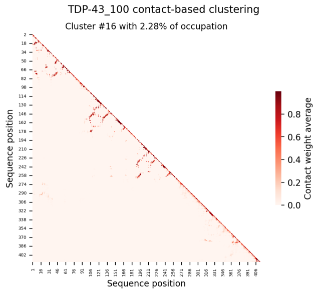
TDP-43_100 contact-based clustering
Cluster #16 with 2.28% of occupation
Sequence position
Sequence position
Contact weight average

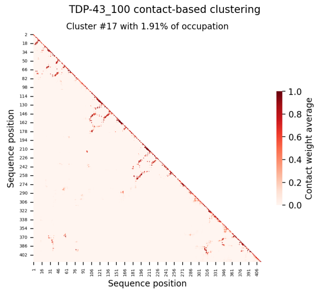
TDP-43_100 contact-based clustering
Cluster #17 with 1.91% of occupation
Sequence position
Sequence position
Contact weight average

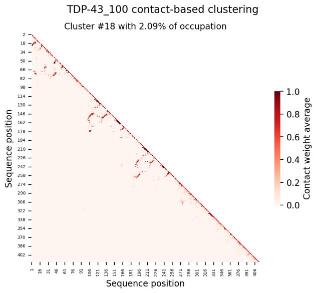
TDP-43_100 contact-based clustering
Cluster #18 with 2.09% of occupation
Sequence position
Sequence position
Contact weight average

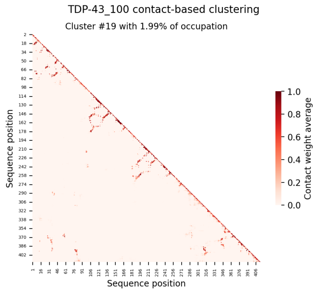
TDP-43_100 contact-based clustering
Cluster #19 with 1.99% of occupation
Sequence position
Sequence position
Contact weight average

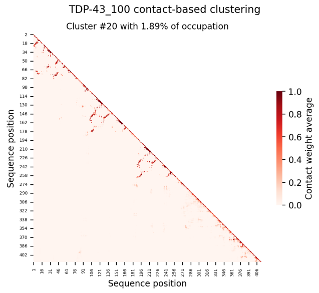
TDP-43_100 contact-based clustering
Cluster #20 with 1.89% of occupation
Sequence position
Sequence position
Contact weight average

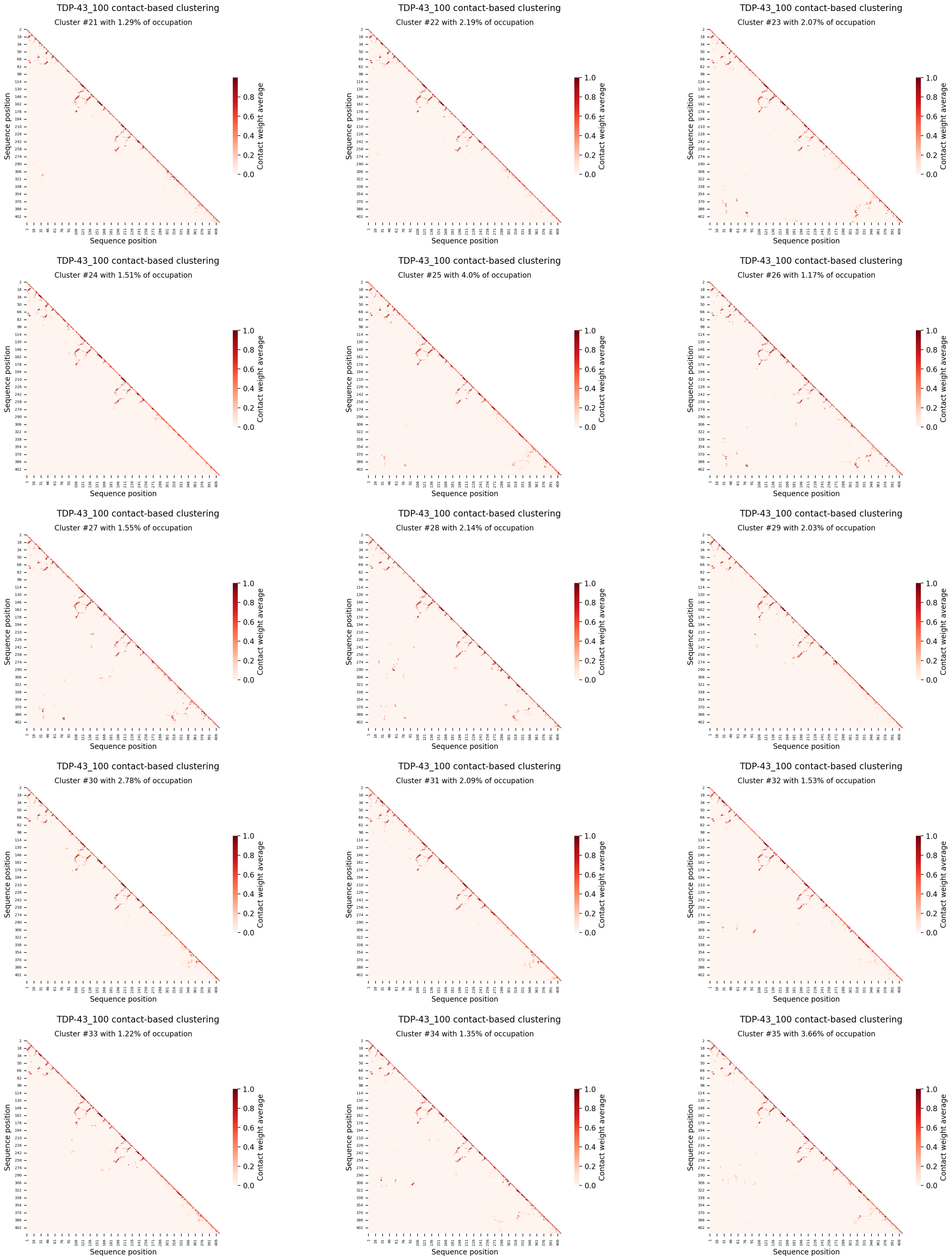
TDP-43_100 contact-based clustering
Cluster #21 with 1.29% of occupation
TDP-43_100 contact-based clustering
Cluster #22 with 2.19% of occupation
TDP-43_100 contact-based clustering
Cluster #23 with 2.07% of occupation
TDP-43_100 contact-based clustering
Cluster #24 with 1.51% of occupation
TDP-43_100 contact-based clustering
Cluster #25 with 4.0% of occupation
TDP-43_100 contact-based clustering
Cluster #26 with 1.17% of occupation
TDP-43_100 contact-based clustering
Cluster #27 with 1.55% of occupation
TDP-43_100 contact-based clustering
Cluster #28 with 2.14% of occupation
TDP-43_100 contact-based clustering
Cluster #29 with 2.03% of occupation
TDP-43_100 contact-based clustering
Cluster #30 with 2.78% of occupation
TDP-43_100 contact-based clustering
Cluster #31 with 2.09% of occupation
TDP-43_100 contact-based clustering
Cluster #32 with 1.53% of occupation
TDP-43_100 contact-based clustering
Cluster #33 with 1.22% of occupation
TDP-43_100 contact-based clustering
Cluster #34 with 1.35% of occupation
TDP-43_100 contact-based clustering
Cluster #35 with 3.66% of occupation
Sequence position
Contact weight average

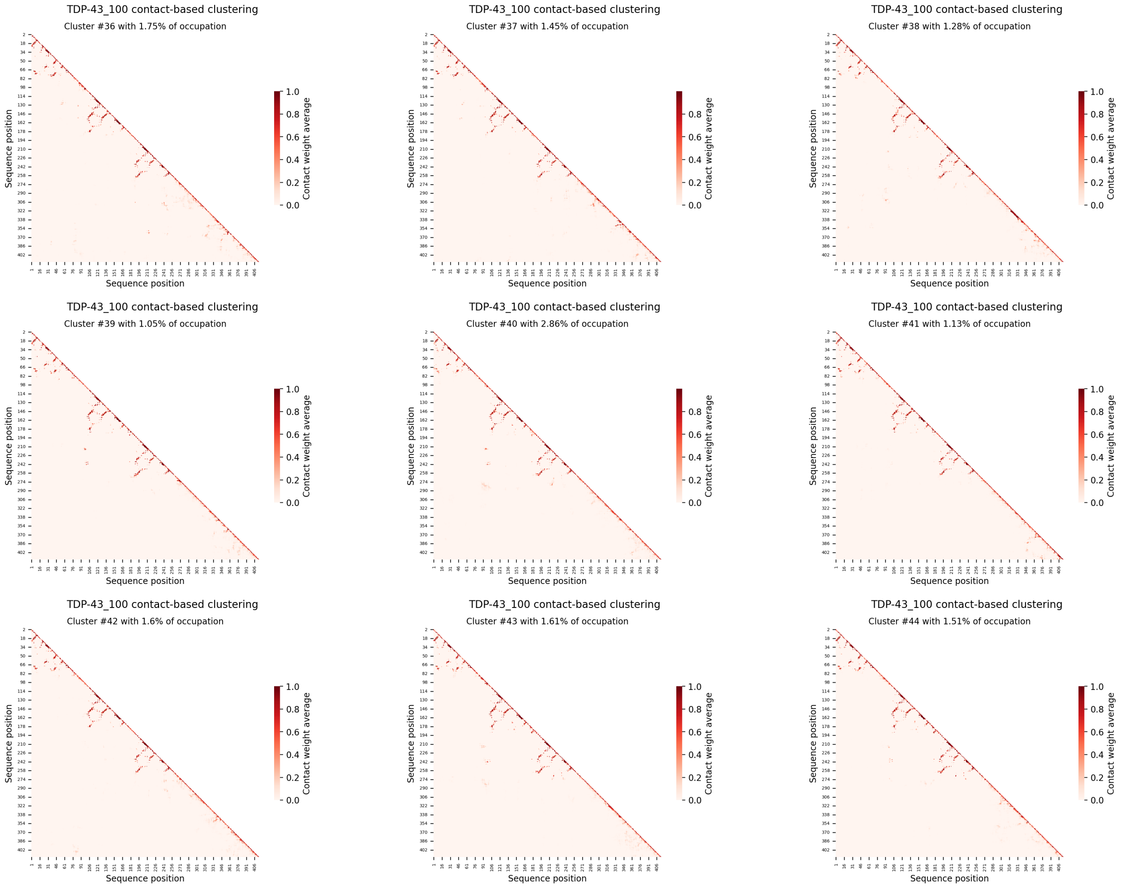

S6.3.3 Secondary structure propensities and average radii of gyration

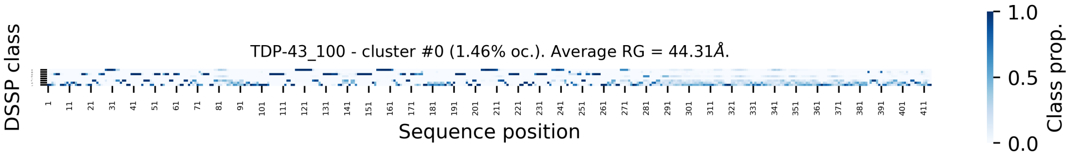

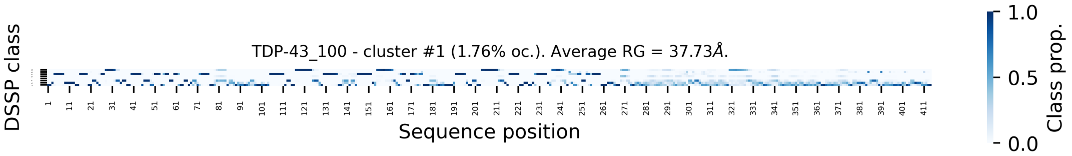

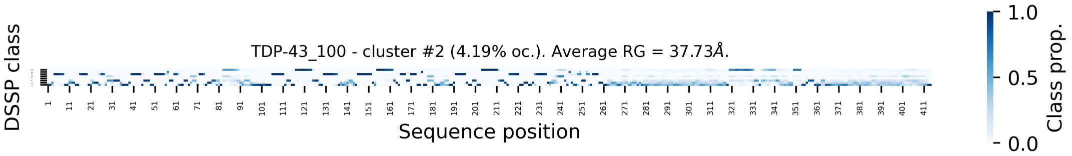

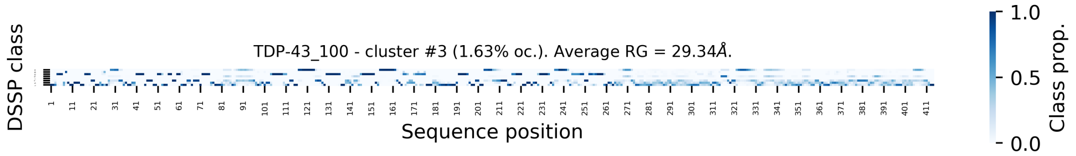
TDP-43_100 - cluster #3 (1.63% oc.). Average RG = 29.34Å.
DSSP class
Sequence position
Class prop.

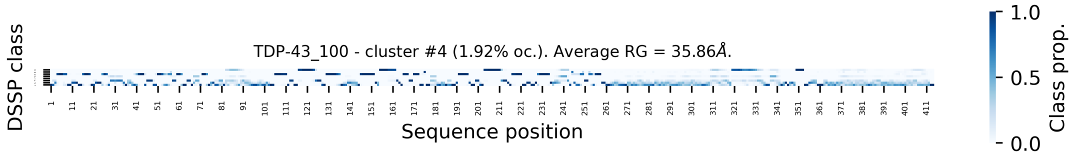
TDP-43_100 - cluster #4 (1.92% oc.). Average RG = 35.86Å.
DSSP class
Sequence position
Class prop.

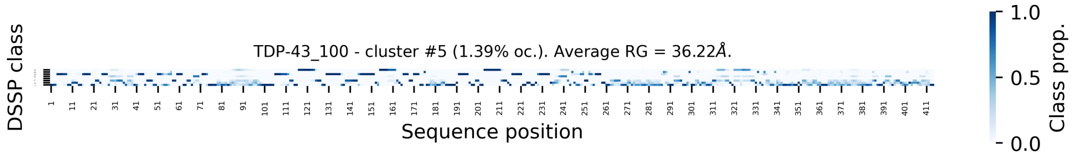
TDP-43_100 - cluster #5 (1.39% oc.). Average RG = 36.22Å.
DSSP class
Sequence position
Class prop.

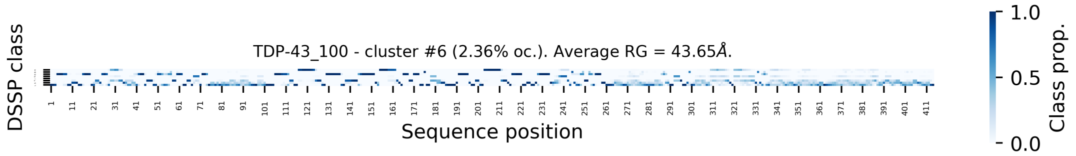
TDP-43_100 - cluster #6 (2.36% oc.). Average RG = 43.65Å.
DSSP class
Sequence position
Class prop.

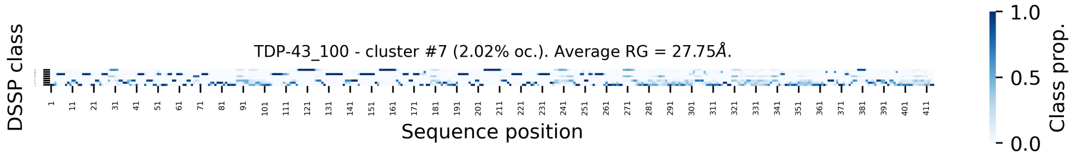
TDP-43_100 - cluster #7 (2.02% oc.). Average RG = 27.75Å.
DSSP class
Sequence position
Class prop.

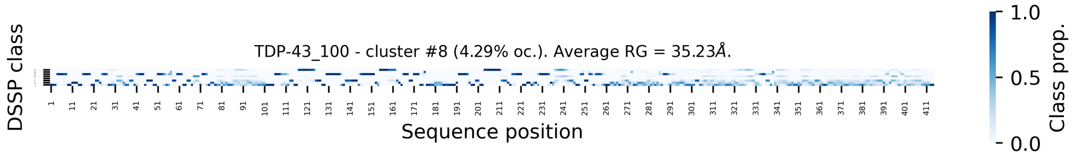
TDP-43_100 - cluster #8 (4.29% oc.). Average RG = 35.23Å.
DSSP class
Sequence position
Class prop.

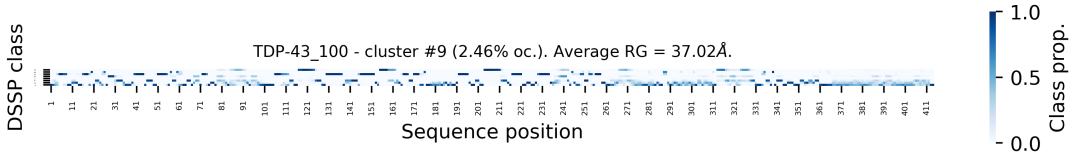
TDP-43_100 - cluster #9 (2.46% oc.). Average RG = 37.02Å.
DSSP class
Sequence position
Class prop.

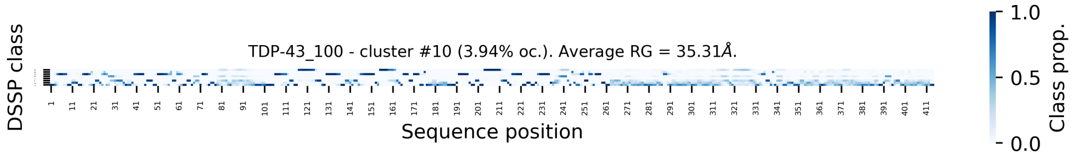
TDP-43_100 - cluster #10 (3.94% oc.). Average RG = 35.31Å.
DSSP class
Sequence position
Class prop.

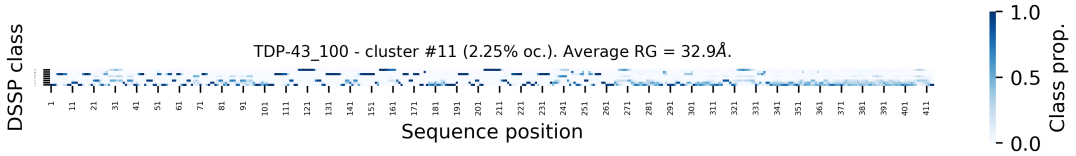
TDP-43_100 - cluster #11 (2.25% oc.). Average RG = 32.9Å.
DSSP class
Sequence position
Class prop.

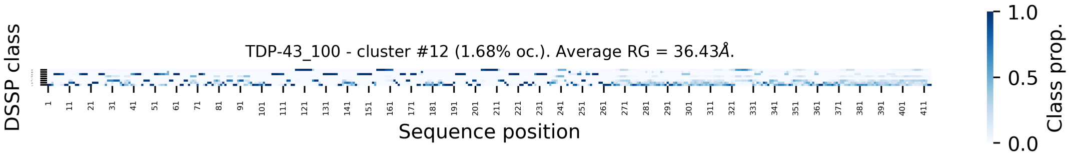
TDP-43_100 - cluster #12 (1.68% oc.). Average RG = 36.43Å.
DSSP class
Sequence position
Class prop.

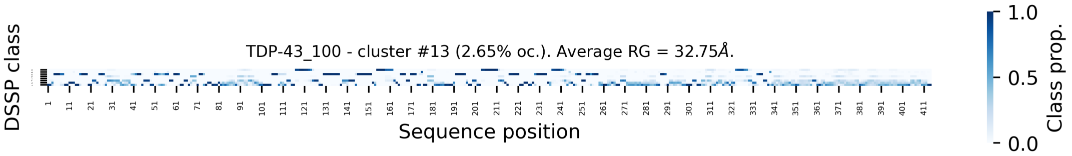
TDP-43_100 - cluster #13 (2.65% oc.). Average RG = 32.75Å.
DSSP class
Sequence position
Class prop.

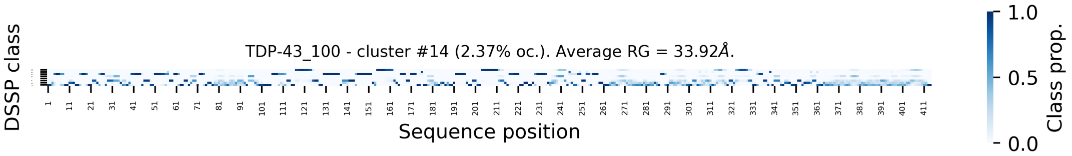
TDP-43_100 - cluster #14 (2.37% oc.). Average RG = 33.92Å.
DSSP class
Sequence position
Class prop.

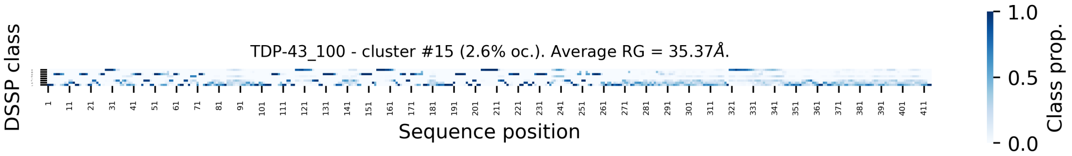
TDP-43_100 - cluster #15 (2.6% oc.). Average RG = 35.37Å.
DSSP class
Sequence position
Class prop.

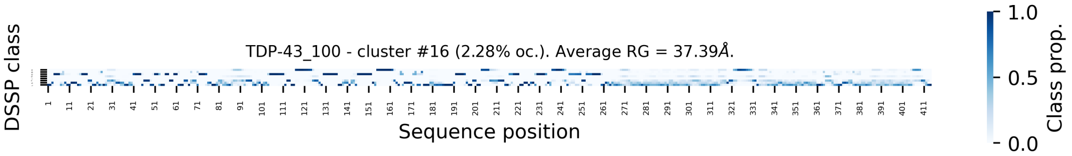
TDP-43_100 - cluster #16 (2.28% oc.). Average RG = 37.39Å.
DSSP class
Sequence position
Class prop.

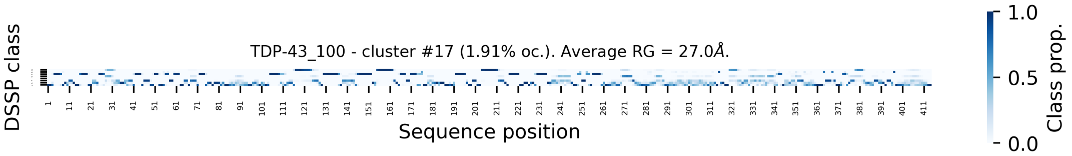
TDP-43_100 - cluster #17 (1.91% oc.). Average RG = 27.0Å.
DSSP class
Sequence position
Class prop.

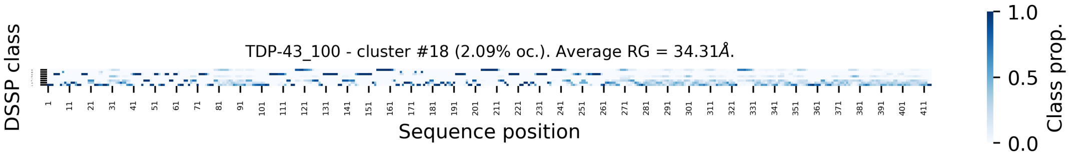
TDP-43_100 - cluster #18 (2.09% oc.). Average RG = 34.31Å.
DSSP class
Sequence position
Class prop.

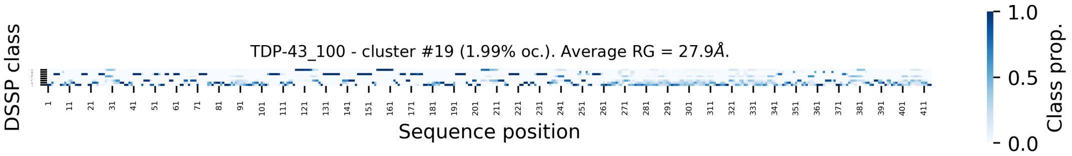
TDP-43_100 - cluster #19 (1.99% oc.). Average RG = 27.9Å.
DSSP class
Sequence position
Class prop.

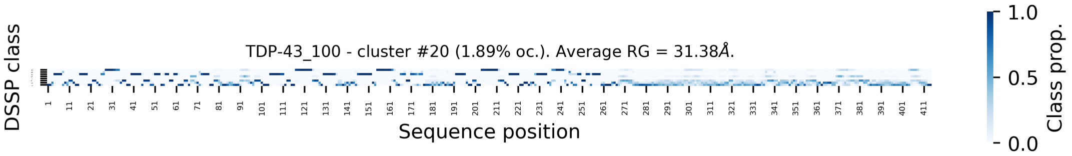
TDP-43_100 - cluster #20 (1.89% oc.). Average RG = 31.38Å.
DSSP class
Sequence position
Class prop.

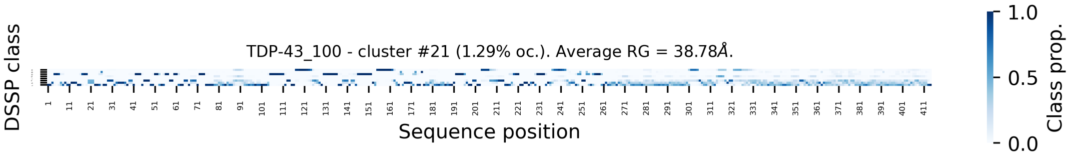
TDP-43_100 - cluster #21 (1.29% oc.). Average RG = 38.78Å.
DSSP class
Sequence position
Class prop.
1.0
0.5
0.0

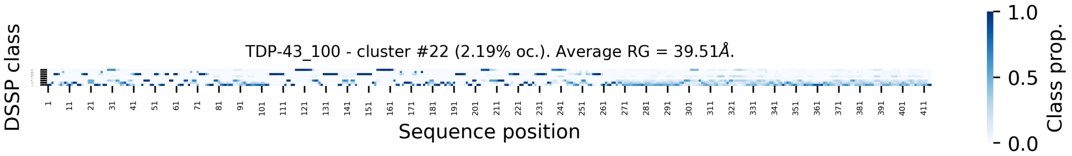
TDP-43_100 - cluster #22 (2.19% oc.). Average RG = 39.51Å.
DSSP class
Sequence position
Class prop.
1.0
0.5
0.0

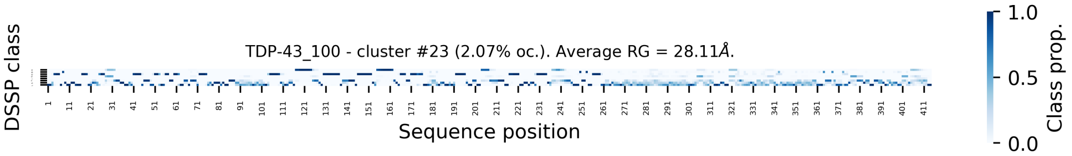
TDP-43_100 - cluster #23 (2.07% oc.). Average RG = 28.11Å.
DSSP class
Sequence position
Class prop.
1.0
0.5
0.0

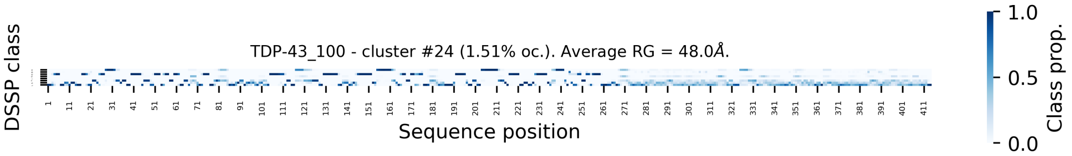
TDP-43_100 - cluster #24 (1.51% oc.). Average RG = 48.0Å.
DSSP class
Sequence position
Class prop.
1.0
0.5
0.0

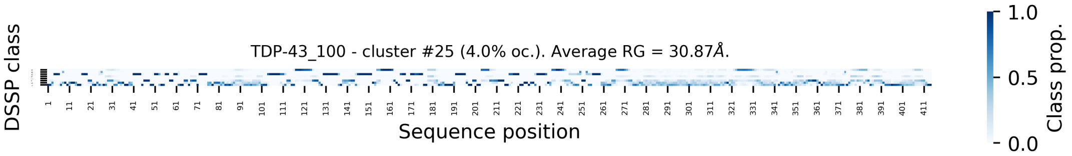
TDP-43_100 - cluster #25 (4.0% oc.). Average RG = 30.87Å.
DSSP class
Sequence position
Class prop.
1.0
0.5
0.0

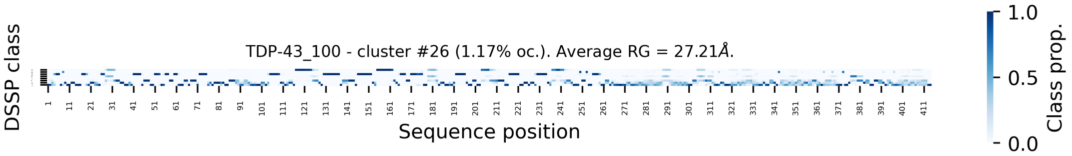
TDP-43_100 - cluster #26 (1.17% oc.). Average RG = 27.21Å.
DSSP class
Sequence position
Class prop.
1.0
0.5
0.0

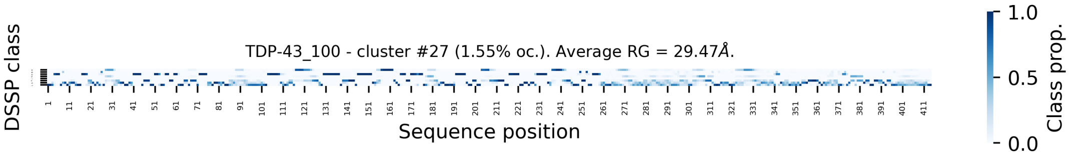
TDP-43_100 - cluster #27 (1.55% oc.). Average RG = 29.47Å.
DSSP class
Sequence position
Class prop.
1.0
0.5
0.0

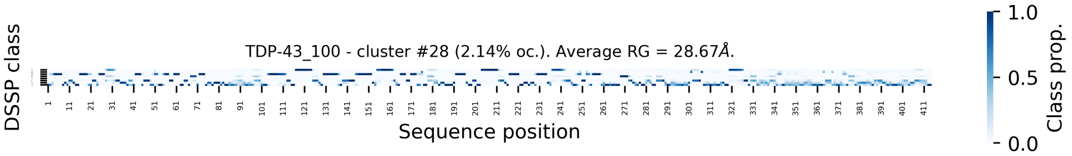
TDP-43_100 - cluster #28 (2.14% oc.). Average RG = 28.67Å.
DSSP class
Sequence position
Class prop.
1.0
0.5
0.0

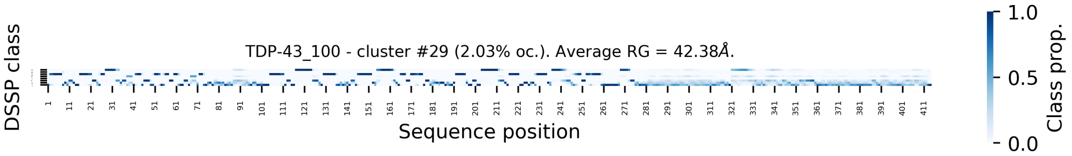
TDP-43_100 - cluster #29 (2.03% oc.). Average RG = 42.38Å.
DSSP class
Sequence position
Class prop.
1.0
0.5
0.0

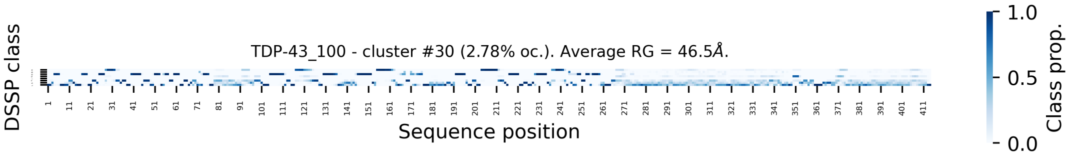
TDP-43_100 - cluster #30 (2.78% oc.). Average RG = 46.5Å.
DSSP class
Sequence position
Class prop.
1.0
0.5
0.0

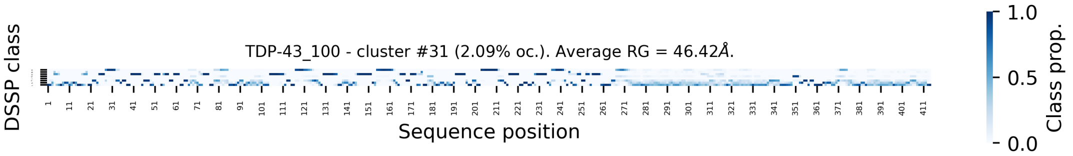
TDP-43_100 - cluster #31 (2.09% oc.). Average RG = 46.42Å.
DSSP class
Sequence position
Class prop.
1.0
0.5
0.0

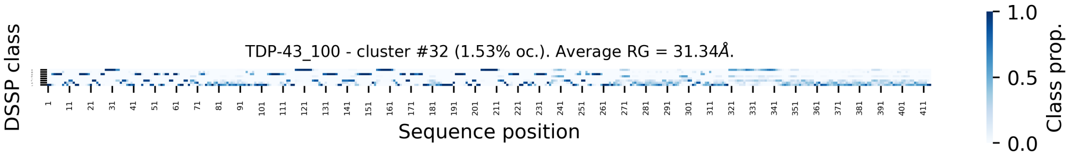
TDP-43_100 - cluster #32 (1.53% oc.). Average RG = 31.34Å.
DSSP class
Sequence position
Class prop.
1.0
0.5
0.0

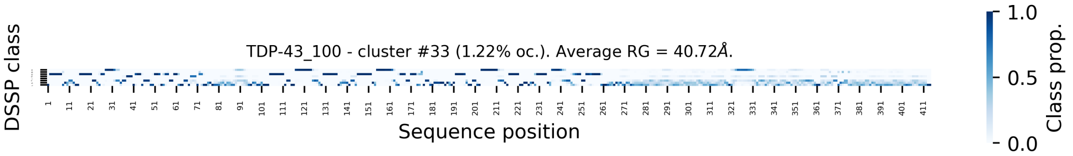
TDP-43_100 - cluster #33 (1.22% oc.). Average RG = 40.72Å.
DSSP class
Sequence position
Class prop.
1.0
0.5
0.0

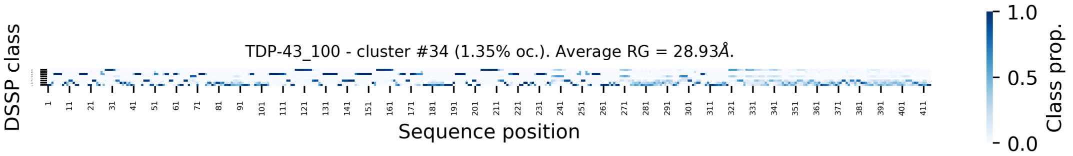
TDP-43_100 - cluster #34 (1.35% oc.). Average RG = 28.93Å.
DSSP class
Sequence position
Class prop.
1.0
0.5
0.0

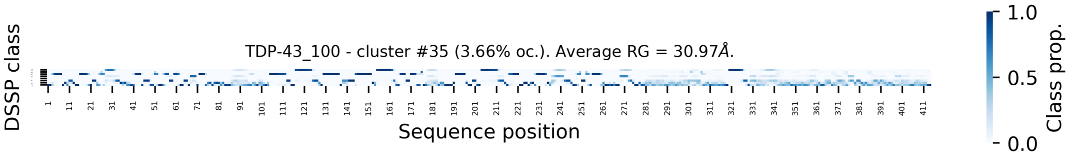
TDP-43_100 - cluster #35 (3.66% oc.). Average RG = 30.97Å.
DSSP class
Sequence position
Class prop.
1.0
0.5
0.0

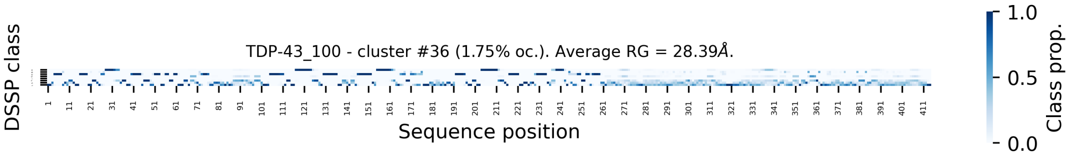
TDP-43_100 - cluster #36 (1.75% oc.). Average RG = 28.39Å.
DSSP class
Sequence position
Class prop.
1.0
0.5
0.0

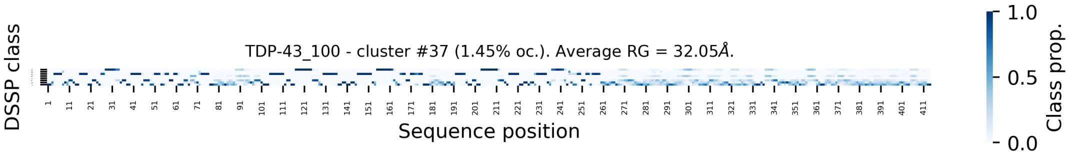
TDP-43_100 - cluster #37 (1.45% oc.). Average RG = 32.05Å.
DSSP class
Sequence position
Class prop.
1.0
0.5
0.0

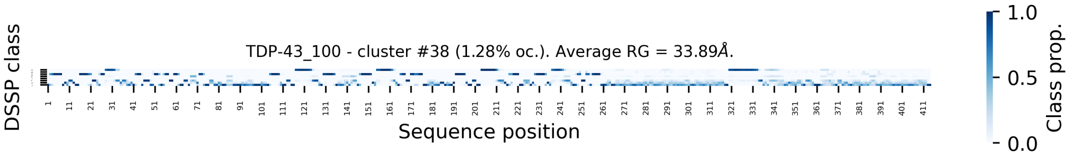
TDP-43_100 - cluster #38 (1.28% oc.). Average RG = 33.89Å.
DSSP class
Sequence position
Class prop.
1.0
0.5
0.0

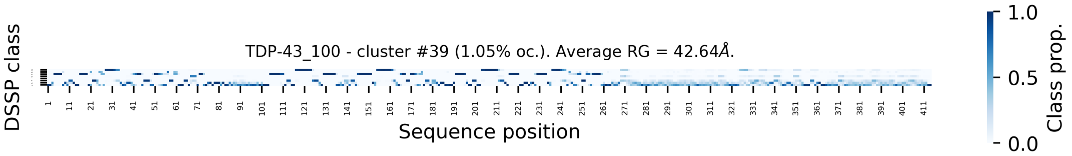
DSSP class
TDP-43_100 - cluster #39 (1.05% oc.). Average RG = 42.64Å.
Sequence position
Class prop.
1.0
0.5
0.0

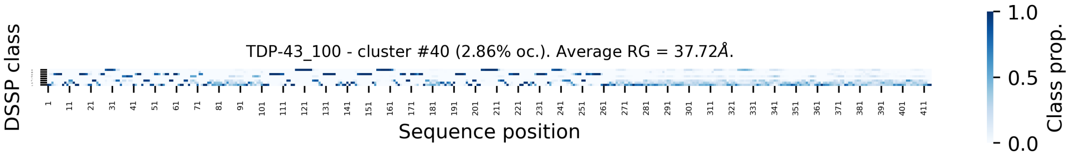
DSSP class
TDP-43_100 - cluster #40 (2.86% oc.). Average RG = 37.72Å.
Sequence position
Class prop.
1.0
0.5
0.0

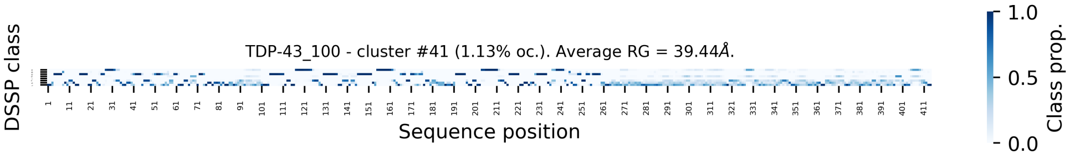
DSSP class
TDP-43_100 - cluster #41 (1.13% oc.). Average RG = 39.44Å.
Sequence position
Class prop.
1.0
0.5
0.0

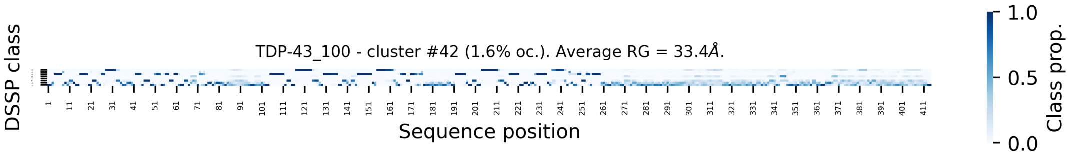
DSSP class
TDP-43_100 - cluster #42 (1.6% oc.). Average RG = 33.4Å.
Sequence position
Class prop.
1.0
0.5
0.0

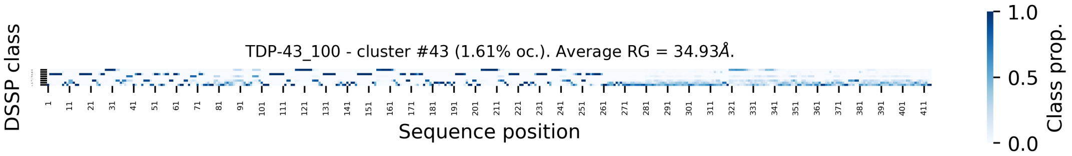
DSSP class
TDP-43_100 - cluster #43 (1.61% oc.). Average RG = 34.93Å.
Sequence position
Class prop.
1.0
0.5
0.0

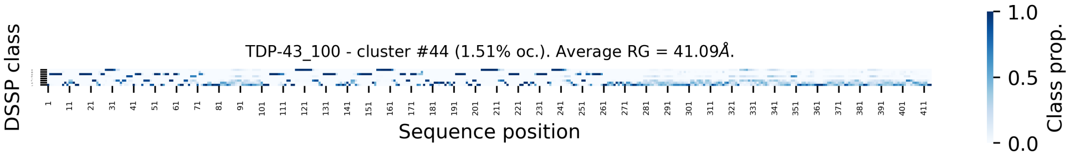
DSSP class
TDP-43_100 - cluster #44 (1.51% oc.). Average RG = 41.09Å.
Sequence position
Class prop.
1.0
0.5
0.0

S6.4 Complete characterization of TDP-43 at 300 mM NaCl

S6.4.1 Two-dimensional UMAP projection

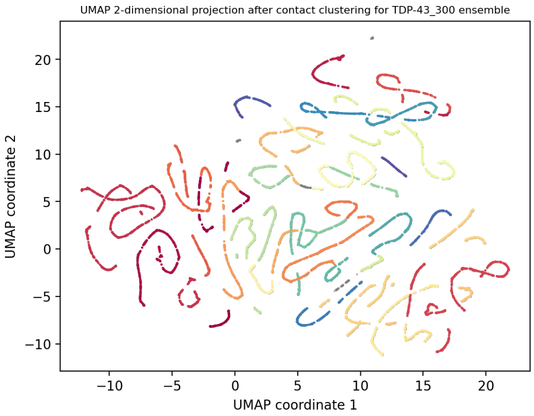

S6.4.2 Complete family of weighted ω-contact maps

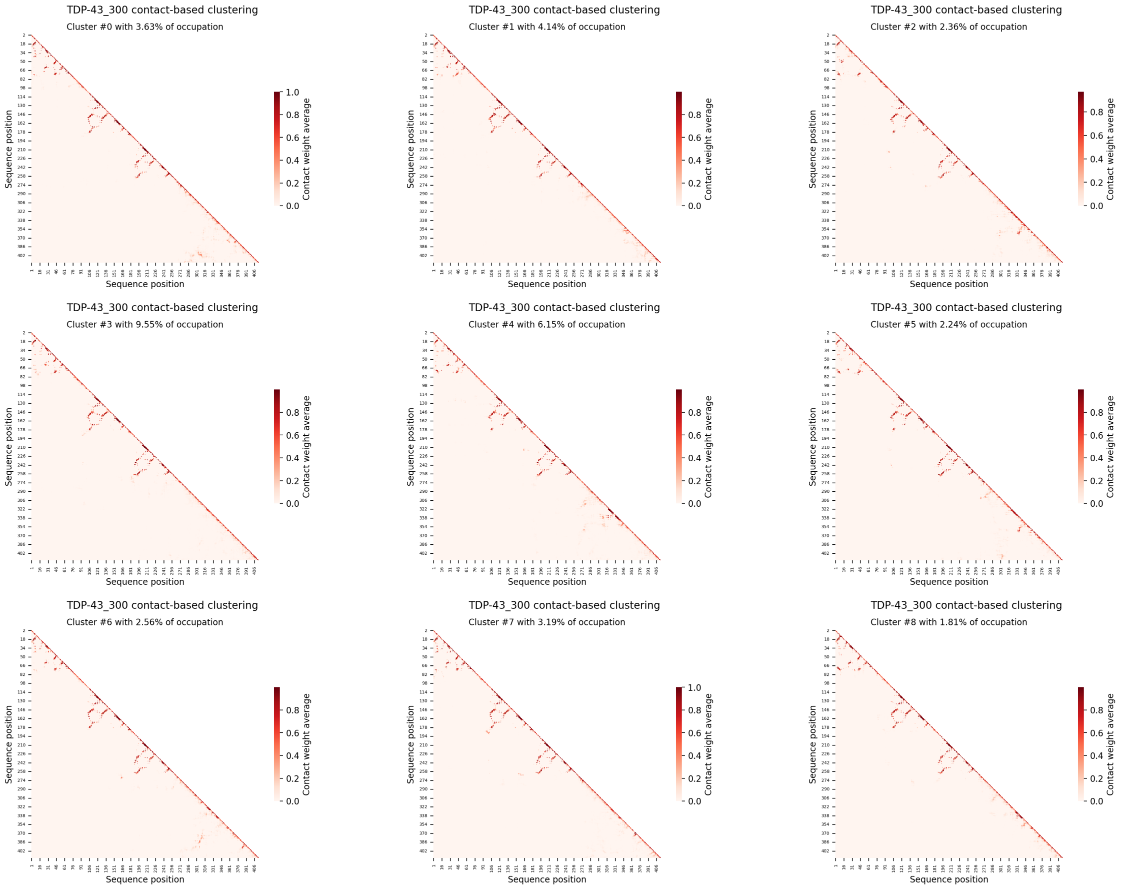

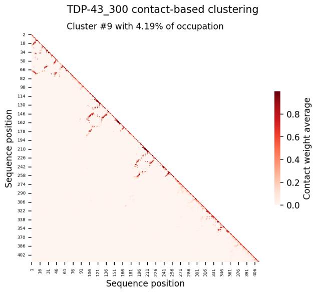
TDP-43_300 contact-based clustering
Cluster #9 with 4.19% of occupation
Sequence position
Sequence position
Contact weight average

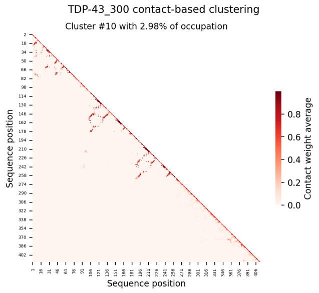
TDP-43_300 contact-based clustering
Cluster #10 with 2.98% of occupation
Sequence position
Sequence position
Contact weight average

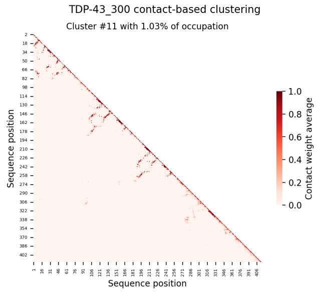
TDP-43_300 contact-based clustering
Cluster #11 with 1.03% of occupation
Sequence position
Sequence position
Contact weight average

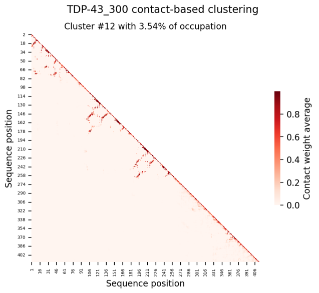
TDP-43_300 contact-based clustering
Cluster #12 with 3.54% of occupation
Sequence position
Sequence position
Contact weight average

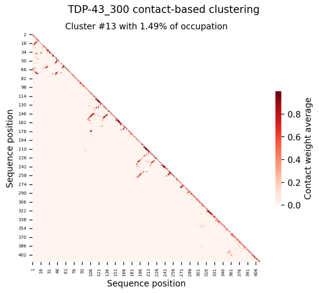
TDP-43_300 contact-based clustering
Cluster #13 with 1.49% of occupation
Sequence position
Sequence position
Contact weight average

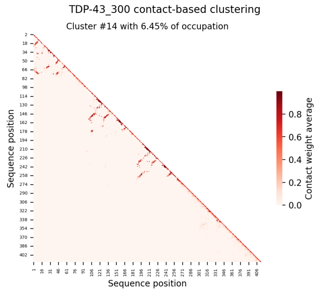
TDP-43_300 contact-based clustering
Cluster #14 with 6.45% of occupation
Sequence position
Sequence position
Contact weight average

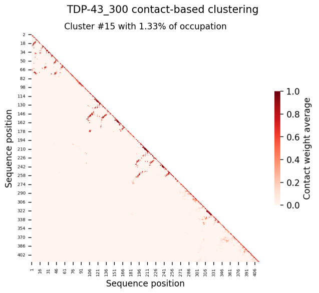
TDP-43_300 contact-based clustering
Cluster #15 with 1.33% of occupation
Sequence position
Sequence position
Contact weight average

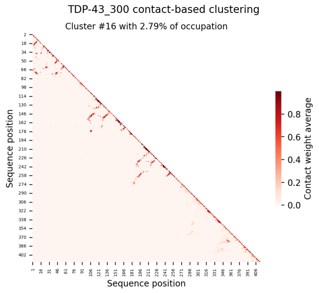
TDP-43_300 contact-based clustering
Cluster #16 with 2.79% of occupation
Sequence position
Sequence position
Contact weight average

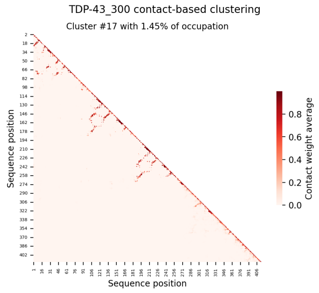
TDP-43_300 contact-based clustering
Cluster #17 with 1.45% of occupation
Sequence position
Sequence position
Contact weight average

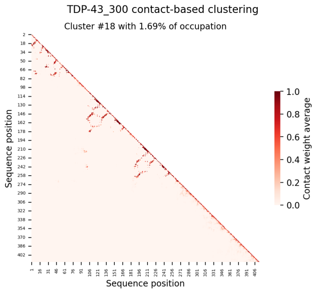
TDP-43_300 contact-based clustering
Cluster #18 with 1.69% of occupation
Sequence position
Sequence position
Contact weight average

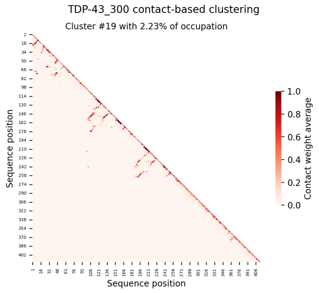
TDP-43_300 contact-based clustering
Cluster #19 with 2.23% of occupation
Sequence position
Sequence position
Contact weight average

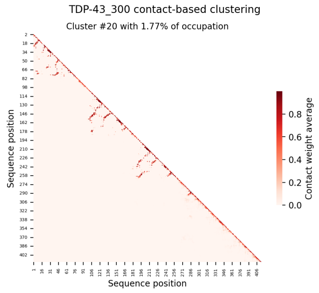
TDP-43_300 contact-based clustering
Cluster #20 with 1.77% of occupation
Sequence position
Sequence position
Contact weight average

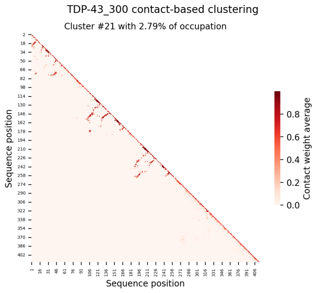
TDP-43_300 contact-based clustering
Cluster #21 with 2.79% of occupation
Sequence position
Sequence position
Contact weight average

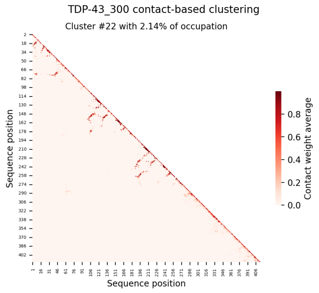
TDP-43_300 contact-based clustering
Cluster #22 with 2.14% of occupation
Sequence position
Sequence position
Contact weight average

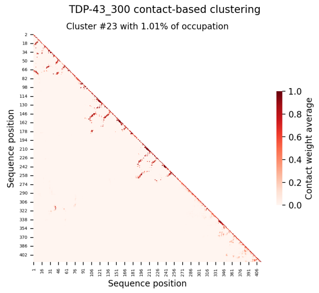
TDP-43_300 contact-based clustering
Cluster #23 with 1.01% of occupation
Sequence position
Sequence position
Contact weight average

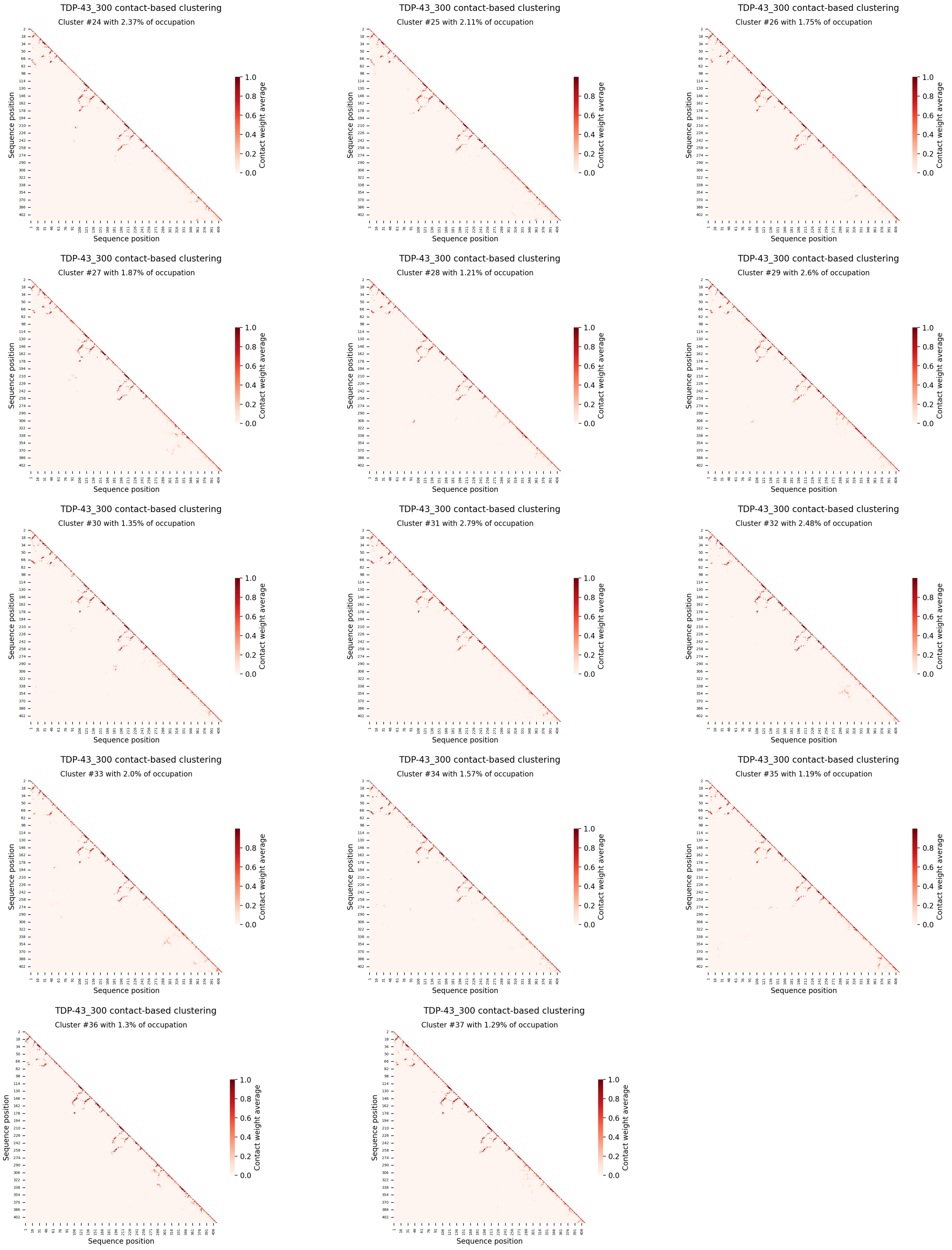
TDP-43_300 contact-based clustering
Cluster #24 with 2.37% of occupation
TDP-43_300 contact-based clustering
Cluster #25 with 2.11% of occupation
TDP-43_300 contact-based clustering
Cluster #26 with 1.75% of occupation
TDP-43_300 contact-based clustering
Cluster #27 with 1.87% of occupation
TDP-43_300 contact-based clustering
Cluster #28 with 1.21% of occupation
TDP-43_300 contact-based clustering
Cluster #29 with 2.6% of occupation
TDP-43_300 contact-based clustering
Cluster #30 with 1.35% of occupation
TDP-43_300 contact-based clustering
Cluster #31 with 2.79% of occupation
TDP-43_300 contact-based clustering
Cluster #32 with 2.48% of occupation
TDP-43_300 contact-based clustering
Cluster #33 with 2.0% of occupation
TDP-43_300 contact-based clustering
Cluster #34 with 1.57% of occupation
TDP-43_300 contact-based clustering
Cluster #35 with 1.19% of occupation
TDP-43_300 contact-based clustering
Cluster #36 with 1.3% of occupation
TDP-43_300 contact-based clustering
Cluster #37 with 1.29% of occupation
Sequence position
Contact weight average

S6.4.3 Secondary structure propensities and average radii of gyration

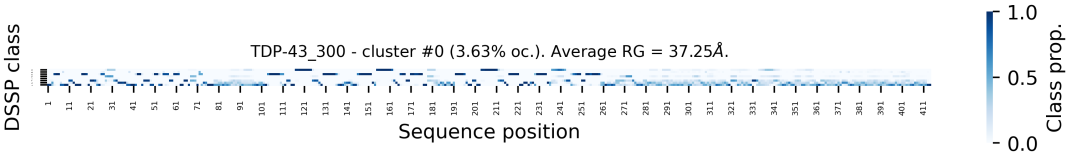

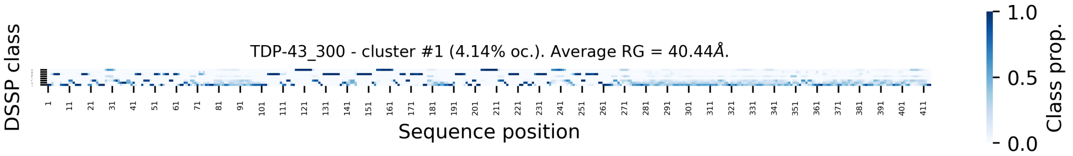

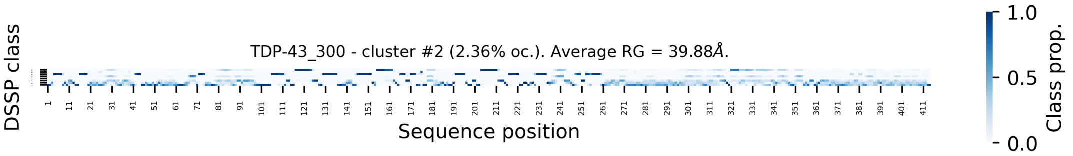

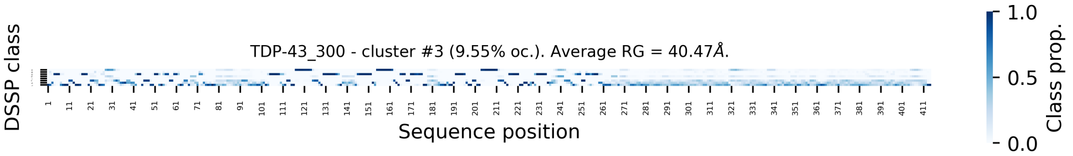

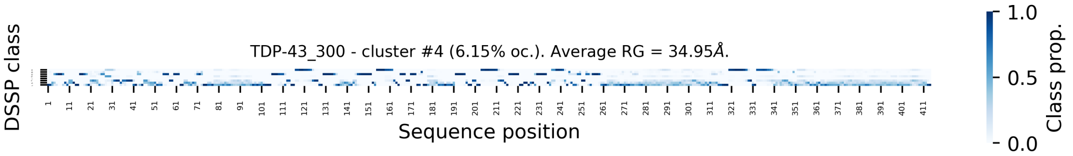

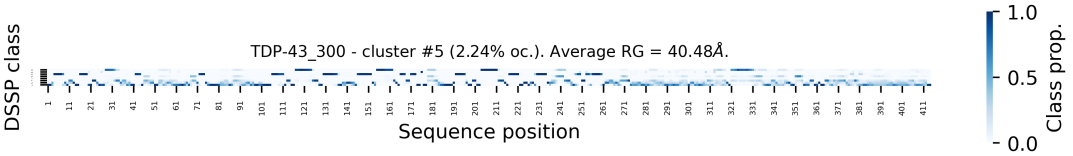

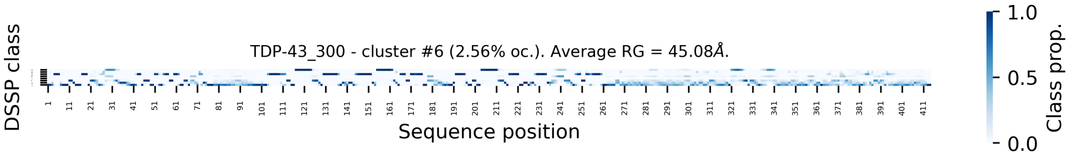

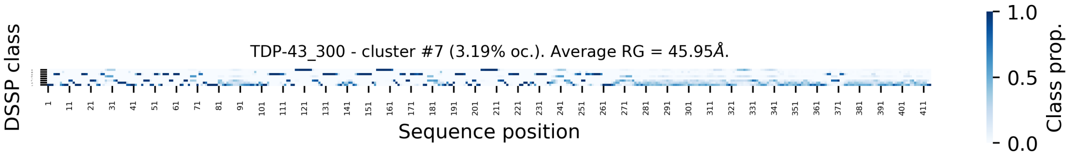

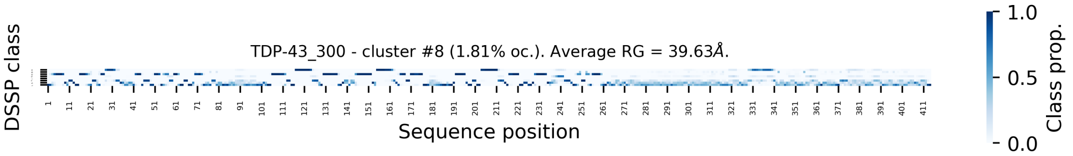

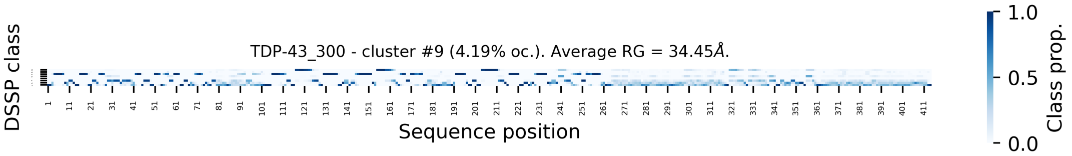
TDP-43_300 - cluster #9 (4.19% oc.). Average RG = 34.45Å.
DSSP class
Sequence position
Class prop.
1.0
0.5
0.0

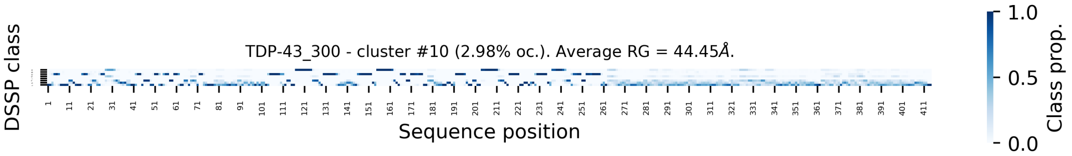
TDP-43_300 - cluster #10 (2.98% oc.). Average RG = 44.45Å.
DSSP class
Sequence position
Class prop.
1.0
0.5
0.0

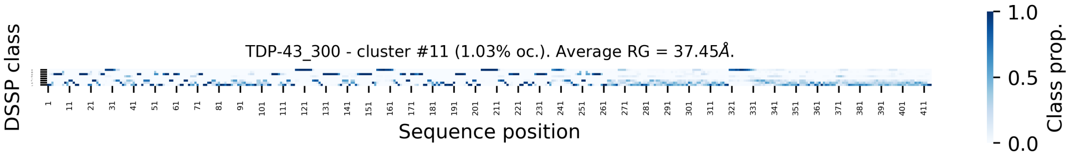
TDP-43_300 - cluster #11 (1.03% oc.). Average RG = 37.45Å.
DSSP class
Sequence position
Class prop.
1.0
0.5
0.0

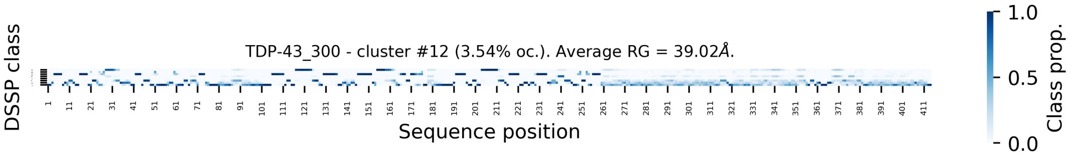
TDP-43_300 - cluster #12 (3.54% oc.). Average RG = 39.02Å.
DSSP class
Sequence position
Class prop.
1.0
0.5
0.0

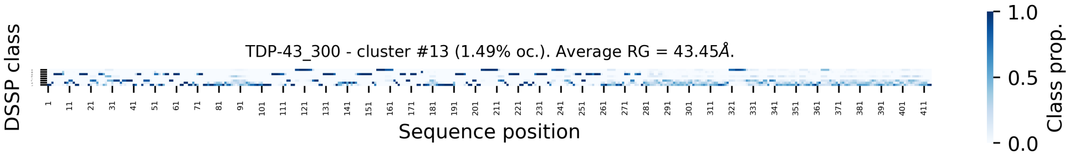
TDP-43_300 - cluster #13 (1.49% oc.). Average RG = 43.45Å.
DSSP class
Sequence position
Class prop.
1.0
0.5
0.0

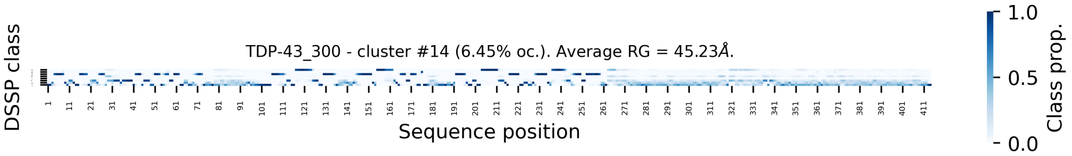
TDP-43_300 - cluster #14 (6.45% oc.). Average RG = 45.23Å.
DSSP class
Sequence position
Class prop.
1.0
0.5
0.0

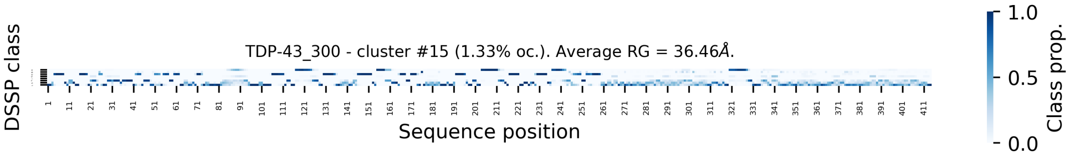
TDP-43_300 - cluster #15 (1.33% oc.). Average RG = 36.46Å.
DSSP class
Sequence position
Class prop.
1.0
0.5
0.0

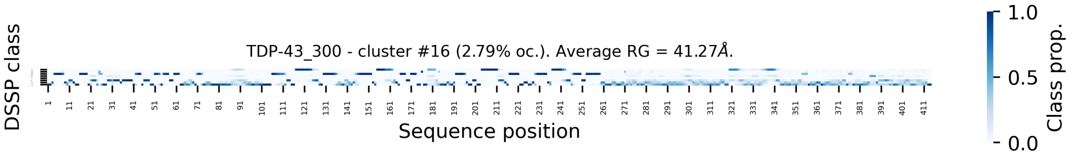
TDP-43_300 - cluster #16 (2.79% oc.). Average RG = 41.27Å.
DSSP class
Sequence position
Class prop.
1.0
0.5
0.0

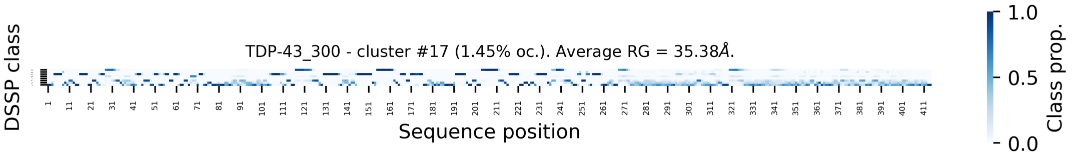
TDP-43_300 - cluster #17 (1.45% oc.). Average RG = 35.38Å.
DSSP class
Sequence position
Class prop.
1.0
0.5
0.0

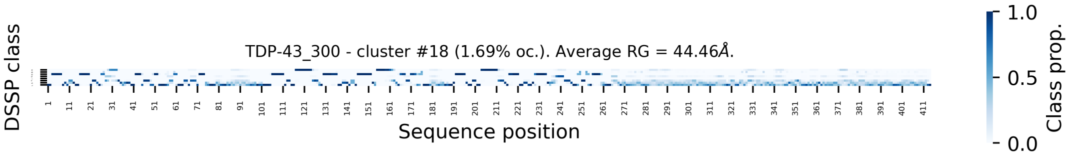
TDP-43_300 - cluster #18 (1.69% oc.). Average RG = 44.46Å.
DSSP class
Sequence position
Class prop.
1.0
0.5
0.0

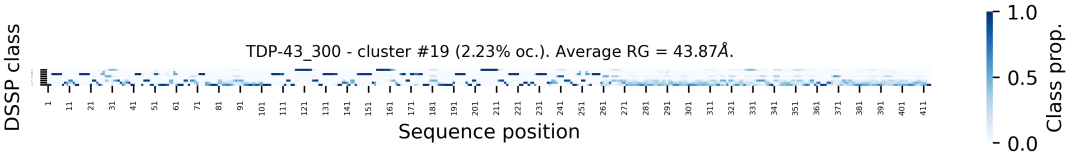
TDP-43_300 - cluster #19 (2.23% oc.). Average RG = 43.87Å.
DSSP class
Sequence position
Class prop.
1.0
0.5
0.0

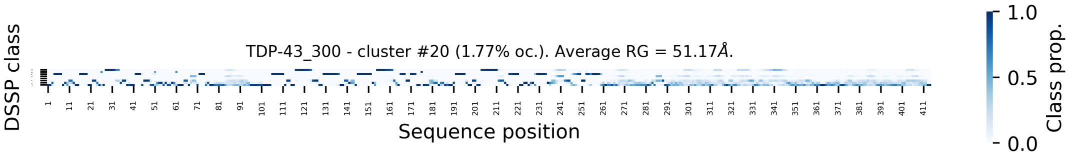
TDP-43_300 - cluster #20 (1.77% oc.). Average RG = 51.17Å.
DSSP class
Sequence position
Class prop.
1.0
0.5
0.0

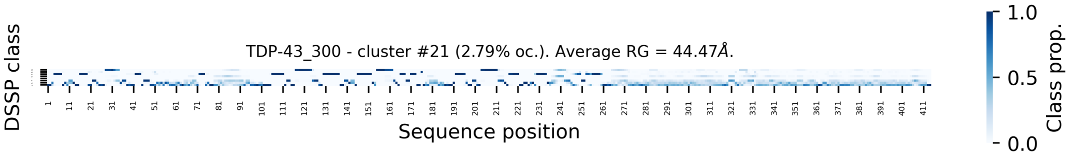
TDP-43_300 - cluster #21 (2.79% oc.). Average RG = 44.47Å.
DSSP class
Sequence position
Class prop.
1.0
0.5
0.0

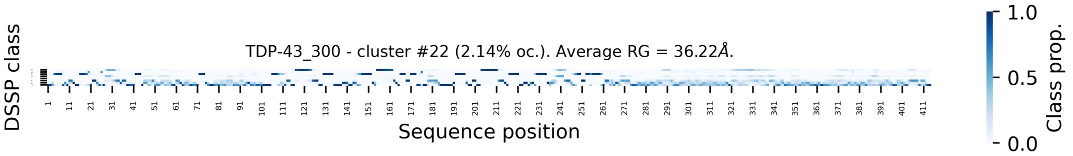
TDP-43_300 - cluster #22 (2.14% oc.). Average RG = 36.22Å.
DSSP class
Sequence position
Class prop.
1.0
0.5
0.0

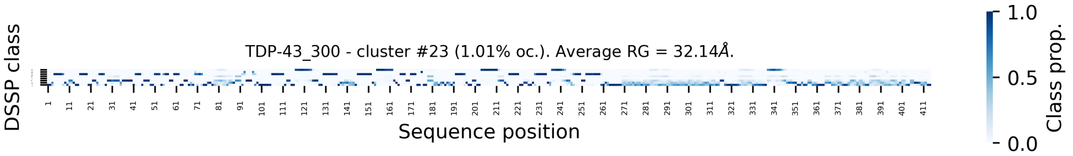
TDP-43_300 - cluster #23 (1.01% oc.). Average RG = 32.14Å.
DSSP class
Sequence position
Class prop.
1.0
0.5
0.0

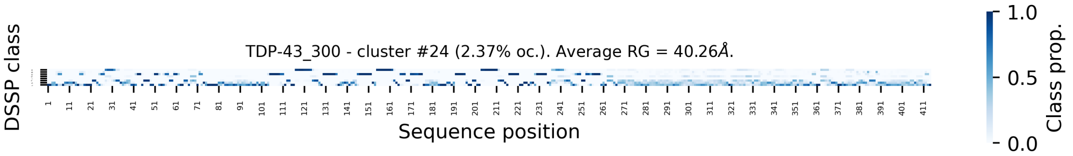
TDP-43_300 - cluster #24 (2.37% oc.). Average RG = 40.26Å.
DSSP class
Sequence position
Class prop.
1.0
0.5
0.0

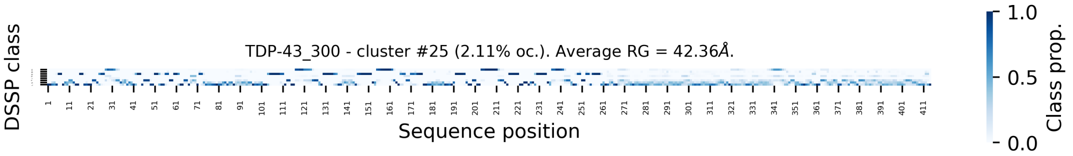
TDP-43_300 - cluster #25 (2.11% oc.). Average RG = 42.36Å.
DSSP class
Sequence position
Class prop.
1.0
0.5
0.0

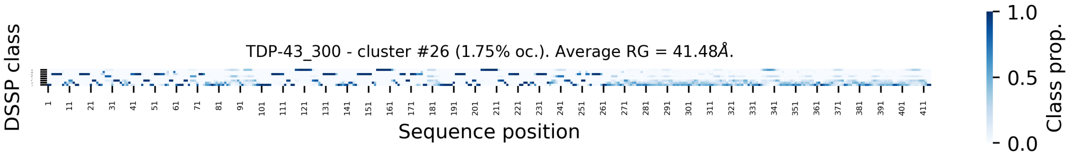
TDP-43_300 - cluster #26 (1.75% oc.). Average RG = 41.48Å.
DSSP class
Sequence position
Class prop.
1.0
0.5
0.0

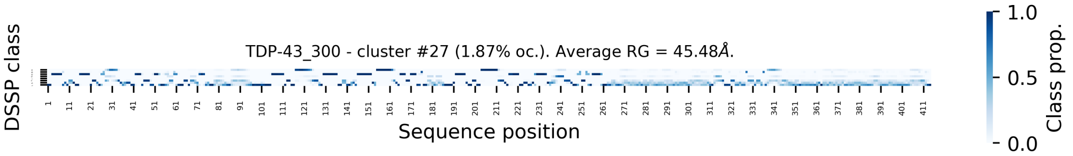
TDP-43_300 - cluster #27 (1.87% oc.). Average RG = 45.48Å.
DSSP class
Sequence position
Class prop.
1.0
0.5
0.0

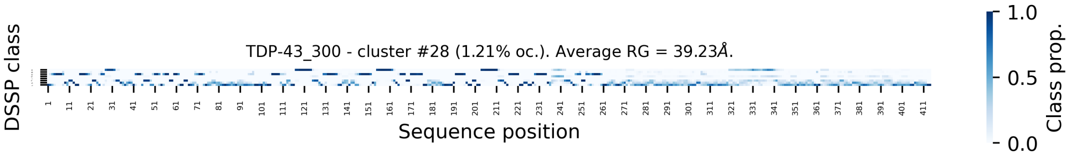
TDP-43_300 - cluster #28 (1.21% oc.). Average RG = 39.23Å.
DSSP class
Sequence position
Class prop.
1.0
0.5
0.0

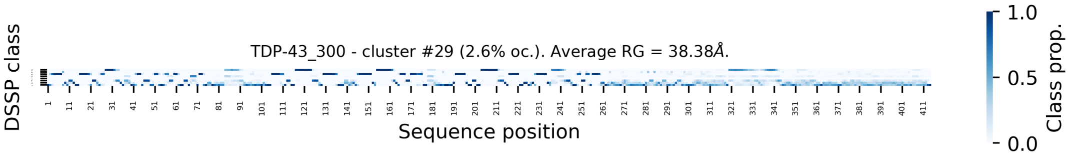
TDP-43_300 - cluster #29 (2.6% oc.). Average RG = 38.38Å.
DSSP class
Sequence position
Class prop.
1.0
0.5
0.0

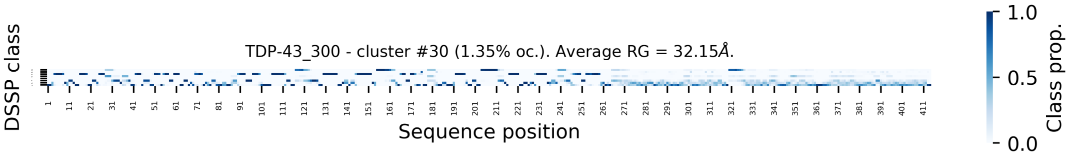
TDP-43_300 - cluster #30 (1.35% oc.). Average RG = 32.15Å.
DSSP class
Sequence position
Class prop.
1.0
0.5
0.0

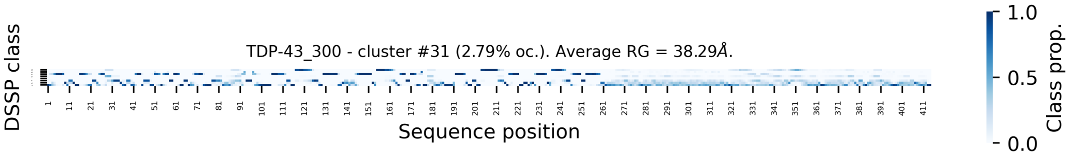
TDP-43_300 - cluster #31 (2.79% oc.). Average RG = 38.29Å.
DSSP class
Sequence position
Class prop.
1.0
0.5
0.0

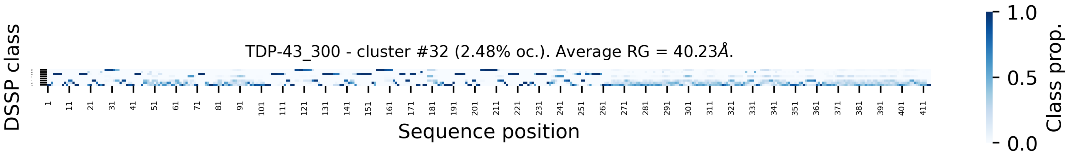
TDP-43_300 - cluster #32 (2.48% oc.). Average RG = 40.23Å.
DSSP class
Sequence position
Class prop.
1.0
0.5
0.0

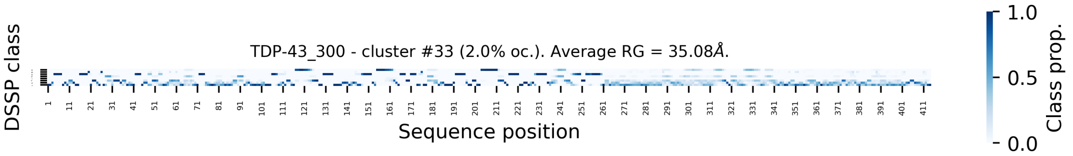
TDP-43_300 - cluster #33 (2.0% oc.). Average RG = 35.08Å.
DSSP class
Sequence position
Class prop.
1.0
0.5
0.0

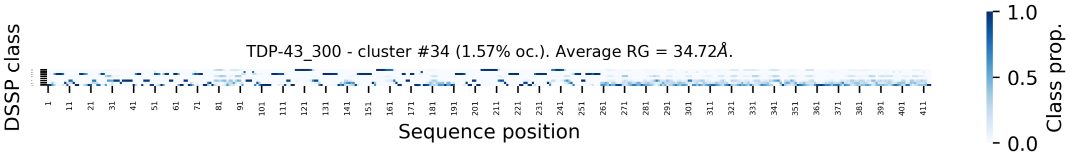
TDP-43_300 - cluster #34 (1.57% oc.). Average RG = 34.72Å.
DSSP class
Sequence position
Class prop.
1.0
0.5
0.0

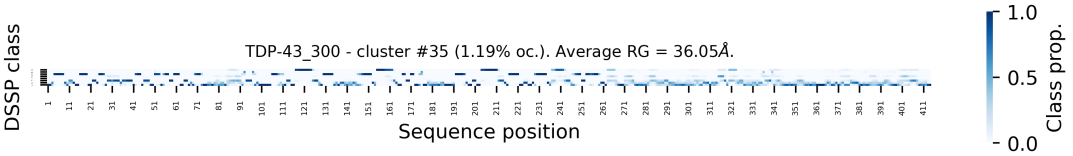
TDP-43_300 - cluster #35 (1.19% oc.). Average RG = 36.05Å.
DSSP class
Sequence position
Class prop.
1.0
0.5
0.0

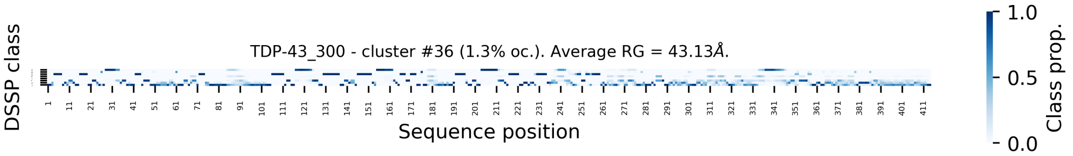

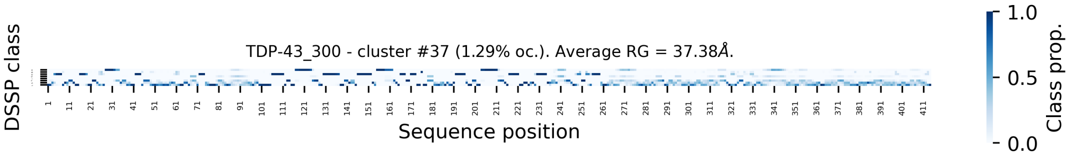

References

[1] J.-M. Chandonia, N. K. Fox, and S. E Brenner. SCOPe: classification of large macromolecular structures in the structural classification of proteins—extended database. *Nucleic Acids Research.*, 47(D1):D475–D481, 2018.

[2] M.A. Hakim Newton, Julia Rahman, Rianon Zaman, and Abdul Sattar. Enhancing protein contact map prediction accuracy via ensembles of inter-residue distance predictors. *Computational Biology and Chemistry*, 99:107700, 2022.

[3] José Ramón López-Blanco and Pablo Chacón. KORP: knowledge-based 6D potential for fast protein and loop modeling. *Bioinformatics*, 35(17):3013–3019, 2019.

[4] Rémi Zallot, Nils Oberg, and John A. Gerlt. The EFI web resource for genomic enzymology tools: Leveraging protein, genome, and metagenome databases to discover novel enzymes and metabolic pathways. *Biochemistry*, 58(41):4169–4182, 2019.

[5] Damiano Clementel, Alessio Del Conte, Alexander Miguel Monzon, Giorgia F Camagni, Giovanni Minervini, Damiano Piovesan, and Silvio C E Tosatto. RING 3.0: fast generation of probabilistic residue interaction networks from structural ensembles. *Nucleic Acids Researchearch*, 50(W1):W651–W656, 2022.

[6] John Jumper, Richard Evans, Alexander Pritzel, Tim Green, Michael Figurnov, Olaf Ronneberger, Kathryn Tunyasuvunakool, Russ Bates, Augustin Žídek, Anna Potapenko, Alex Bridgland, Clemens Meyer, Simon A. A. Kohl, Andrew J. Ballard, Andrew Cowie, Bernardino Romera-Paredes, Stanislav Nikolov, Rishub Jain, Jonas Adler, Trevor Back, Stig Petersen, David Reiman, Ellen Clancy, Michal Zielinski, Martin Steinegger, Michalina Pacholska, Tamas Berghammer, Sebastian Bodenstein, David Silver, Oriol Vinyals, Andrew W. Senior, Koray Kavukcuoglu, Pushmeet Kohli, and Demis Hassabis. Highly accurate protein structure prediction with AlphaFold. *Nature*, 596(7873):583–589, 2021.

[7] Alberto J. M. Martin, Michele Vidotto, Filippo Boscariol, Tomàs Di Domenico, Ian Walsh, and Silvio C. E. Tosatto. RING: networking interacting residues, evolutionary information and energetics in protein structures. *Bioinformatics*, 27(14):2003–2005, 2011.

[8] Javier González-Delgado, Amin Sagar, Christophe Zanon, Kresten Lindorff-Larsen, Pau Bernadó, Pierre Neuvial, and Juan Cortés. Wasco: A wasserstein-based statistical tool to compare conformational ensembles of intrinsically disordered proteins. *Journal of Molecular Biology*, 435(14):168053, 2023. Computation Resources for Molecular Biology.

[9] Leland McInnes, John Healy, and James Melville. Umap: Uniform manifold approximation and projection for dimension reduction, 2020.

[10] Leland McInnes. How umap works, 2018. Accessed: 2023-07-21.

[11] Samuel Eilenberg and Norman Steenrod. *Foundations of Algebraic Topology.* Princeton University Press, 1952.

[12] Laurens van der Maaten and Geoffrey Hinton. Visualizing data using t-sne. *Journal of Machine Learning Research*, 9(86):2579–2605, 2008.

[13] Ruizhi Xiang, Wencan Wang, Lei Yang, Shiyuan Wang, Chaohan Xu, and Xiaowen Chen. A comparison for dimensionality reduction methods of single-cell rna-seq data. *Frontiers in Genetics*, 12, 2021.

[14] Alex Diaz-Papkovich, Luke Anderson-Trocmé, Chief Ben-Eghan, and Simon Gravel. UMAP reveals cryptic population structure and phenotype heterogeneity in large genomic cohorts. *PLOS Genetics*, 15(11):e1008432, 2019.

[15] Alex Diaz-Papkovich, Shadi Zabad, Chief Ben-Eghan, Luke Anderson-Trocmé, Georgette Femerling, Vikram Nathan, Jenisha Patel, and Simon Gravel. Topological stratification of continuous genetic variation in large biobanks, 2023.

[16] Etienne Becht, Leland McInnes, John Healy, Charles-Antoine Dutertre, Immanuel W H Kwok, Lai Guan Ng, Florent Ginhoux, and Evan W Newell. Dimensionality reduction for visualizing single-cell data using UMAP. *Nature Biotechnology*, 37(1):38–44, 2018.

[17] Tim Stuart, Andrew Butler, Paul Hoffman, Christoph Hafemeister, Efthymia Papalexi, William M. Mauck, Yuhan Hao, Marlon Stoeckius, Peter Smibert, and Rahul Satija. Comprehensive integration of single-cell data. *Cell*, 177(7):1888–1902.e21, 2019.

[18] Benson Mwangi, Tian Siva Tian, and Jair C. Soares. A review of feature reduction techniques in neuroimaging. *Neuroinformatics*, 12(2):229–244, 2013.

[19] Mebarka Allaoui, Mohammed Lamine Kherfi, and Abdelhakim Cheriet. Considerably improving clustering algorithms using UMAP dimensionality reduction technique: A comparative study. In *Lecture Notes in Computer Science*, pages 317–325. Springer International Publishing, 2020.

[20] Ricardo J. G. B. Campello, Davoud Moulavi, and Joerg Sander. Density-based clustering based on hierarchical density estimates. In *Advances in Knowledge Discovery and Data Mining*, pages 160–172. Springer Berlin Heidelberg, 2013.

[21] Martin Ester, Hans-Peter Kriegel, Jörg Sander, and Xiaowei Xu. A density-based algorithm for discovering clusters in large spatial databases with noise. In *Proceedings of the Second International Conference on Knowledge Discovery and Data Mining*, KDD'96, page 226–231. AAAI Press, 1996.

[22] Rajeswari Appadurai, Jaya Krishna Koneru, Massimiliano Bonomi, Paul Robustelli, and Anand Srivastava. Clustering heterogeneous conformational ensembles of intrinsically disordered proteins with t-distributed stochastic neighbor embedding. *Journal of Chemical Theory and Computation*, 2023.

[23] Anja Conev, Mauricio Menegatti Rigo, Didier Devaurs, André Faustino Fonseca, Hussain Kalavadwala, Martiela Vaz de Freitas, Cecilia Clementi, Geancarlo Zanatta, Dinler Amaral Antunes, and Lydia E Kavraki. EnGens: a computational framework for generation and analysis of representative protein conformational ensembles. *Briefings in Bioinformatics*, 24(4):bbad242, 2023.

[24] Leland McInnes. How hdbscan works, 2016. Accessed: 2023-07-21.

[25] Lawrence Hubert and Phipps Arabie. Comparing partitions. *J. Classification*, 2(1):193–218, 1985.

[26] Frédéric Saudou and Sandrine Humbert. The biology of huntingtin. 89(5):910–926, 2016.

[27] Chiara Zuccato, Marta Valenza, and Elena Cattaneo. Molecular mechanisms and potential therapeutical targets in huntington's disease. *Physiological Reviews*, 90(3):905–981, 2010.

[28] Annika Urbanek, Matija Popovic, Anna Morató, Alejandro Estaña, Carlos A. Elena-Real, Pablo Mier, Aurélie Fournet, Frédéric Allemand, Stephane Delbecq, Miguel A. Andrade-Navarro, Juan Cortés, Nathalie Sibille, and Pau Bernadó. Flanking Regions Determine the Structure of the Poly-Glutamine in Huntingtin through Mechanisms Common among Glutamine-Rich Human Proteins. *Structure*, 28(7):733–746.e5, 2020.

[29] John B. IV Warner, Kiersten M. Ruff, Piau Siong Tan, Edward A. Lemke, Rohit V. Pappu, and Hilal A. Lashuel. Monomeric huntingtin exon 1 has similar overall structural features for wild-type and pathological polyglutamine lengths. *Journal of the American Chemical Society*, 139(41):14456–14469, 2017.

[30] Hongsuk Kang, Francisco X. Vázquez, Leili Zhang, Payel Das, Leticia Toledo-Sherman, Binquan Luan, Michael Levitt, and Ruhong Zhou. Emerging β-sheet rich conformations in supercompact huntingtin exon-1 mutant structures. *Journal of the American Chemical Society*, 139(26):8820–8827, 2017.

[31] Carlos A. Elena-Real, Amin Sagar, Annika Urbanek, Matija Popovic, Anna Morató, Alejandro Estaña, Aurélie Fournet, Christine Doucet, Xamuel L. Lund, Zhen Dan Shi, Luca Costa, Aurélien Thureau, Frédéric Allemand, Rolf E. Swenson, Pierre Emmanuel Milhiet, Ramon Crehuet, Alessandro Barducci, Juan Cortés, Davy Sinnaeve, Nathalie Sibille, and Pau Bernadó. The structure of pathogenic huntingtin exon 1 defines the bases of its aggregation propensity. *Nature Structural and Molecular Biology*, 30(3):309–320, 2023.

[32] Todd J. Cohen, Virginia M.Y. Lee, and John Q. Trojanowski. Tdp-43 functions and pathogenic mechanisms implicated in tdp-43 proteinopathies. *Trends in Molecular Medicine*, 17(11):659–667, 2011.

[33] David S.W. Protter and Roy Parker. Principles and properties of stress granules. *Trends in Cell Biology*, 26(9):668–679, 2016.

[34] Alexander E. Conicella, Gregory L. Dignon, Gül H. Zerze, Hermann Broder Schmidt, Alexandra M. D'Ordine, Young C. Kim, Rajat Rohatgi, Yuna M. Ayala, Jeetain Mittal, and Nicolas L. Fawzi. Tdp-43 α-helical structure tunes liquid–liquid phase separation and function. *Proceedings of the National Academy of Sciences*, 117(11):5883–5894, 2020.

[35] Priyesh Mohanty, Jayakrishna Shenoy, Azamat Rizuan, José F. Mercado-Ortiz, Nicolas L. Fawzi, and Jeetain Mittal. A synergy between site-specific and transient interactions drives the phase separation of a disordered, low-complexity domain. *Proceedings of the National Academy of Sciences U.S.A.*, 120(34):e2305625120, 2023.

[36] Helgi I. Ingólfsson, Azamat Rizuan, Xikun Liu, Priyesh Mohanty, Paulo C.T. Souza, Siewert J. Marrink, Michael T. Bowers, Jeetain Mittal, and Joel Berry. Multiscale simulations reveal tdp-43 molecular-level interactions driving condensation. *Biophysical Journal*, 122(22):4370–4381, 2023.

[37] Priyesh Mohanty, Azamat Rizuan, Young C. Kim, Nicolas L. Fawzi, and Jeetain Mittal. A complex network of interdomain interactions underlies the conformational ensemble of monomeric TDP-43 and modulates its phase behavior. *Protein Science*, 33(2):e4891, 2024.

[38] Malene Ringkjøbing Jensen, Klaartje Houben, Ewen Lescop, Laurence Blanchard, Rob W. H. Ruigrok, and Martin Blackledge. Quantitative conformational analysis of partially folded proteins from residual dipolar couplings: Application to the molecular recognition element of sendai virus nucleoprotein. *Journal of the American Chemical Society*, 130(25):8055–8061, 2008.

[39] Alejandro Estaña, Nathalie Sibille, Elise Delaforge, Marc Vaisset, Juan Cortés, and Pau Bernadó. Realistic ensemble models of intrinsically disordered proteins using a structure-encoding coil database. *Structure*, 27(2):381–391.e2, 2019.

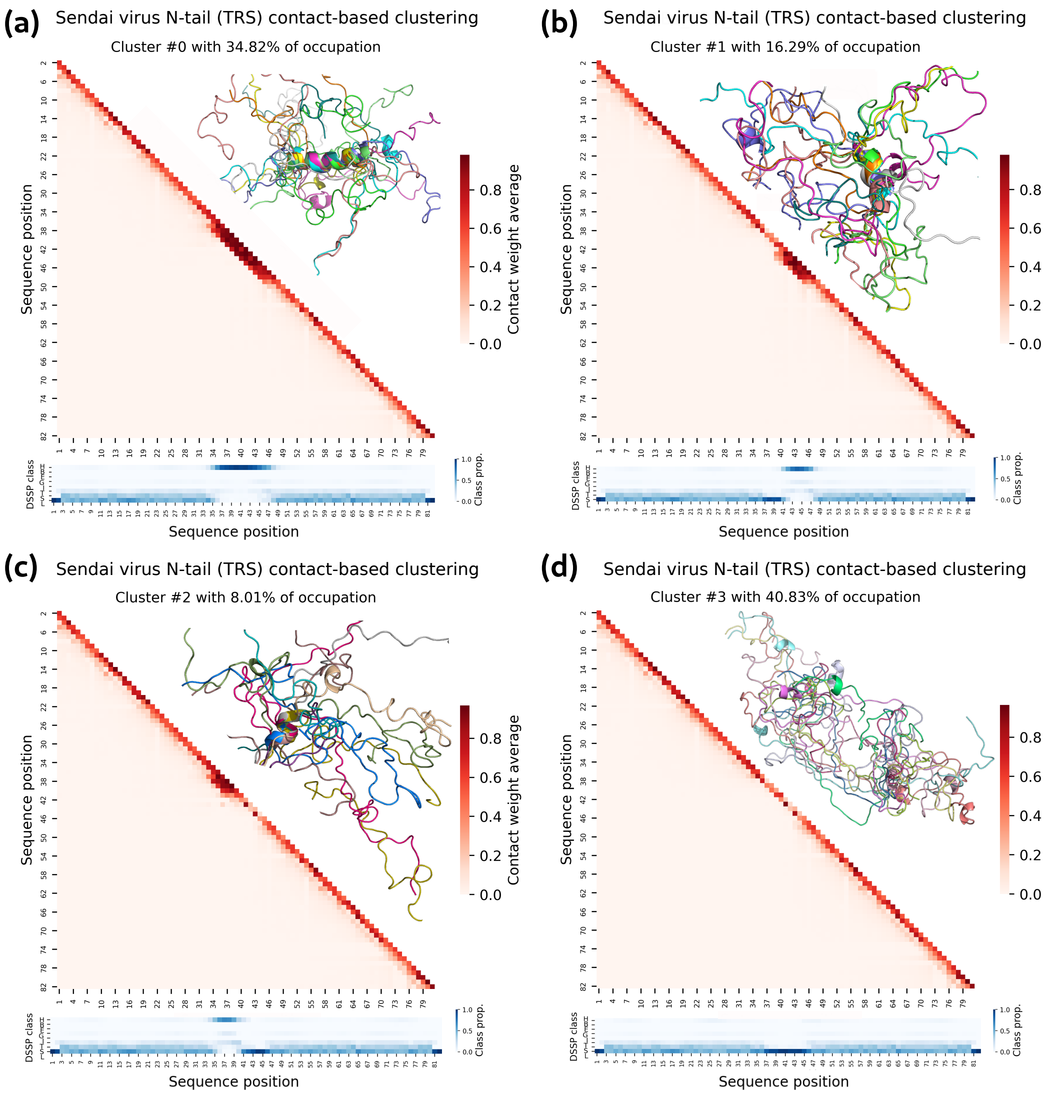

Figure S13: Cluster-specific ω-contact maps (2) for the four clusters of Sendai virus N-tails. In the upper triangles, 10 conformations randomly extracted from each clusters and aligned (a-c) at the residues presenting high helical propensity and (d) at all residues. Below each matrix, the mean DSSP propensities are presented for each cluster.

UMAP 2-dimensional projection after clustering for CHCHD4 ensemble

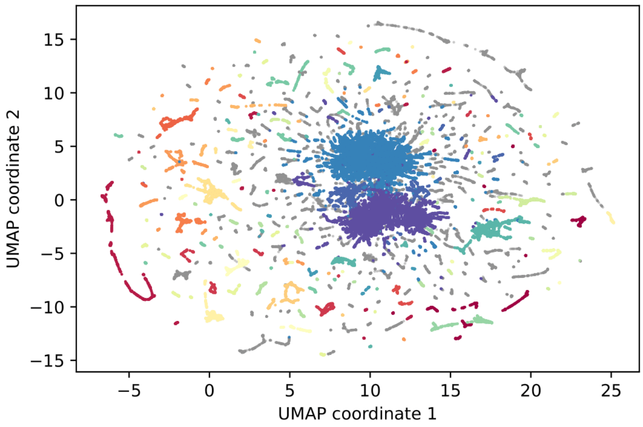

Supplement: btae627_Supplementary_Data [file btae627_supplementary_data.pdf]
